# Supplementary material for: Pre-existing anti-polyethylene glycol antibodies in pregnant women and newborns
Source: J Nanobiotechnology. 2026 Apr 18;24:512. doi: 10.1186/s12951-026-04185-9 (PMC13220525; doi:10.1186/s12951-026-04185-9)
Supplement: Supplementary file 1 — Supplementary Material 1 [file 12951_2026_4185_MOESM3_ESM.pdf]

Supplementary Appendix

Pre-existing anti-polyethylene glycol antibodies in pregnant women and newborns

Haiyang Wang[1†], Yan Feng[2†], Lin Zhang[3†], Changzheng Yuan[4], Junyang Xue[5], Jicheng Li[5], Xiao Xu[6], Wenbin Zhou[1], Baohua Li[2], Yisha Wang[1], Gan Luo[1], Yue Zheng[1], Meihua Sui[1,3,7*]

[1]*School of Basic Medical Sciences and Sir Run Run Shaw Hospital, Zhejiang University School of Medicine, Hangzhou, 310058, China*

[2]*Women's Hospital, Zhejiang University School of Medicine, Hangzhou, 310006, China*

[3]*Shaoxing People's Hospital (Shaoxing Hospital, Zhejiang University School of Medicine; The First Affiliated Hospital of Shaoxing University), Shaoxing, 312000, China*

[4]*School of Public Health, Zhejiang University School of Medicine, Hangzhou, 310058, China*

[5]*School of Basic Medical Sciences, Zhejiang University School of Medicine, Hangzhou, 310058, China*

[6]*School of Clinical Medicine, Hangzhou Medical College, Hangzhou, 311399, China*

[7]*Institue of Cell Biology, Zhejiang University, Hangzhou, 310058, China*

†These authors contributed equally

[*]Corresponding author

Meihua Sui, M.D., Ph.D. **E-mail:** suim@zju.edu.cn

Content

Methods S1. Screening of Reference Human Serum Negative for Anti-PEG Antibodies
Methods S2. Materials and Reagents Used in ELISA
Methods S3. Establishment of Detection Cutoffs of Anti-PEG IgG1-4, IgM and IgE Antibodies
Methods S4. Confirmation of the PEG Specificity of Antibodies in Serum Samples by Competitive ELISA
Methods S5. Direct ELISA for Quantification of Anti-PEG Antibodies
Results S1. Univariable Logistic Regression Analysis for the Prevalence of Pre-existing Anti-PEG Antibodies in Pregnant Women and Newborns
Results S2. Spearman Correlation Analysis for the Levels of Pre-existing Anti-PEG Antibodies in Seropositive Pregnant Women and Newborns
Results S3. Univariable Generalized Linear Regression Analysis for the Levels of Pre-existing Anti-PEG Antibodies in Seropositive Pregnant Women and Newborns
Discussion S1. Clinical Implications of Treatment-induced and Pre-existing Anti-PEG Antibodies
Table S1. Prevalence of Pre-existing Anti-PEG Antibodies in General Adults across Different Studies
Table S2. Levels of Pre-existing Anti-PEG Antibodies in Seropositive General Adults across Different Studies
Table S3. Representative PEG/PEG-derivatives Contained in Commonly Used Makeup Products
Table S4. Representative PEG/PEG-derivatives Contained in Food-Contact Packaging Materials
Table S5. Precision of Direct ELISA for Quantification of Maternal and Newborn Anti-PEG Antibodies
Figure S1. Detection of Anti-PEG IgG1 in Maternal Serum Samples by Direct ELISA (Batch 1)
Figure S2. Detection of Anti-PEG IgG1 in Maternal Serum Samples by Direct ELISA (Batch 2)
Figure S3. Detection of Anti-PEG IgG1 in Newborn Serum Samples by Direct ELISA (Batch 1)
Figure S4. Detection of Anti-PEG IgG1 in Newborn Serum Samples by Direct ELISA (Batch 2)
Figure S5. Detection of Anti-PEG IgG2 in Maternal Serum Samples by Direct ELISA (Batch 1)
Figure S6. Detection of Anti-PEG IgG2 in Maternal Serum Samples by Direct ELISA (Batch 2)
Figure S7. Detection of Anti-PEG IgG2 in Newborn Serum Samples by Direct ELISA (Batch 1)
Figure S8. Detection of Anti-PEG IgG2 in Newborn Serum Samples by Direct ELISA (Batch 2)
Figure S9. Detection of Anti-PEG IgG3 in Maternal Serum Samples by Direct ELISA (Batch 1)
Figure S10. Detection of Anti-PEG IgG3 in Maternal Serum Samples by Direct ELISA (Batch 2)
Figure S11. Detection of Anti-PEG IgG3 in Newborn Serum Samples by Direct ELISA (Batch 1)
Figure S12. Detection of Anti-PEG IgG3 in Newborn Serum Samples by Direct ELISA (Batch 2)
Figure S13. Detection of Anti-PEG IgG4 in Maternal Serum Samples by Direct ELISA (Batch 1)
Figure S14. Detection of Anti-PEG IgG4 in Maternal Serum Samples by Direct ELISA (Batch 2)
Figure S15. Detection of Anti-PEG IgG4 in Newborn Serum Samples by Direct ELISA (Batch 1)
Figure S16. Detection of Anti-PEG IgG4 in Newborn Serum Samples by Direct ELISA (Batch 2)
Figure S17. Detection of Anti-PEG IgM in Maternal Serum Samples by Direct ELISA (Batch 1)
Figure S18. Detection of Anti-PEG IgM in Maternal Serum Samples by Direct ELISA (Batch 2)
Figure S19. Detection of Anti-PEG IgM in Newborn Serum Samples by Direct ELISA (Batch 1)
Figure S20. Detection of Anti-PEG IgM in Newborn Serum Samples by Direct ELISA (Batch 2)
Figure S21. Detection of Anti-PEG IgE in Maternal Serum Samples by Direct ELISA (Batch 1)
Figure S22. Detection of Anti-PEG IgE in Maternal Serum Samples by Direct ELISA (Batch 2)
Figure S23. Detection of Anti-PEG IgE in Newborn Serum Samples by Direct ELISA (Batch 1)
Figure S24. Detection of Anti-PEG IgE in Newborn Serum Samples by Direct ELISA (Batch 2)
Table S6. Precision of Competitive ELISA for Confirmation of the PEG Specificity of Maternal and Newborn Anti-PEG IgG1-4 and IgM
Figure S25. Establishment of Specificity Cutoffs of Maternal Anti-PEG IgG1-4 and IgM
Figure S26. Establishment of Specificity Cutoffs of Newborn Anti-PEG IgG1-4 and IgM
Figure S27. Confirmation of the PEG Specificity of Anti-PEG IgG1 in Maternal Serum Samples by Competitive ELISA
Figure S28. Confirmation of the PEG Specificity of Anti-PEG IgG1 in Newborn Serum Samples by Competitive ELISA
Figure S29. Confirmation of the PEG Specificity of Anti-PEG IgG2 in Maternal Serum Samples by Competitive ELISA
Figure S30. Confirmation of the PEG Specificity of Anti-PEG IgG2 in Newborn Serum Samples by Competitive ELISA
Figure S31. Confirmation of the PEG Specificity of Anti-PEG IgG3 in Maternal Serum Samples by

Competitive ELISA
Figure S32. Confirmation of the PEG Specificity of Anti-PEG IgG3 in Newborn Serum Samples by Competitive ELISA
Figure S33. Confirmation of the PEG Specificity of Anti-PEG IgG4 in Maternal Serum Samples by Competitive ELISA
Figure S34. Confirmation of the PEG Specificity of Anti-PEG IgG4 in Newborn Serum Samples by Competitive ELISA
Figure S35. Confirmation of the PEG Specificity of Anti-PEG IgM in Maternal Serum Samples by Competitive ELISA
Figure S36. Confirmation of the PEG Specificity of Anti-PEG IgM in Newborn Serum Samples by Competitive ELISA
Figure S37. Standard Curves of Direct ELISA for Detecting Anti-PEG IgG1 in Maternal and Newborn Serum Samples
Figure S38. Standard Curves of Direct ELISA for Detecting Anti-PEG IgG2 in Maternal and Newborn Serum Samples
Figure S39. Standard Curves of Direct ELISA for Detecting Anti-PEG IgG3 in Maternal and Newborn Serum Samples
Figure S40. Standard Curves of Direct ELISA for Detecting Anti-PEG IgG4 in Maternal and Newborn Serum Samples
Figure S41. Standard Curves of Direct ELISA for Detecting Anti-PEG IgM in Maternal and Newborn Serum Samples
Figure S42. Standard Curves of Direct ELISA for Detecting Anti-PEG IgE in Maternal and Newborn Serum Samples
Table S7. Anti-PEG IgG1 Levels in Seropositive Pregnant Women
Table S8. Anti-PEG IgG1 Levels in Seropositive Newborns
Table S9. Anti-PEG IgG2 Levels in Seropositive Pregnant Women
Table S10. Anti-PEG IgG2 Levels in Seropositive Newborns
Table S11. Anti-PEG IgM Levels in Seropositive Pregnant Women
Table S12. Univariable Logistic Regression Analysis for the Prevalence of Pre-existing Total Anti-PEG Antibodies in Pregnant Women (n = 256)
Table S13. Univariable Logistic Regression Analysis for the Prevalence of Pre-existing Anti-PEG IgG and IgM in Pregnant Women (n = 256)
Table S14. Univariable Logistic Regression Analysis for the Prevalence of Pre-existing Total Anti-PEG Antibodies in Newborns (n = 256)
Figure S43. Correlation between the Levels of Pre-existing Total Anti-PEG Antibodies, Anti-PEG IgG and IgM with Maternal Age and BMI in Pregnant Women Seropositive for Anti-PEG Antibodies (n = 49), Anti-PEG IgG (n = 24) and IgM (n = 28)
Table S15. Univariable Generalized Linear Regression Analysis for the Levels of Pre-existing Total Anti-PEG Antibodies in Pregnant Women Seropositive for Anti-PEG Antibodies (n = 49)
Table S16. Univariable Generalized Linear Regression Analysis for the Levels of Pre-existing Anti-PEG IgG and IgM in Pregnant Women Seropositive for Anti-PEG IgG (n = 24) and IgM (n = 28)
Figure S44. Correlation Between the Levels of Pre-existing Total Anti-PEG Antibodies and Maternal Age, Maternal BMI, Gestational Age at Delivery, and Newborn Weight in Newborns Seropositive for Anti-PEG Antibodies (n = 14)
Table S17. Univariable Generalized Linear Regression Analysis for the Levels of Pre-existing Total Anti-PEG Antibodies in Newborns Seropositive for Anti-PEG Antibodies (n = 14)
Figure S45. Overview Diagram Describing the Design, Procedures and Major Research Findings of this Study
Table S18. Preclinical and Clinical Studies Indicating Induction of Anti-PEG Antibodies by Topical Application of Lotion Products Containing PEG Derivatives
Table S19. Representative Preclinical Studies Indicating Accelerated Blood Clearance of PEGylated Drugs
Table S20. Clinical Studies Indicating Accelerated Blood Clearance of PEGylated Drugs
Figure S46. Screening of Paired Maternal and Newborn Serum Samples Negative for Anti-PEG IgG and IgM
Figure S47. Confirmation of the Absence of Anti-PEG IgE in Maternal and Newborn Serum Samples Negative for Anti-PEG IgG and IgM
References

Methods S1. Screening of Reference Human Serum Negative for Anti-PEG Antibodies

When quantitatively detecting anti-PEG antibodies in serum using direct ELISA, the presence of matrix in the serum can interfere with the detection of anti-PEG antibodies, impacting the accuracy of the results, known as matrix effects (1). To mitigate these effects and ensure the precision of our assays, we used a standard dilution buffer (10% reference human serum negative for anti-PEG antibodies, 2% (w/v) skim milk powder in DPBS) to create serial dilutions of human anti-PEG antibody standards (see Methods). To prepare the standard dilution buffer, commercial human anti-PEG IgG ELISA kits and human anti-PEG IgM ELISA kits were used to screen a batch of paired maternal and newborn serum samples that were negative for anti-PEG IgG and IgM according to the manufacturer's instructions.

Lacking commercial kits for detecting anti-PEG IgE, we intend to employ competitive ELISA (see below eMethods 4 for detailed experimental procedures) to confirm the presence of anti-PEG IgE in sera negative for anti-PEG IgG and IgM measured by commercial kits. In this experiment, anti-PEG IgG and IgM-negative maternal and newborn pooled serum samples diluted 1:5 with dilution buffer (2% (w/v) skim milk powder in DPBS) were used as the tested samples. Six serial dilutions of human anti-PEG IgE standards (respectively at 8.2, 24.6, 74.0, 222.2, 666.6 and 2000.0 ng/mL) in standard dilution buffer (20% maternal or newborn pooled serum tested negative for anti-PEG IgG and IgM, 2% (w/v) skim milk powder in DPBS) were used as the positive control.

Methods S2. Materials and Reagents Used in ELISA

NH_2-PEG_{10000}-NH_2 (Cat. No. 8218815000) was obtained from Sigma-Aldrich (St. Louis, MO, USA). 3-[(3-cholamidopropyl)dimethylammonio]-1-propanesulfonate (CHAPS, Cat. No. ST1145), 3,3′,5,5′-tetramethylbenzidine dihydrochloride hydrate (TMB 2HCl, Cat. No. ST1708) and nonfat powdered milk (Cat. No. P0216) were purchased from Beyotime Biotechnology (Shanghai, China). Maxisorp 96-well microplates (Cat. No. 44-2404-21) were acquired from Nalge-Nunc International (Rochester, NY, USA). Chimeric human anti-PEG IgG1 (Cat. No. cHu-3.3-IgG1, Clone No. c3.3-IgG1), chimeric human anti-PEG IgG2 (Cat. No. cHu-r33G-IgG2, Clone No. cr33G-IgG2), chimeric human anti-PEG IgG3 (Cat. No. cHu-r33G-IgG3, Clone No. cr33G-IgG3), chimeric human anti-PEG IgG4 (Cat. No. cHu-3.3-IgG4, Clone No. c3.3-IgG4), chimeric human anti-PEG IgM (Cat. No. cHu-AGP4-PABG-A, Clone No. cAGP4-IgM) and human anti-PEG IgE (Cat. No. Hu-6.3-IgE, Clone No. Hu 6.3-IgE) were obtained from Academia Sinica (Taipei, China). Mouse anti-human IgG1 Fc secondary antibody (Cat. No. 9054-05, Clone No. HP6001), mouse anti-human IgG2 Fc secondary antibody (Cat. No. 9060-05, Clone No. 31-7-4), and mouse anti-human IgG3 Hinge secondary antibody (Cat. No. 9210-05, Clone No. HP6050) were purchased from Southern Biotech (Birmingham, AL, USA). Goat anti-human IgM μ-chain secondary antibody (Cat. No. 609-1307) was acquired from Rockland Immunochemicals (Philadelphia, Pennsylvania, USA). Mouse anti-human IgE secondary antibody (Cat. No. SA5-10306, Clone No. 24A) and mouse anti-human IgG4 Fc secondary antibody (Cat. No. A-10654, Clone No. HP6025) were obtained from ThermoFisher Scientific (Waltham, MA, USA). Human anti-PEG IgM ELISA kit (Cat. No. PEG-020), and human anti-PEG IgG ELISA kit (Cat. No. PEG-010) were purchased from Alpha Diagnostic Intl (San Antonio, Texas, USA). LIBOD® (PEGylated liposomal doxorubicin) was acquired from Shanghai Fudan-zhangjiang Bio-Pharmaceutical Co. Ltd (Shanghai, China).

Methods S3. Establishment of Detection Cutoffs of Anti-PEG IgG1-4, IgM and IgE Antibodies

Detection cutoffs of anti-PEG IgG1-4, IgM and IgE antibodies were established based on the absorbance value of six negative controls (standard dilution buffer: 10% human reference serum negative for anti-PEG antibodies, 2% (w/v) skim milk powder in DPBS) in each batch of the direct ELISA according to the method described by Frey et al (2). The formula for Detection cutoff is given below: Detection cutoff = $\bar{X} + SDf$, where $\bar{X}$ is the mean absorbance value of six independent negative controls, SD is the standard deviation of absorbance value of six independent negative controls, f is the standard deviation multipliers based on the number of negative controls (n = 6) and the 95% confidence level.

Methods S4. Confirmation of the PEG Specificity of Antibodies in Serum Samples by Competitive ELISA

Serum samples with average absorbance values higher than the corresponding detection cutoffs in the direct ELISA were further verified by a competitive ELISA to confirm the PEG specificity of the antibodies detected. The competitive ELISA experiment was performed based on a previously published procedure from Chen et al (1). Maxisorp 96-well microplates were prepared following the same procedure as direct ELISA (see Methods). After incubation with blocking buffer (5% (w/v) skim milk powder in DPBS, 200 μL/well) at RT for 5.5 hours, plates were gently washed three times with 350 μL of DPBS. Dilution buffer with competitors was prepared by dissolving clinically relevant PEGylated liposomal doxorubicin (the PEGylated liposomal doxorubicin (LIBOD®), congruent with the formulation of Doxil®, exhibits stable physicochemical characteristics; Given the robust binding affinity of pre-existing anti-PEG antibodies to clinically relevant PEGylated liposomal doxorubicin (1), we have elected this formulation as a competitor in our study) into dilution buffer (2% (w/v) skim milk powder in DPBS) at a concentration of 400 mg phospholipids/mL. Then 50 μL of dilution buffer with competitors or dilution buffer without competitors were added into plates and further incubated for 15 minutes at RT. Following this, 50 μL of anti-PEG antibody-positive serum samples diluted 1:25 with dilution buffer, together with eight serial dilutions of human anti-PEG IgG1-4 and IgM standards (respectively at 2.3, 6.9, 20.6, 61.7, 185.2, 555.6, 1666.7 and 5000.0 ng/mL) in standard dilution buffer (4% human reference serum tested negative for anti-PEG antibodies, 2% (w/v) skim milk powder in DPBS), were added to the wells containing PEGylated liposomes (competitors) or no PEGylated liposomes of corresponding detection plates in duplicate (for serum samples) or triplicate (for anti-PEG antibody standards). Same volumes of triplicate standard dilution buffer were used as negative controls. After further incubation for 1 hour at RT, the same procedure was followed as direct ELISA (see Methods).

Specificity cutoffs of anti-PEG IgG1-4 and IgM antibodies were established based on the absorbance value of anti-PEG antibody standards with the addition of competitors and without the addition of competitors according to the method described by Chen et al (1). Specifically, the percentage of antibody binding for anti-PEG antibody standards with the addition of competitors and without the addition of competitors was calculated separately by dividing the absorbance value of anti-PEG antibody standards with the addition of competitors and without the addition of competitors by the average absorbance value of those without the addition of

competitors. Specificity cutoffs of anti-PEG antibodies were defined as the maximum percentage of antibody binding for anti-PEG antibody standards with the addition of competitors (NOTE: The percentage of antibody binding for anti-PEG antibody standards with the addition of competitors must be significantly less than the percentage of antibody binding for those without the addition of competitors). The percentage of antibody binding for serum samples with the addition of competitors and without the addition of competitors was calculated using the same method as that of antibody binding for anti-PEG antibody standards. Antibodies detected in serum samples via direct ELISA are considered anti-PEG antibodies (antibodies with PEG specificity) if the percentage of antibody binding for serum samples with the addition of competitors was significantly less than that of antibody binding for serum samples without the addition of competitors and no more than the specificity cutoffs of corresponding anti-PEG antibodies.

Methods S5. Direct ELISA for Quantification of Anti-PEG Antibodies

Anti-PEG IgG1-4, IgM and IgE standard curves in each batch of the direct ELISA were constructed by plotting the average absorbance values (OD450 nm) and corresponding antibody concentrations with Four Parameter Logistic (4PL) curve fit using Origin 2021 software (OriginLab Corporation, Northampton, Massachusetts, USA). The goodness of fit of each standard curve was evaluated by the coefficient of determination (R^2). To evaluate the quality control of ELISA, intra-assay precision was determined by calculating the Coefficient of Variation (CV% = (Standard deviation/Mean) × 100%) for all detectable standards and samples in all batches of ELISA (3). In addition, inter-assay precision was determined by calculating the Coefficient of Variation for serially diluted anti-PEG antibody standards among all batches of ELISA (3). The acceptance criteria for ELISA's mean intra-assay and inter-assay coefficient of variation (CV%) are < 20% and < 25%, respectively (3).

Results S1. Univariable Logistic Regression Analysis for the Prevalence of Pre-existing Anti-PEG Antibodies in Pregnant Women and Newborns

To reveal the demographic and clinical factors associated with the prevalence of maternal and newborn anti-PEG antibodies, univariable logistic regression analysis was firstly conducted. Interestingly, our data showed that for each 1-year increase in the age of pregnant women, the prevalence of maternal total anti-PEG antibodies and anti-PEG IgM respectively dropped to 88.7% (95% CI, 0.820-0.959; $P = 0.003$; Table S12) and 88.6% (95% CI, 0.783-0.958; $P = 0.005$; Table S13). Compared with those without cosmetic use (reference category with odds ratio/OR = 1), the odds of having maternal anti-PEG IgG and having newborn total anti-PEG antibodies were respectively 5.841 (95% CI, 1.947-17.558; $P = 0.002$; Table S13) and 7.259 (95% CI, 2.159-24.411; $P = 0.001$; Table S14) times higher for pregnant women with 4-7 days per week cosmetic use. Moreover, compared with those without take-out food consumption (reference category with OR = 1), the odds of having maternal total anti-PEG antibodies were 3.129 (95% CI, 1.195-8.191; $P = 0.020$), 2.987 (95% CI, 1.071-8.328; $P = 0.036$) and 4.148 (95% CI, 1.238-13.901; $P = 0.021$) times higher, respectively, for pregnant women who had take-out food 1-3 times, 4-6 times and 7-9 times per week (Table S12). Meanwhile, compared with those without take-out food consumption (reference category with OR = 1), the odds of having maternal anti-PEG IgG were 8.500 (95% CI, 1.531-47.182; $P = 0.014$) for pregnant women who had take-out food 7-9 times per week (Table S13). These data revealed inverse associations of the prevalence of maternal total anti-PEG antibodies and anti-PEG IgM with maternal age, while positive associations of the prevalence of maternal total anti-PEG antibodies and anti-PEG IgG with maternal take-out food consumption, as well as a positive association of the prevalence of maternal anti-PEG IgG and newborn total anti-PEG antibodies with maternal cosmetic use. Nevertheless, no other association was found between the prevalence of maternal or newborn anti-PEG antibodies (either total or any isotype) and all other demographic or clinical factors (Tables S12-14). Variables positive in univariable logistic regression analysis, including maternal age and maternal take-out food consumption, were entered into a multivariable logistic regression analysis to estimate adjusted associations with the prevalence of maternal total anti-PEG antibodies. Moreover, variables positive in univariable logistic regression analysis, including maternal cosmetic use and maternal take-out food consumption, were entered into a multivariable logistic regression analysis to estimate adjusted associations with the prevalence of maternal anti-PEG IgG.

Results S2. Spearman Correlation Analysis for the Levels of Pre-existing Anti-PEG Antibodies in Seropositive Pregnant Women and Newborns

For pregnant women and newborns seropositive for anti-PEG antibodies, the Spearman correlation analysis was conducted to evaluate the correlation between the levels of maternal and newborn anti-PEG antibodies after $\log_{10}$ transformation and each continuous variable. Correlation plots showed a moderate positive correlation between the levels of maternal total anti-PEG antibodies and maternal age ($r = 0.363$, $P = 0.01$; Figure S43A), as well as a higher positive correlation between the levels of newborn total anti-PEG antibodies and maternal age ($r = 0.711$, $P = 0.006$; Figure S44A). Nevertheless, no correlation was found between the levels of maternal total anti-PEG antibodies and maternal BMI (Figure S43B), as well as between the levels of maternal anti-PEG IgG and IgM with both maternal age and BMI (Figure S43C-F). Nor did we find any correlation between the levels of newborn total anti-PEG antibodies and maternal BMI, gestational age at delivery and newborn weight (Figure S44B-D).

Results S3. Univariable Generalized Linear Regression Analysis for the Levels of Pre-existing Anti-PEG Antibodies in Seropositive Pregnant Women and Newborns

For pregnant women and newborns seropositive for anti-PEG antibodies, to reveal the demographic and clinical factors associated with the levels of maternal and newborn anti-PEG antibodies after $\log_{10}$ transformation, univariable generalized linear regression analysis was firstly conducted. Univariable analysis showed that for each 1-year increase in the age of pregnant women, the levels of maternal total anti-PEG antibodies, maternal anti-PEG IgG, and newborn total anti-PEG antibodies increased by 0.053 (95% CI, 0.018-0.088; $P = 0.003$; Table S15), 0.057 (95% CI, 0.010-0.104; $P = 0.018$; Table S16) and 0.065 (95% CI, 0.032-0.098; $P < 0.001$; Table S17), respectively. Compared with those with vocational degree or below, the levels of maternal anti-PEG IgM increased by 0.453 (95% CI, 0.005-0.900; $P = 0.047$; Table S16) for pregnant women with bachelor's degree. Compared with no cosmetic use, the levels of maternal total anti-PEG antibodies and anti-PEG IgM increased by 0.576 (95% CI, 0.191-0.960; $P = 0.003$; Table S15) and 1.220 (95% CI, 0.645-1.795; $P < 0.001$; Table S16), respectively, for pregnant women with cosmetic use 4-7 days per week. Moreover, compared with those without take-out food consumption, the levels of maternal anti-PEG IgG increased by 0.702 (95% CI, 0.107-1.297; $P = 0.021$) and 0.642 (95% CI, 0.006-1.279; $P = 0.048$), respectively, for pregnant women who had take-out food 1-3 times and 7-9 times per week (Table S16). These data demonstrate positive associations between the levels of maternal total anti-PEG antibodies, anti-PEG IgG and newborn total anti-PEG antibodies with maternal age, a positive association between the levels of maternal anti-PEG IgG and maternal take-out food consumption, as well as a positive association between the levels of maternal total anti-PEG antibodies and anti-PEG IgM with maternal cosmetic use. Nevertheless, no other association was found between the levels of maternal or newborn anti-PEG antibodies (either total or any isotype) and all other demographic or clinical factors (Tables S15-17). Variables positive in univariable generalized linear regression analysis, including maternal age and maternal cosmetic use, were entered into a multivariable generalized linear regression analysis to estimate adjusted associations with the levels of maternal total anti-PEG antibodies after $\log_{10}$ transformation among seropositive pregnant women. Moreover, variables positive in univariable generalized linear regression analysis, including maternal age and maternal take-out food consumption, were entered into a multivariable generalized linear regression analysis to estimate adjusted associations with the levels of maternal anti-PEG IgG after $\log_{10}$ transformation among anti-PEG IgG seropositive pregnant women. Lastly, variables positive in univariable generalized linear regression analysis, including maternal education and maternal cosmetic use, were entered into a multivariable generalized linear regression analysis to estimate adjusted associations with the levels of maternal anti-PEG IgM after $\log_{10}$ transformation among anti-PEG IgM seropositive pregnant women.

Discussion S1. Clinical Implications of Treatment-induced and Pre-existing Anti-PEG Antibodies

To date, researchers have confirmed that in rats (4-8), mice (6,7,9,10), rabbits (7), beagle dogs (6) and guinea pigs (7), anti-PEG antibodies induced by previous intravenous injection of PEGylated liposomes led to accelerated blood clearance (ABC) phenomenon in subsequent or repeated injections of same liposomes (Table S18). Moreover, this anti-PEG antibody-mediated ABC phenomenon were also observed for intravenously injected PEGylated proteins (11-13), PEGylated polymer nanoparticles (14), PEGylated micelles (15), PEGylated nanoemulsions (16), PEGylated exosomes (17) and PEGylated lipid nanoparticles in animal models (Table S18). Interestingly, ABC phenomenon is not confined to intravenous administration of PEGylated agents. For instance, Elsadek et al. reported that ABC phenomenon occurred following repeated subcutaneous injections of Peginterferon Alfa-2a (Pegasys®) (12) in mice. Most recently, we demonstrated the presence of accelerated blood clearance of the PEGylated lipid nanoparticles of mRNA vaccine Comirnaty® following repeated intramuscular injections in rats (3).

Importantly, several clinical studies have documented the occurrence of the ABC phenomenon in patients following treatment with FDA-approved PEGylated therapeutics (18-25) (Table S19). For instance, in several clinical studies, both subcutaneous and intravenous injections of Krystexxa® (PEGylated uricase) induced the production of anti-PEG antibodies in gout patients, which resulted in significantly increased drug clearance rates and reduced therapeutic effecticacy (18, 20-22). Impressively, Armstrong et al. discovered that Oncaspar® (PEGylated asparaginase) induced anti-PEG antibodies in pediatric patients with acute lymphoblastic leukemia (ALL), leading to ABC phenomenon and reduced drug efficacy (19). Moreover, in a Phase II clinical trial, Zori et al. found that after intravenous infusion of Palynziq® (PEGylated phenylalanine ammonia lyase), approximately 90% of phenylketonuria patients were seropositive for anti-PEG IgG or anti-PEG IgM, which was correlated with augmented drug clearance rates (23). Stephen et al. discovered that intramuscular injections of Spikevax® (a PEGylated lipid nanoparticle-encapsulated mRNA encoding the spike protein of SARS-CoV-2 for the prevention of COVID-19) induced the production of anti-PEG antibodies, which was significantly correlated with increased drug clearance rates (25). It is noteworthy that similar to treatment-induced anti-PEG antibodies, several clinical investigations have delineated pre-existing anti-PEG antibody-mediated alterations in pharmacokinetic profiles of PEGylated therapeutics (21,24). For example, in pediatric subjects undergoing treatment with Oncaspar® for ALL, the presence of pre-existing anti-PEG antibodies was associated with an enhanced systemic clearance of the initially administered drug and a subsequently diminished therapeutic efficacy (24). Analogously, in individuals seropositive for pre-existing anti-PEG antibodies, there were pronounced reductions in both the plasma concentration and therapeutic efficacy of Krystexxa® (21).

On the other hand, Kozma et al. revealed that anti-PEG IgM could activate the complement system via the classical pathway, causing severe hypersensitivity reactions to PEGylated liposomes in pigs (26). Later, in mice models, Stavnsbjerg et al. identified a correlation between acute hypersensitivity reactions and production of anti-PEG IgG following repeated intravenous administrations of PEGylated liposomes containing Toll-like receptor (TLR7/8) agonists (27), while Chen et al. found that anti-PEG IgG could activate innate immune cells and induce hypersensitivity reactions to PEGylated liposomes (28). Notably, Kozma and colleagues linked higher rates of allergic reactions in women to the presence of pre-existing anti-PEG antibodies (29). In addition, pre-existing anti-PEG IgG and anti-PEG IgM were correlated with hypersensitivity reactions in pediatric patients with ALL following the administration of Oncaspar® (24). Indeed, pre-existing anti-PEG antibodies were associated with severe first-exposure hypersensitivity reactions to PEGylated RNA aptamer (Pegnivacogin), which led to the discontinuation of phase III clinical trials (30).

Table S1. Prevalence of pre-existing anti-PEG antibodies in general adults across different studies

Year[Ref]	Population location	Sample number	Gender (female/male)	Age, range, (y)	Prevalence of anti-PEG antibodies: Total[a]	IgM	IgG	IgM & IgG	IgE	Correlations between the prevalence of anti-PEG antibodies and demographic characteristics	Method
1984 (31)	Japan GER Italy	Japan: 142 GER: 151 Italy: 160	NA	NA	0.2%	NA	NA	NA	NA	NA	Passive hemagglutination
2016 (32)	Austria	710	NA	18-67	23%	13%	15%	5%	NA	IgG prevalence was negatively correlated with donor age; IgM prevalence showed no correlation with donor age.	Flow cytometry with validation assays
	Austria USA	Austria:100 USA: 500	NA	18-67	24%	14%	12%	2%	NA	IgG prevalence was negatively correlated with donor age, and was correlated with donor location; IgM prevalence showed no correlation with either donor age or location.	Direct ELISA & competitive ELISA
2016 (1)	Taiwan, China	1504	748/756	20-80	44.3%	27.1%	25.7%	8.4%	NA	Females have a higher prevalence of IgM (32.0% vs 22.2%, $p < 0.0001$) and IgG (28.3% vs 23.0%, $p = 0.018$) than males; IgG prevalence showed negative correlations with donor age.	Direct ELISA & competitive ELISA
2016 (33)	USA	377	151/226	19-70	72%	55%	48% (IgG1：26%；IgG2：57%；IgG3：1%；IgG4: 1%)	30%	NA	IgG prevalence was negatively correlated with donor age; IgM prevalence showed no correlation with donor age; no correlation was observed between donor gender or race and the prevalence of either IgM or IgG.	Competitive ELISA with validation assays
	USA	1970s: 30 1980s: 30 1990s: 19	1970s: 15/15 1980s: 15/15 1990s: 6/13	19-70	56%	35%	36%	16%	NA	NA	Competitive ELISA with validation assays
2021 (34)	USA	300	116/184	18-65	65.3%	44%	46.3%	25%	NA	No correlation between donor age, gender or race and the prevalence of either IgM or IgG.	Flow cytometry with validation assays
2023 (35)	Germany	500	NA	18-60	83%	NA	NA	48.8%	NA	IgM and IgG prevalence was negatively correlated with donor age.	Direct ELISA & competitive ELISA
2024 (36)	USA	12	NA	NA	NA	83%	83%	NA	0%	NA	Competitive ELISA
2024 (Present study)	Zhejiang, China	Pregnant woman: 256	NA	23-44	19.14%	10.94%	9.38% (IgG1：2.34%；IgG2：7.03%；IgG3 & IgG4：0%)	1.17%	0%	Maternal age (inverse association) and take-out food consumption (positive association) were independent influencing factors of the prevalence of maternal total antibodies; maternal age (inverse association) was an independent influencing factor of the prevalence of maternal IgM; maternal cosmetic use (positive association) and take-out food consumption (positive association) were independent influencing factors of the prevalence of maternal IgG; no other association was found between the prevalence of maternal antibodies (either total or any isotype) and all other demographic factors.	Direct ELISA & competitive ELISA
	Zhejiang, China	Newborns: 256	129/127	NA	5.47%	0%	5.47% (IgG1：2.73%；IgG2：2.73%；IgG3 & IgG4：0%)	0%	0%	A positive association between total newborn antibody prevalence and maternal cosmetic use was observed; no other association was found between total newborn antibody prevalence and all other demographic factors.	Direct ELISA & competitive ELISA

Abbreviations: GER, Germany; NA, not applicable. [a]Total Ab included all anti-PEG antibody isotypes.

Table S2. Levels of pre-existing anti-PEG antibodies in seropositive general adults across different studies

Year[Ref]	Levels (ng/mL) of anti-PEG antibodies				Correlations between the levels of anti-PEG antibodies and demographic characteristics	Detection cutoffs
	Total[a]	IgM	IgG	IgE		
1984 (31)	NA	NA	NA	NA	NA	NA
2016 (32)	NA	NA	NA	NA	NA	1:20 titer[b]
	NA	NA	NA	NA	NA	1:20 titer[c]
2016 (1)	NA	Range: 300 - 238000;<br>Mean: 5760;<br>**Median: 1790**	Range: 100 - 57300;<br>Mean: 1780;<br>**Median: 960**	NA	IgG levels were negatively correlated with donor age; IgM levels showed no correlation with donor age; neither IgM nor IgG levels were associated with donor gender.	NA[d]
2016 (33)	NA	Range: 0.0 - 2858.4;<br>**Median: 19.5**	Range: 0.0 - 6488.6;<br>**Median: 49.3**	NA	IgG levels were inversely related to donor age, while IgM levels showed no such link; females had relatively higher IgM levels, but gender didn't affect IgG levels; neither IgM nor IgG levels were associated with donor race.	14.2, 15.1, 3.9, 4.4 and 6.4 ng/mL for anti-PEG IgG1, IgG2, IgG3, IgG4 and IgM, respectively[f]
	NA	Range: 0.0 - 101.2 for 1970s<br>**Median: 9.1 for 1970s**<br>Range: 0.0 - 233.9 for 1980s<br>**Median: 14.0 for 1980s**<br>Range: 0.0 - 874.6 for 1990s<br>**Median: 33.2 for 1990s**	Range: 0.0 - 2685.9 for 1970s<br>**Median: 31.6 for 1970s**<br>Range: 0.0 - 2071.9 for 1980s<br>**Median: 12.6 for 1980s**<br>Range: 0.0 - 3504.2 for 1990s<br>**Median: 25.3 for 1990s**	NA	NA	
2021 (34)	NA	Range: 26 - 11604;<br>Mean: 930;<br>**Median: 310**	Range: 39 - 18713;<br>Mean: 880;<br>**Median: 260**	NA	No correlation between donor age, gender or race and the levels of either IgM or IgG.	35 and 25 ng/mL for anti-PEG IgG and IgM, respectively[g]
2023 (35)	NA	NA	NA	NA	No correlation between donor age and the levels of either IgM or IgG.	NA[h]
2024 (36)	NA	NA	NA	NA	NA	NA[i]
2024<br>(Present study)	Pregnant woman<br>Range: 55.43 - 23649.14;<br>Mean: 941.67;<br>**Median: 292.02**	Pregnant woman<br>Range: 55.43 - 23649.14;<br>Mean: 1068.95;<br>**Median: 175.07**	Pregnant woman<br>Range: 75.54 - 2604.89;<br>Mean: 675.47;<br>**Median: 495.41**	Pregnant woman<br>Range: 0 - 0;<br>Mean: 0;<br>**Median: 0**	Maternal age (positive association) and cosmetic use (positive association) were independent influencing factors of maternal total antibody levels; maternal cosmetic use (positive association) was an independent influencing factor of maternal IgM levels; Maternal age (positive association) and take-out food consumption (positive association) were independent influencing factors of maternal total IgG levels; no other association was found between the levels of maternal antibodies (either total or any isotype) and all other demographic factors.	14.7, 4.4, 0.3, 1.2, 1.2 and 7.7 ng/mL for anti-PEG IgG1, IgG2, IgG3, IgG4, IgM and IgE, respectively[j]
	Newborns<br>Range: 100.24 - 1513.98;<br>Mean: 443.27;<br>**Median: 306.22**	Newborns<br>Range: 0 - 0;<br>Mean: 0;<br>**Median: 0**	Newborns<br>Range: 100.24 - 1513.98;<br>Mean: 443.27;<br>**Median: 306.22**	Newborns<br>Range: 0 - 0;<br>Mean: 0;<br>**Median: 0**	Maternal age (positive association) was an independent influencing factor of total newborn antibody levels; no other association was found between total newborn antibody levels and all other demographic factors.	11.4, 4.3, 0.2, 0.9, 0.1 and 9.4 ng/mL for anti-PEG IgG1, IgG2, IgG3, IgG4, IgM and IgE, respectively[j]

Abbreviations: NA, not applicable; OD, absorbance values.

[a]Total Ab included all anti-PEG antibody isotypes.

[b]The study employed flow cytometry without constructing a standard curve to semi-quantitatively (a method between qualitative and quantitative) evaluate anti-PEG antibodies in serum samples by determining the antibody titer via serial dilution, with a titer of 1:20 defined as the detection cutoff. The antibody titer, typically expressed as "1:X," indicates the maximum dilution at which the antibody can be detected. For instance, a titer of 1:32,000 means the antibody is still detectable at a 32,000-fold dilution but not at a higher dilution.

[c]The study utilized ELISA without constructing a standard curve to semi-quantitatively assess anti-PEG antibodies in serum samples by determining the antibody titer through serial dilution. An antibody titer of 1:20 was set as the detection cutoff.

[d]The study employed a quantitative direct ELISA, which requires the construction of a standard curve, to measure the relative concentration of anti-PEG antibodies in serum samples. The article provides the formula for calculating the detection cutoff as $OD_{sample} \geq 3 \times$ mean $OD_{background}$, but does not specify an exact value for the detection cutoff.

[e]The study delineates the distribution of anti-PEG antibody levels across the entire spectrum of the healthy human population, encompassing both individuals with detectable levels of anti-PEG antibodies (anti-PEG antibody-positive) and those without such detectable levels (anti-PEG antibody-negative).

[f]The study used a quantitative competitive ELISA, which involves creating a standard curve, to determine the relative concentration of anti-PEG antibodies in serum samples. The calculation for the detection cutoff is detailed as $\bar{X} + \mathrm{SD}f$ (2),

where $\overline{X}$ is the mean absorbance of negative controls, SD is their standard deviation, and f is a multiplier based on the number of negative controls and a 95% confidence level (for details, see eReference 2). Additionally, specific detection cutoff values are provided within the text.

[g]The study utilized flow cytometry, which required the construction of a standard curve, to assess the relative concentration of anti-PEG antibodies in serum samples. Although the calculation formula for the detection cutoff was not provided, specific values were given within the text.

[h]The study employed a quantitative direct ELISA, which requires the construction of a standard curve, to measure the relative concentration of anti-PEG antibodies in serum samples. The article provides the formula for calculating the detection cutoff as $OD_{sample} \geq$ mean $OD_{background} + 3 \times SD_{background}$, but does not specify an exact value for the detection cutoff.

[i]The study employed a quantitative competitive ELISA, which requires the construction of a standard curve, to measure the relative concentration of anti-PEG antibodies in serum samples. The article provides the formula for calculating the detection cutoff as $OD_{sample} \geq$ mean $OD_{background} + 10 \times SD_{background}$, but does not specify an exact value for the detection cutoff.

[j]The present study used a quantitative direct ELISA, which involves creating a standard curve, to determine the relative concentration of anti-PEG antibodies in serum samples. The calculation for the detection cutoff is detailed as $\overline{X} + \mathrm{SD}f$ (2), where $\overline{X}$ is the mean absorbance of negative controls, SD is their standard deviation, and f is a multiplier based on the number of negative controls and a 95% confidence level (for details, see eMethods 3). Additionally, specific detection cutoff values are provided within the text.

Table S3. Representative PEG/PEG-derivatives contained in commonly used makeup products (defined as "cosmetics" in the questionnaire interview of this study)

Category	PEG/PEG-derivatives (INCI)	Function	References
Liquid foundation	PEG-12 dimethicone, PEG/PPG-silicone copolymers (e.g. PEG/PPG-18/18 Dimethicone)	Silicone-PEG surfactants: improve spread, slip, and emulsion stability; help disperse UV filters/pigments	(37)
Mascara, eyeliner	PEG-Stearates (e.g. PEG-45/75 Stearate)	Nonionic emulsifiers in eye-area formulations	(38)
Blemish balm Cream, color correcting cream	PEG diesters (e.g. PEG-6/-8/-12 Distearate, PEG-12 Dilaurate)	Emulsifiers/surfactants; thickening and texture modification in oil-rich systems	(39)

INCI，International Nomenclature of Cosmetic Ingredients.

Table S4. Representative PEG/PEG-derivatives contained in food contact packaging materials

Category	CAS No.	Function	References
PEG Dilaurate	9005-02-1	Defoaming Agent & Plasticizer: Used in the manufacture of paper and paperboard intended for food contact (e.g., pizza boxes, paper bowls).	(40)
PEG Distearate	9005-08-7	Lubricant & Release Agent: Used to prevent food from sticking to packaging surfaces; additive in plastic films.	(41)
PEG Mono-oleate /Dioleate	9004-96-0	Emulsifier: Used in polymeric coatings for polyolefin films and paper packaging.	(42)
PEG Ethers	68439-50-9	Surfactant & Antistatic Agent: Used in plastic packaging to prevent static buildup and improve surface properties.	(43)

Table S5. Precision of direct ELISA for quantification of maternal and newborn anti-PEG antibodies

	Intra-assay precision (CV%)[a]		Inter-assay precision (CV%)[b]
	Anti-PEG antibody standards	Samples	
**Pregnant women**			
Anti-PEG IgG1	3.532 ± 1.694	3.640 ± 4.892	4.353 ± 1.870
Anti-PEG IgG2	3.124 ± 2.404	6.018 ± 5.403	4.396 ± 1.393
Anti-PEG IgG3	4.247 ± 3.403	3.982 ± 4.143	7.825 ± 4.511
Anti-PEG IgG4	4.776 ± 2.557	4.477 ± 3.853	5.120 ± 2.263
Anti-PEG IgM	1.656 ± 0.825	2.864 ± 2.450	4.596 ± 2.043
Anti-PEG IgE	4.150 ± 2.756	2.346 ± 2.476	5.236 ± 2.265
**Newborns**			
Anti-PEG IgG1	3.964 ± 3.573	3.553 ± 4.379	12.725 ± 11.025
Anti-PEG IgG2	2.566 ± 1.855	4.245 ± 3.635	9.102 ± 6.077
Anti-PEG IgG3	4.004 ± 1.728	3.403 ± 3.213	9.963 ± 5.808
Anti-PEG IgG4	4.494 ± 4.509	3.220 ± 2.679	4.778 ± 3.877
Anti-PEG IgM	2.814 ± 1.138	3.163 ± 3.054	3.748 ± 0.592
Anti-PEG IgE	4.390 ± 3.514	1.351 ± 1.568	5.599 ± 2.640

[a]Intra-assay precision was evaluated by calculating the coefficient of variation (CV% = (Standard deviation/Mean) × 100%) for all anti-PEG antibody standards and samples in two independent batches of direct ELISA (see eMethods 5 for acceptance criteria).

[b]Inter-assay precision was determined by calculating the coefficient of variation for serially diluted anti-PEG antibody standards among two independent batches of direct ELISA (see eMethods 5 for acceptance criteria). Data were presented as "mean ± standard deviation", with n = 14 for intra-assay precision (CV%) of anti-PEG antibody standards in all direct ELISA, n = 256 for intra-assay precision (CV%) of serum samples in all direct ELISA, n = 7 for inter-assay precision (CV%) in all direct ELISA.

Figure S1. Detection of anti-PEG IgG1 in maternal serum samples by direct ELISA (Batch 1)

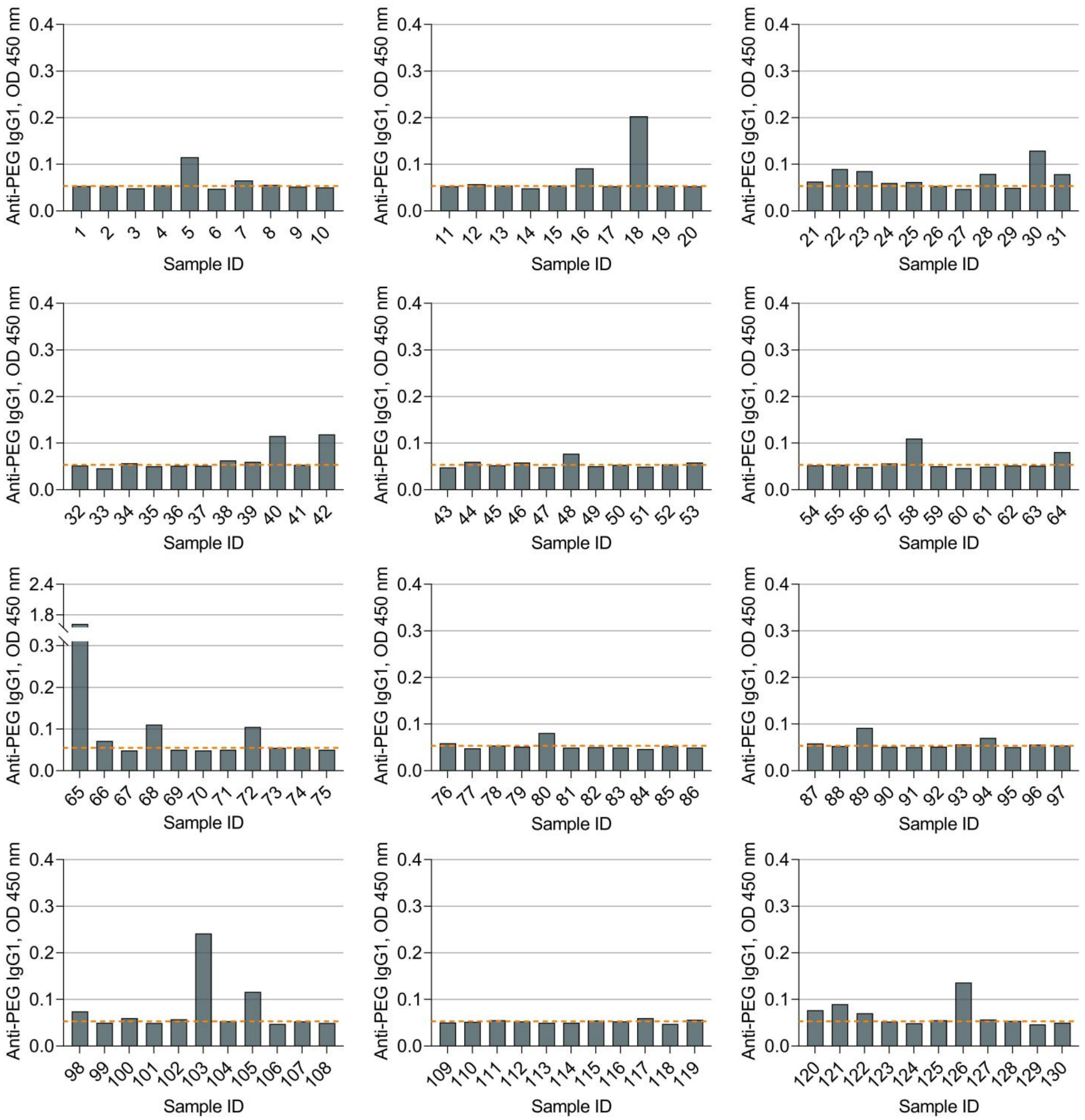

Dotted lines indicate the detection cutoff of anti-PEG IgG1 (see eMethods 3 for the establishment of detection cutoff of anti-PEG IgG1).

Figure S2. Detection of anti-PEG IgG1 in maternal serum samples by direct ELISA (Batch 2)

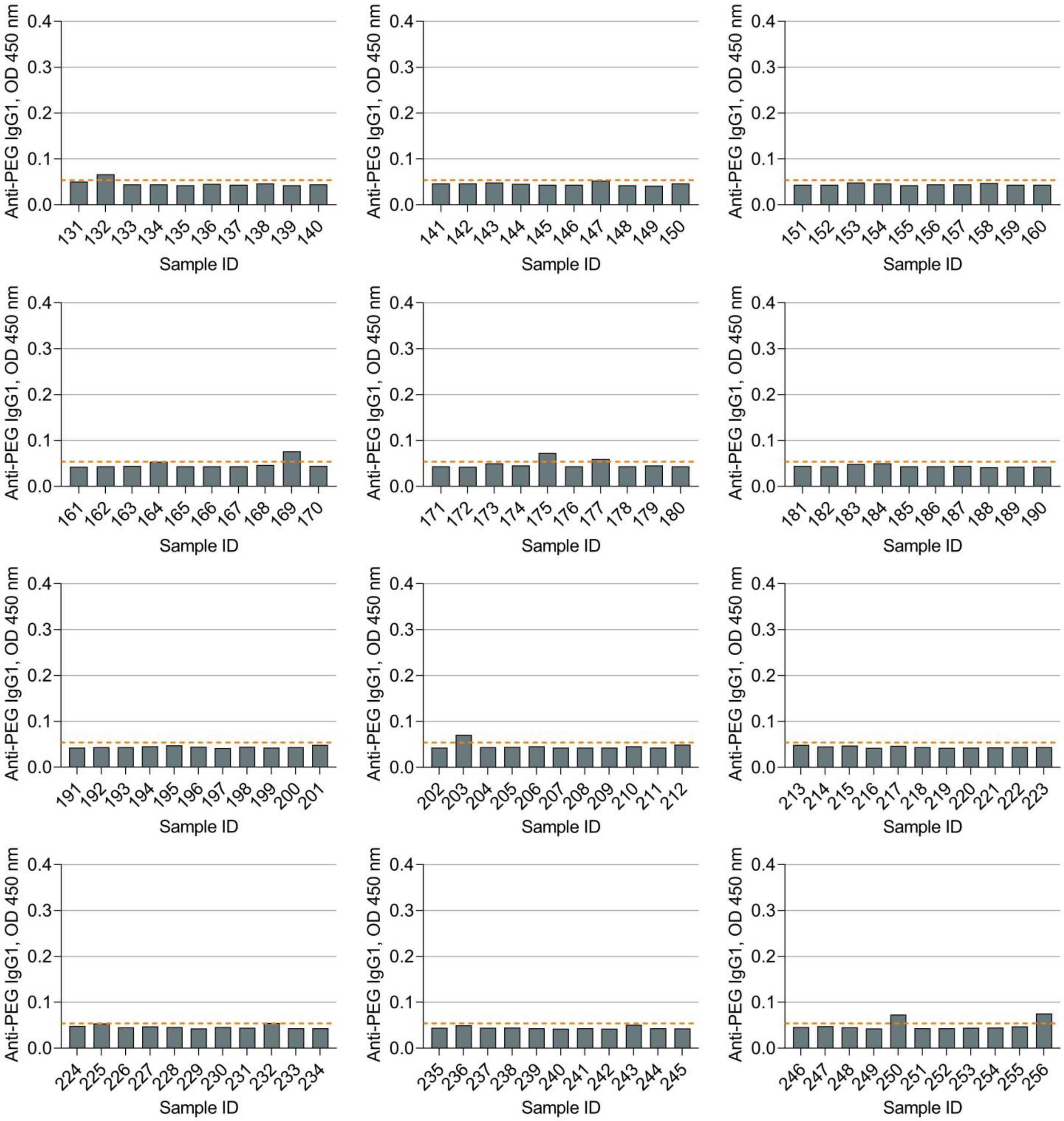

Dotted lines indicate the detection cutoff of anti-PEG IgG1 (see eMethods 3 for the establishment of detection cutoff of anti-PEG IgG1).

Figure S3. Detection of anti-PEG IgG1 in newborn serum samples by direct ELISA (Batch 1)

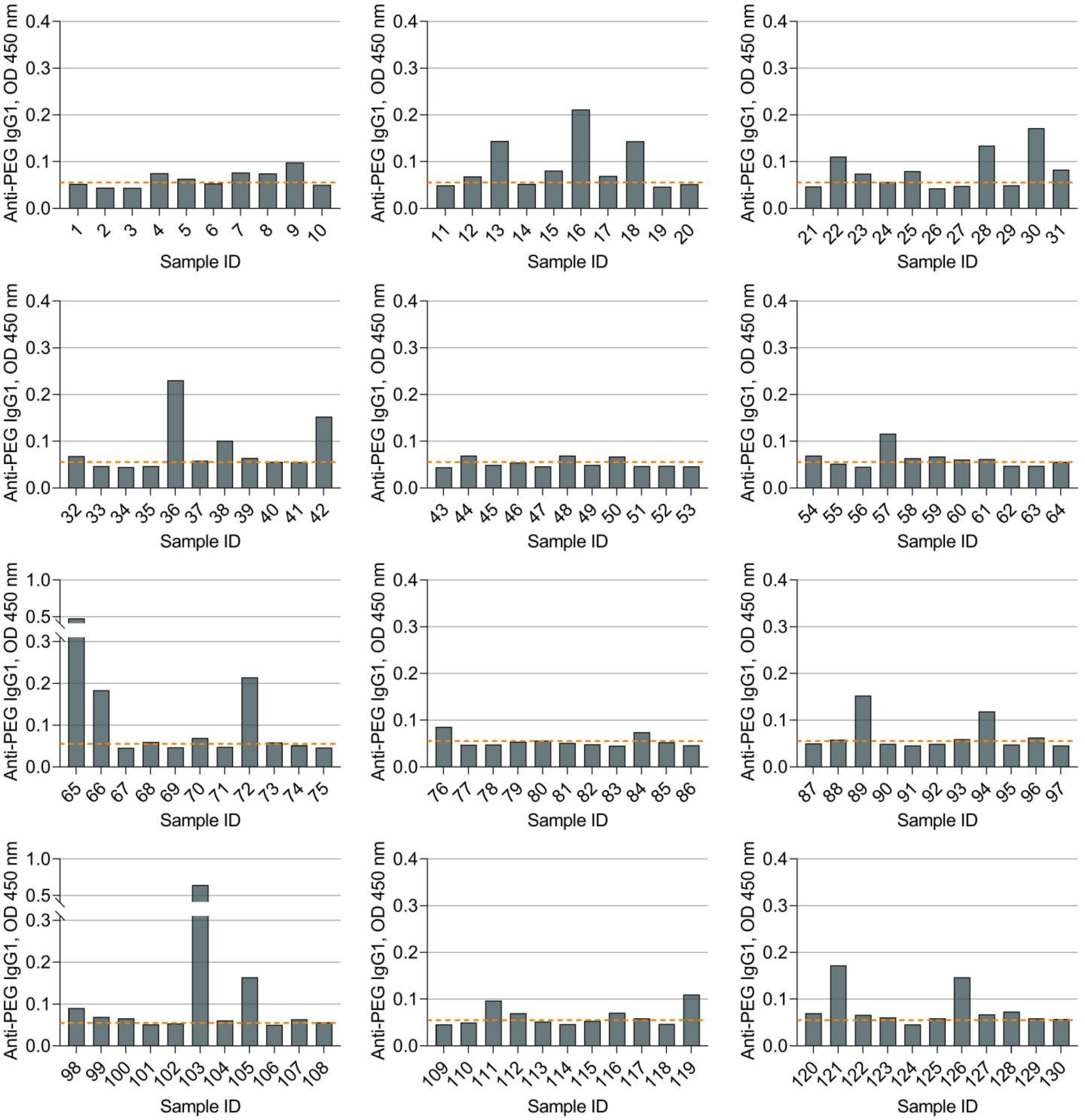

Dotted lines indicate the detection cutoff of anti-PEG IgG1 (see eMethods 3 for the establishment of detection cutoff of anti-PEG IgG1).

Figure S4. Detection of anti-PEG IgG1 in newborn serum samples by direct ELISA (Batch 2)

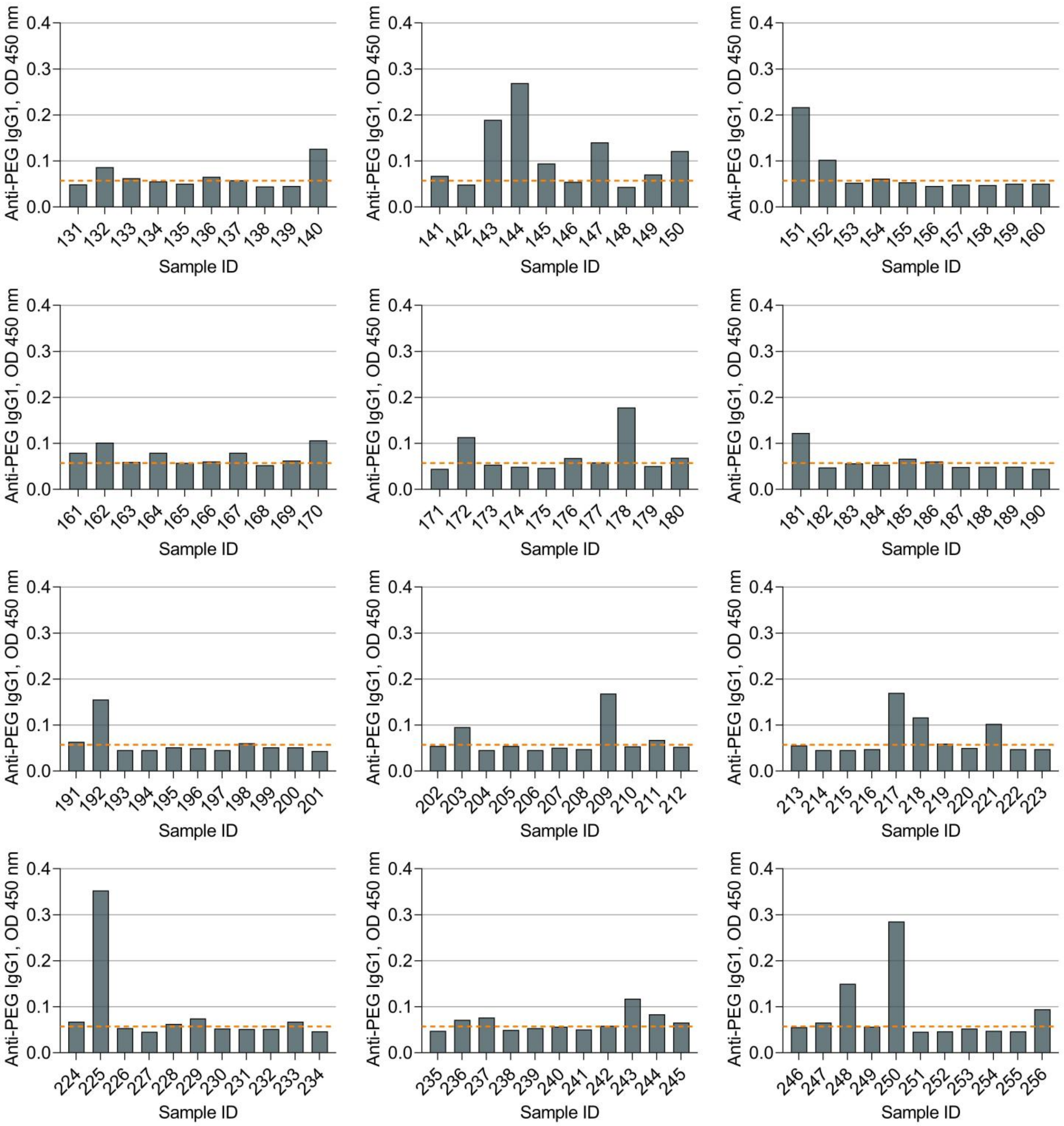

Dotted lines indicate the detection cutoff of anti-PEG IgG1 (see eMethods 3 for the establishment of detection cutoff of anti-PEG IgG1).

Figure S5. Detection of anti-PEG IgG2 in maternal serum samples by direct ELISA (Batch 1)

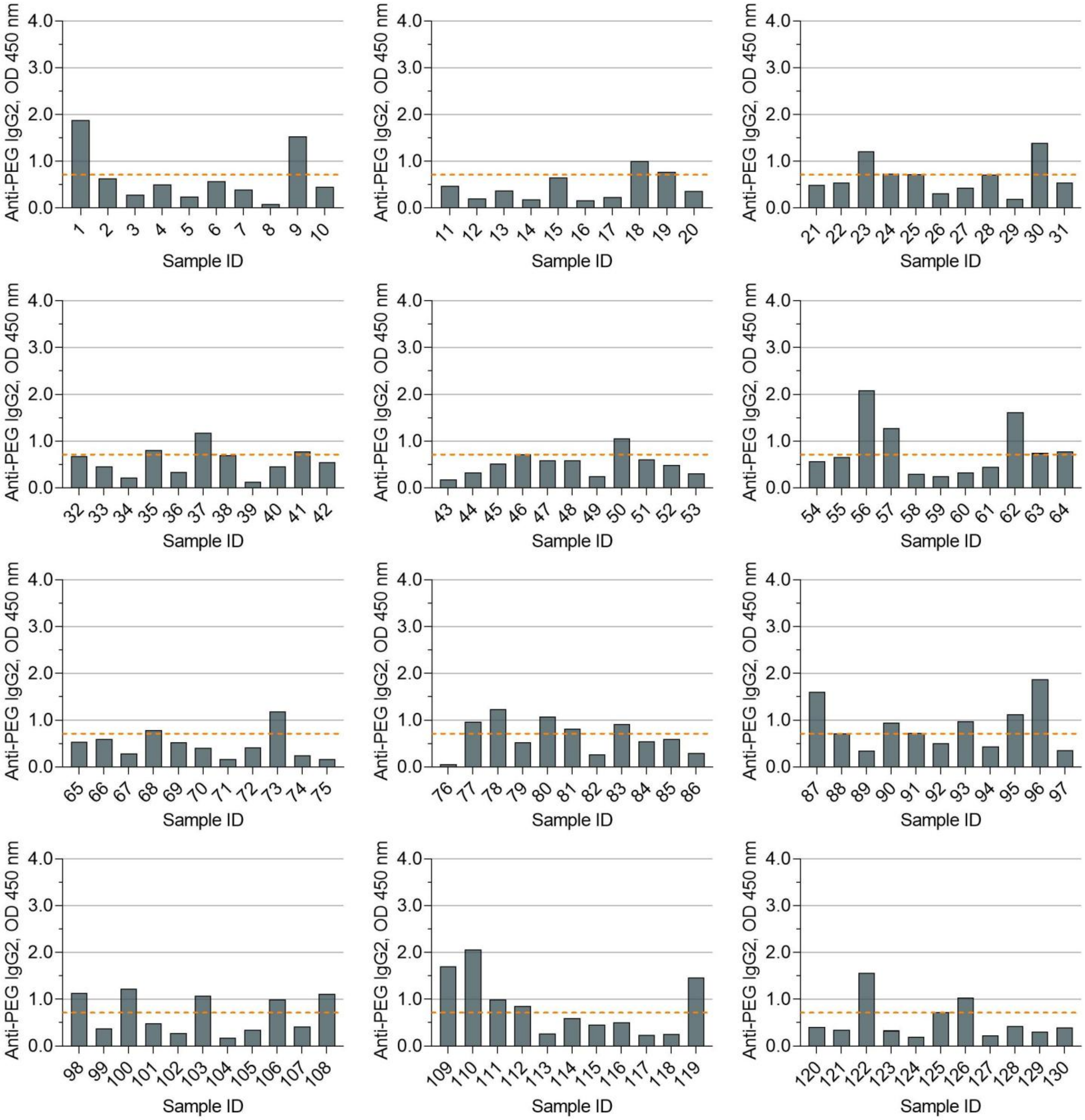

Dotted lines indicate the detection cutoff of anti-PEG IgG2 (see eMethods 3 for the establishment of detection cutoff of anti-PEG IgG2).

Figure S6. Detection of anti-PEG IgG2 in maternal serum samples by direct ELISA (Batch 2)

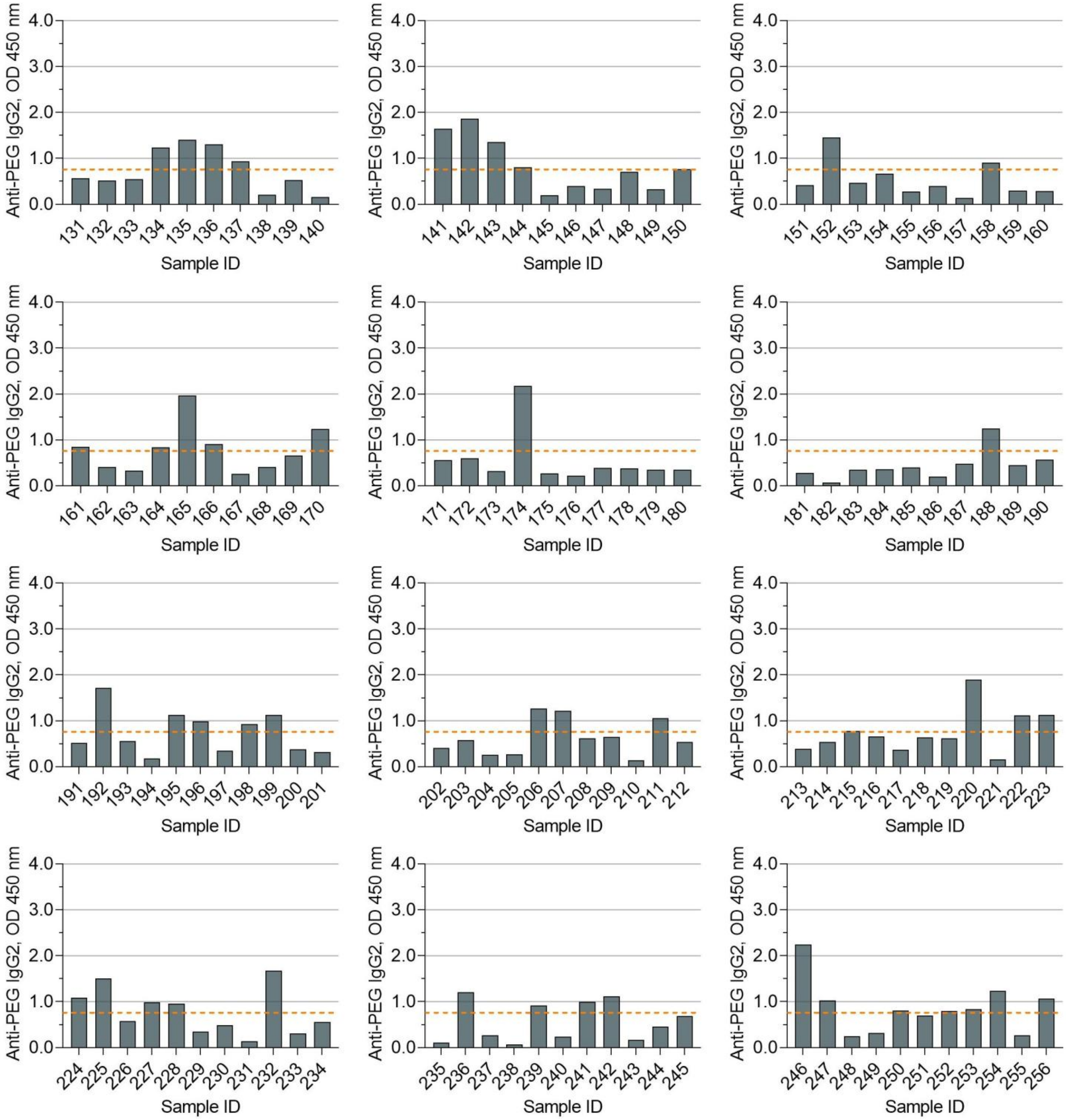

Dotted lines indicate the detection cutoff of anti-PEG IgG2 (see eMethods 3 for the establishment of detection cutoff of anti-PEG IgG2).

Figure S7. Detection of anti-PEG IgG2 in newborn serum samples by direct ELISA (Batch 1)

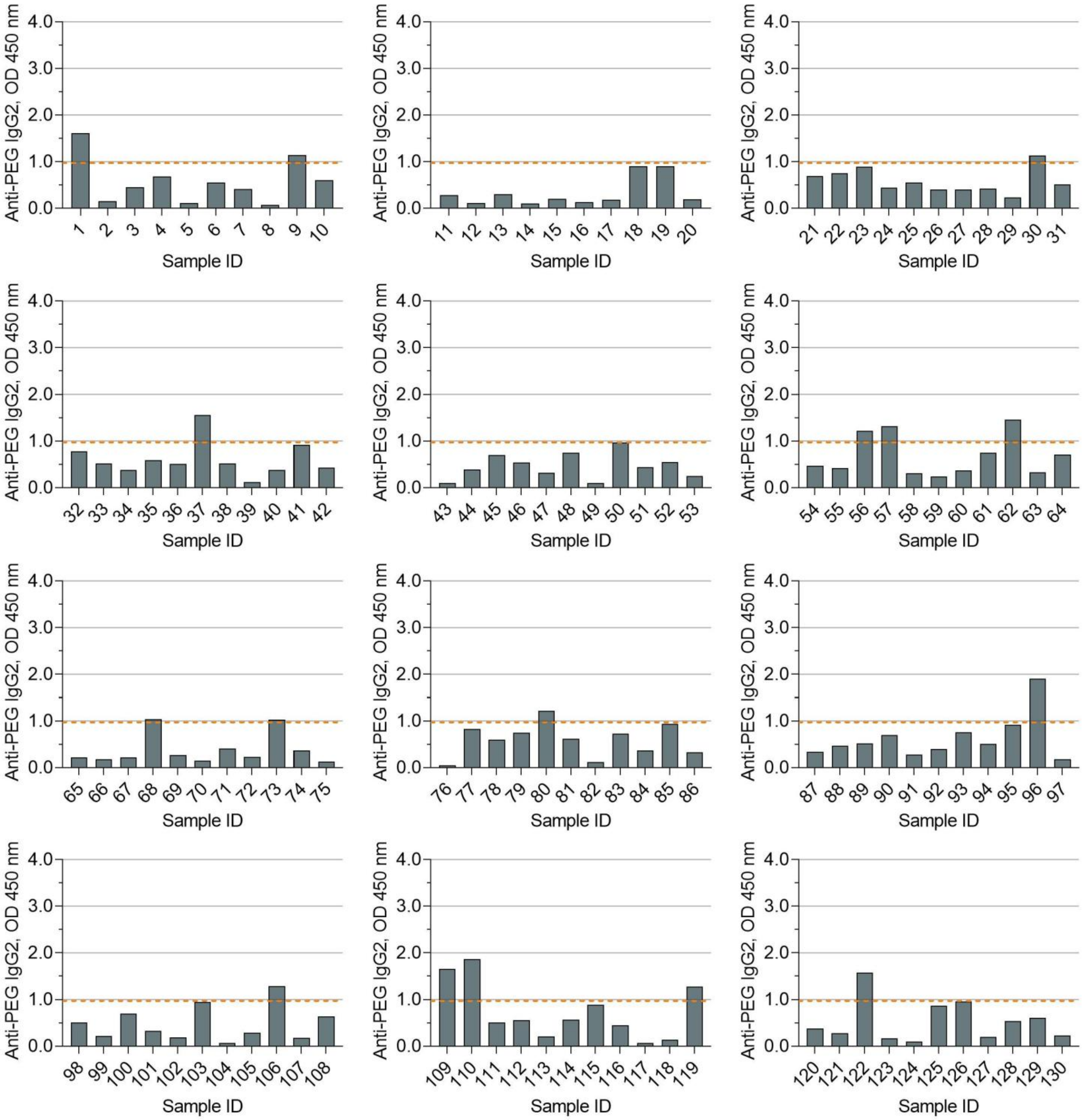

Dotted lines indicate the detection cutoff of anti-PEG IgG2 (see eMethods 3 for the establishment of detection cutoff of anti-PEG IgG2).

Figure S8. Detection of anti-PEG IgG2 in newborn serum samples by direct ELISA (Batch 2)

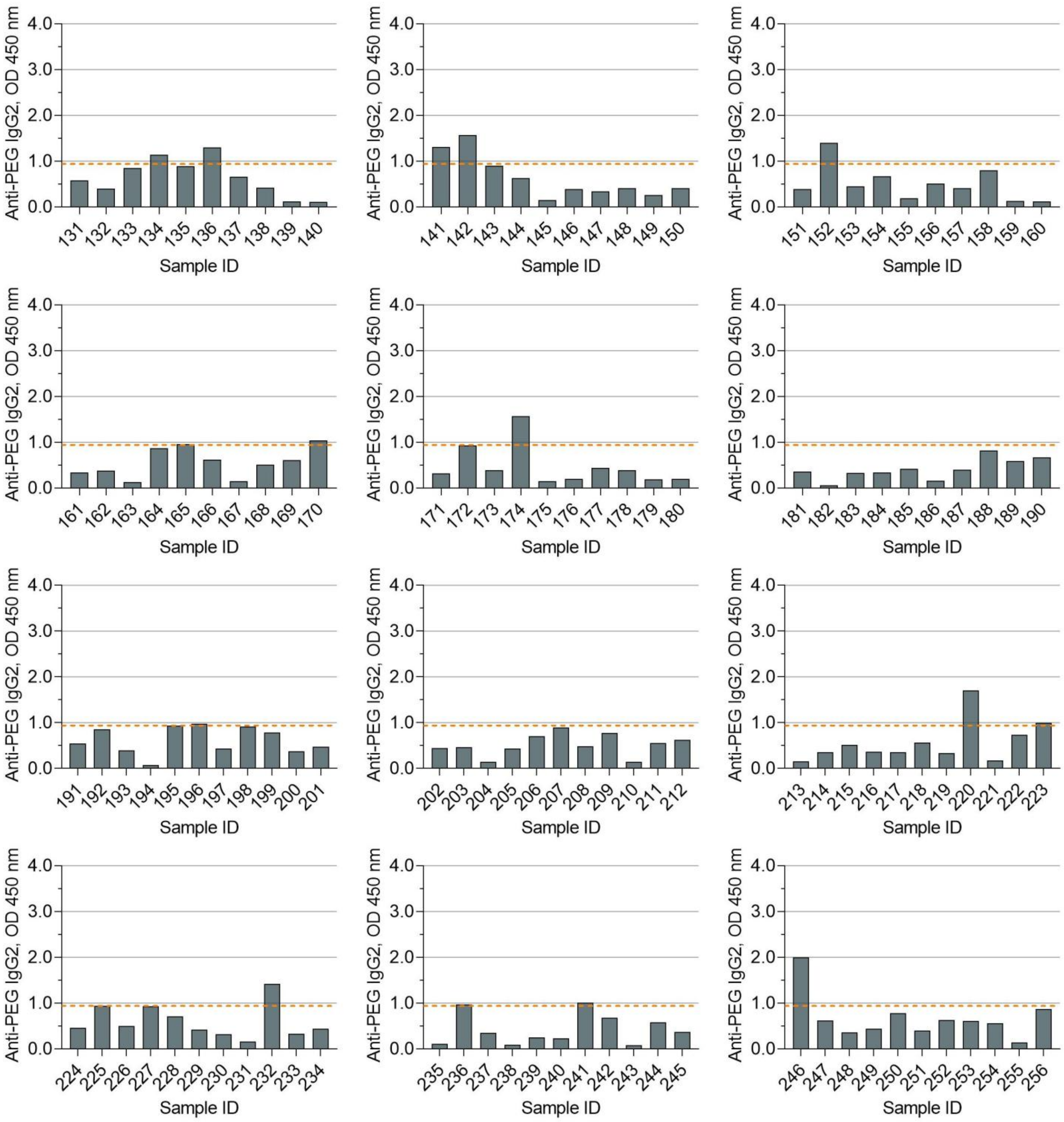

Dotted lines indicate the detection cutoff of anti-PEG IgG2 (see eMethods 3 for the establishment of detection cutoff of anti-PEG IgG2).

Figure S9. Detection of anti-PEG IgG3 in maternal serum samples by direct ELISA (Batch 1)

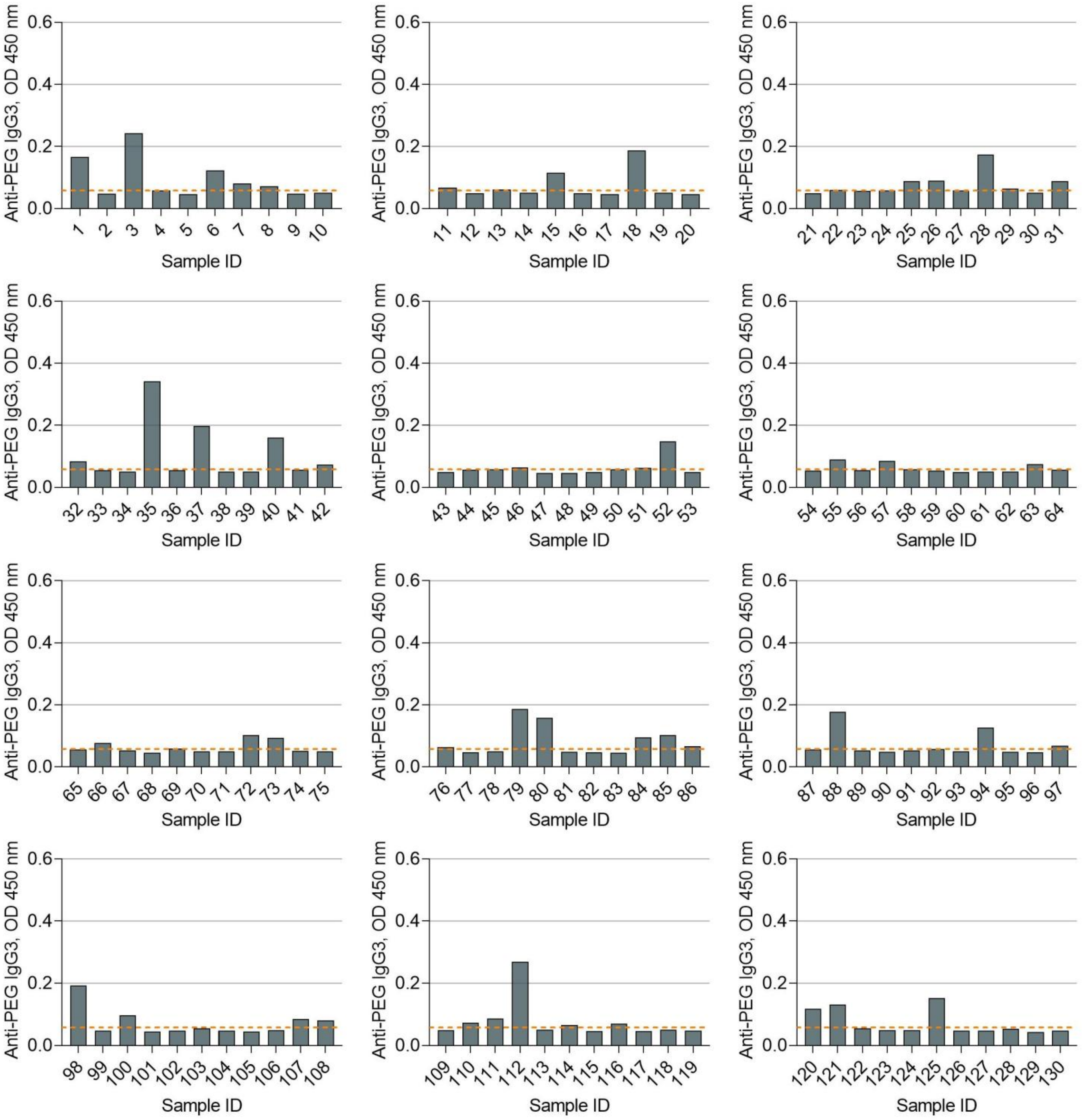

Dotted lines indicate the detection cutoff of anti-PEG IgG3 (see eMethods 3 for the establishment of detection cutoff of anti-PEG IgG3).

Figure S10. Detection of anti-PEG IgG3 in maternal serum samples by direct ELISA (Batch 2)

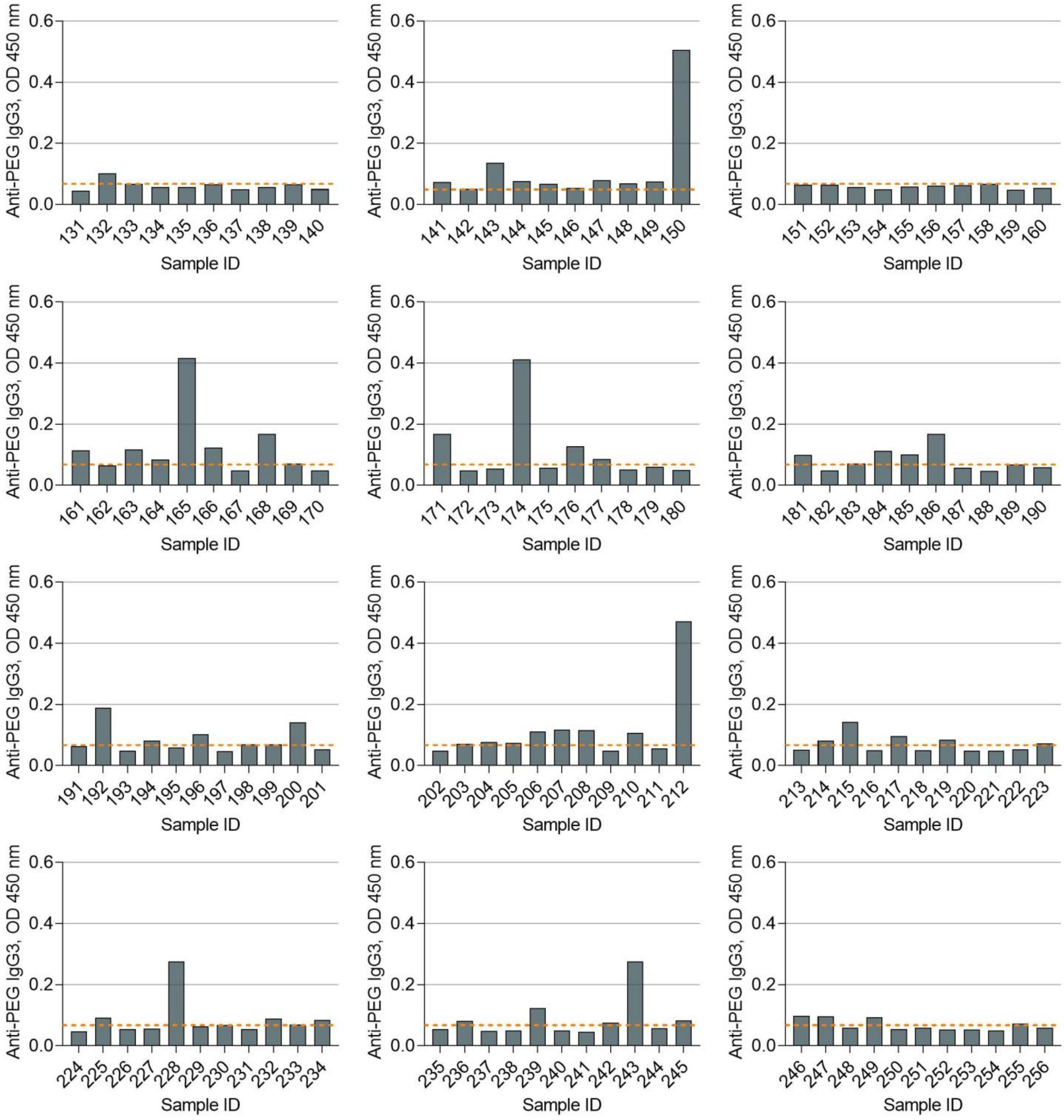

Dotted lines indicate the detection cutoff of anti-PEG IgG3 (see eMethods 3 for the establishment of detection cutoff of anti-PEG IgG3).

Figure S11. Detection of anti-PEG IgG3 in newborn serum samples by direct ELISA (Batch 1)

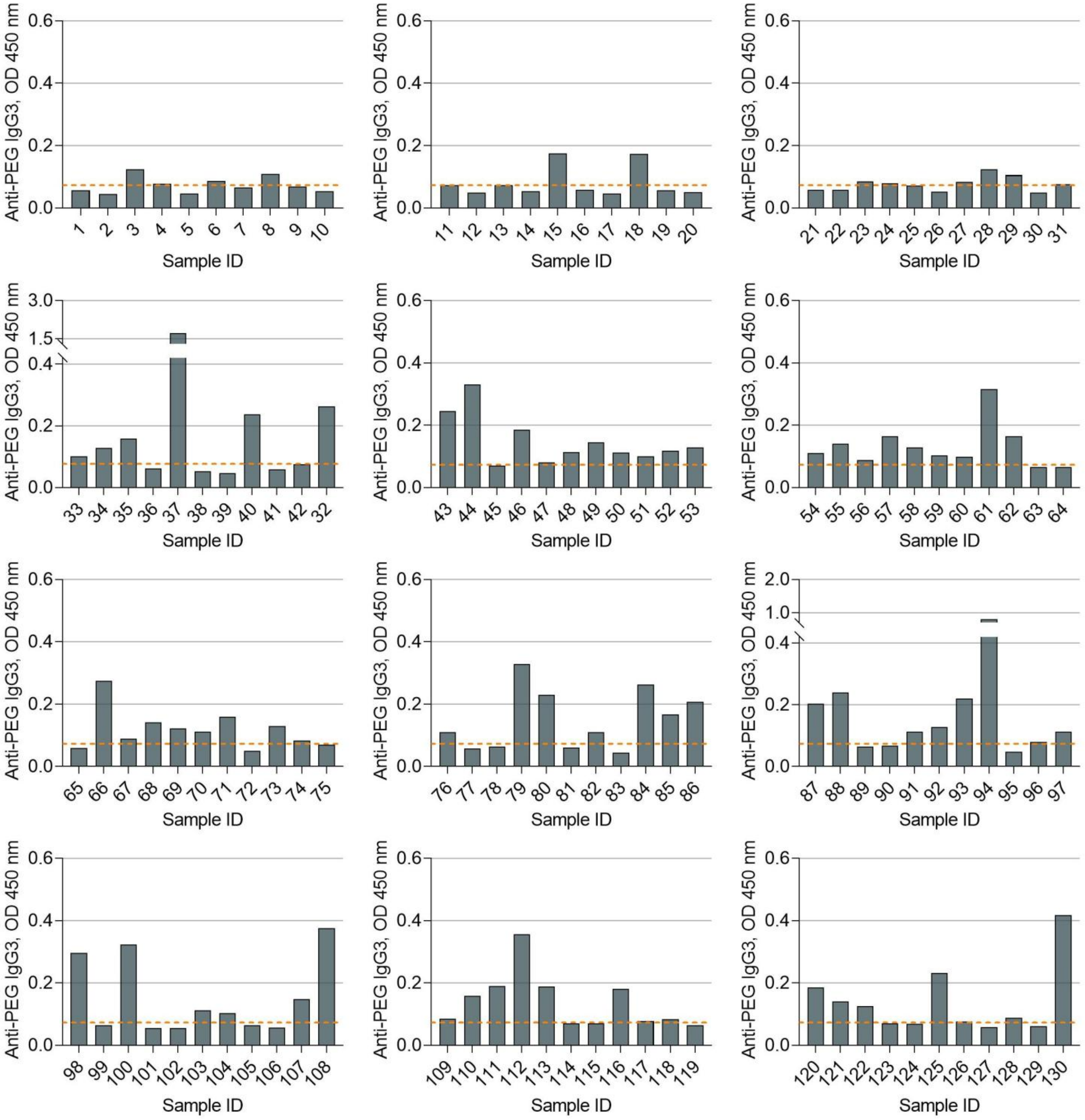

Dotted lines indicate the detection cutoff of anti-PEG IgG3 (see eMethods 3 for the establishment of detection cutoff of anti-PEG IgG3).

Figure S12. Detection of anti-PEG IgG3 in newborn serum samples by direct ELISA (Batch 2)

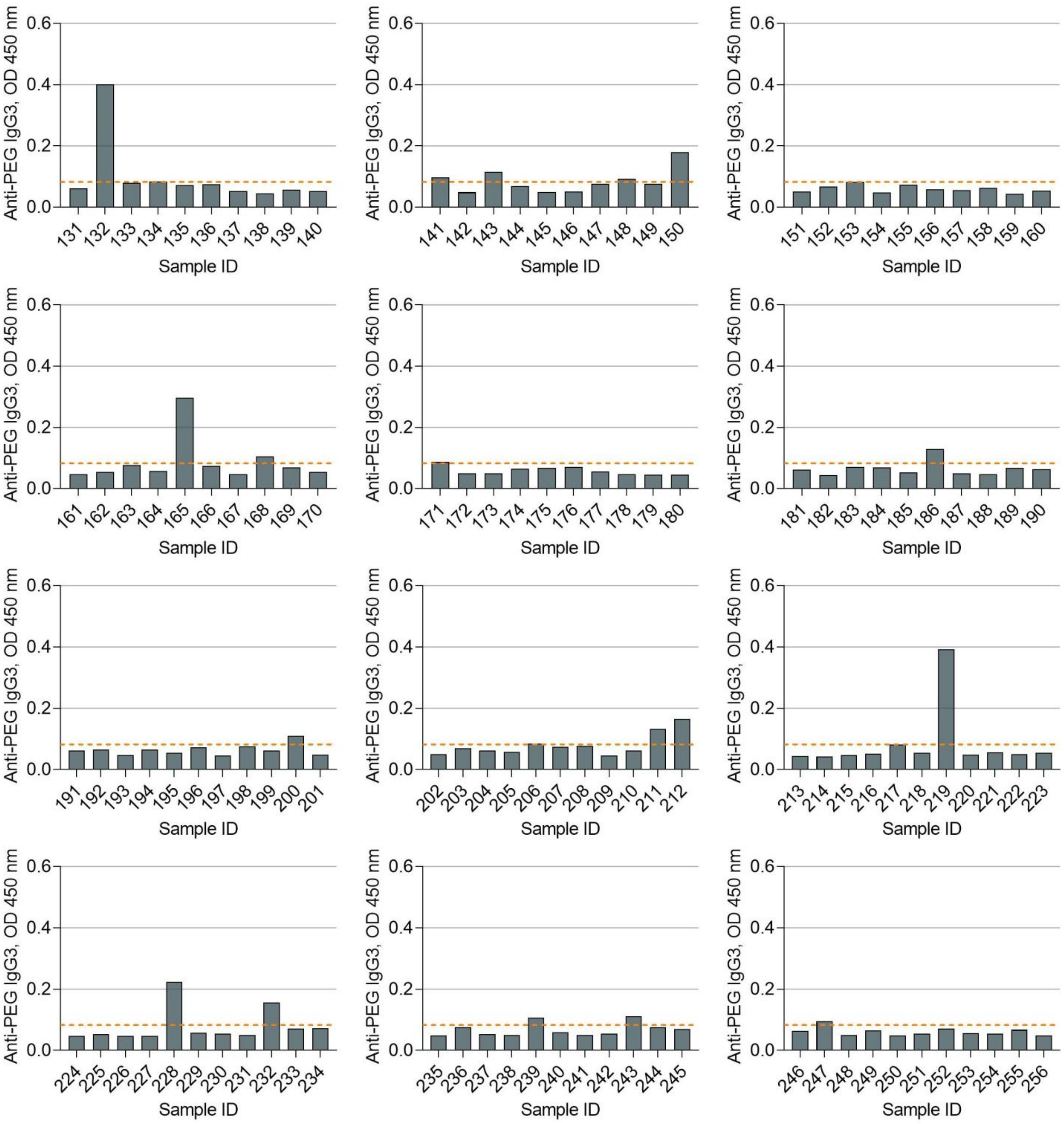

Dotted lines indicate the detection cutoff of anti-PEG IgG3 (see eMethods 3 for the establishment of detection cutoff of anti-PEG IgG3).

Figure S13. Detection of anti-PEG IgG4 in maternal serum samples by direct ELISA (Batch 1)

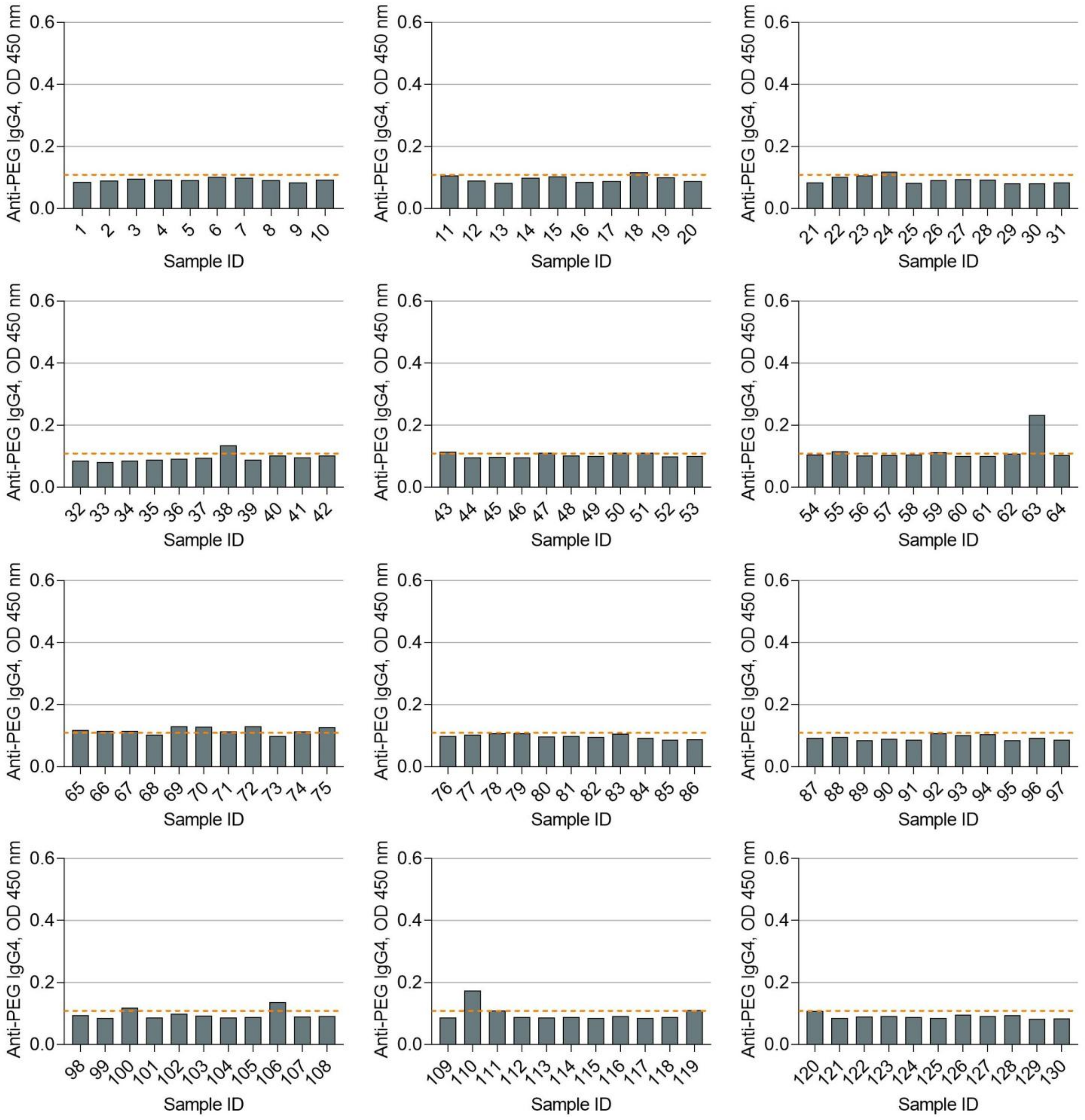

Dotted lines indicate the detection cutoff of anti-PEG IgG4 (see eMethods 3 for the establishment of detection cutoff of anti-PEG IgG4).

Figure S14. Detection of anti-PEG IgG4 in maternal serum samples by direct ELISA (Batch 2)

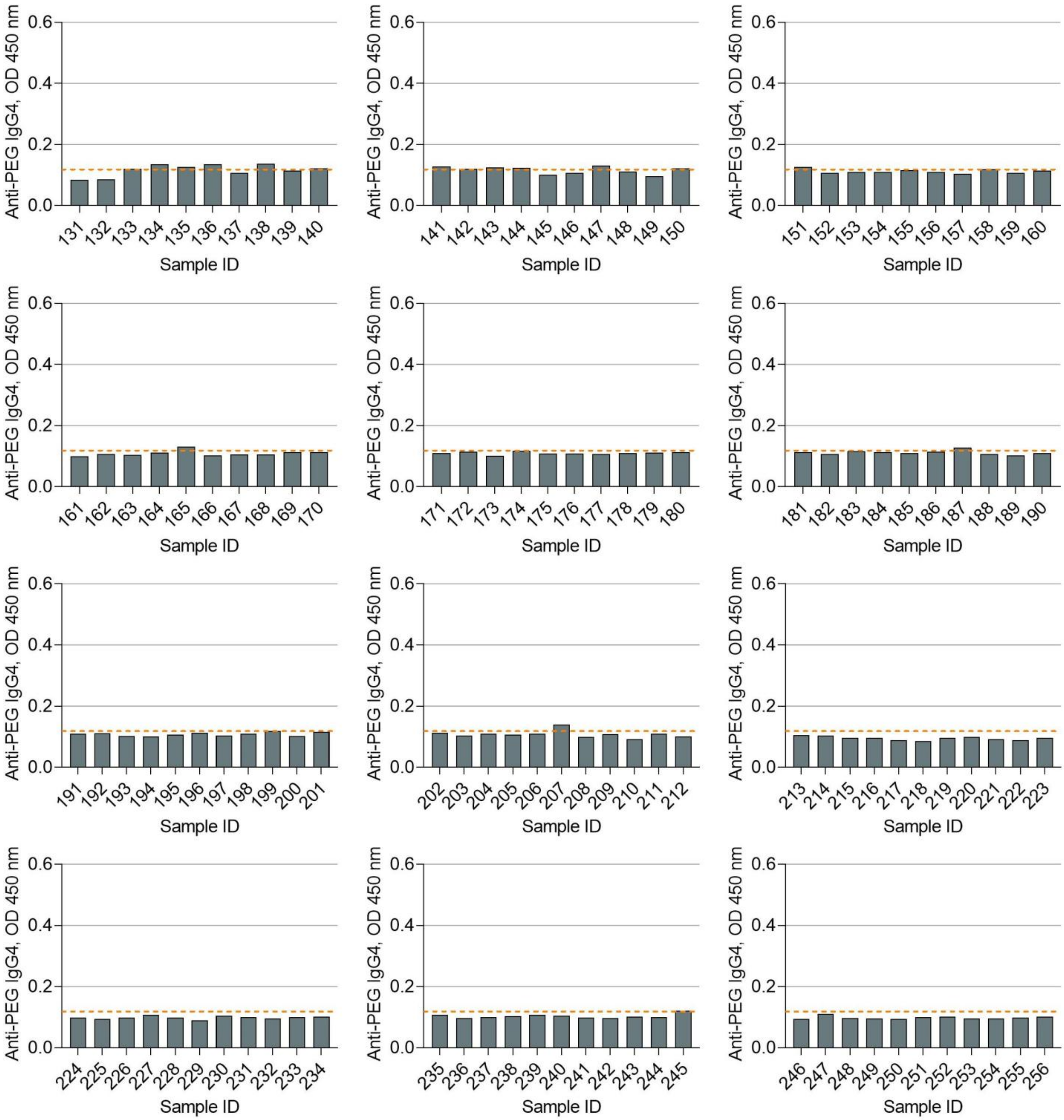

Dotted lines indicate the detection cutoff of anti-PEG IgG4 (see eMethods 3 for the establishment of detection cutoff of anti-PEG IgG4).

Figure S15. Detection of anti-PEG IgG4 in newborn serum samples by direct ELISA (Batch 1)

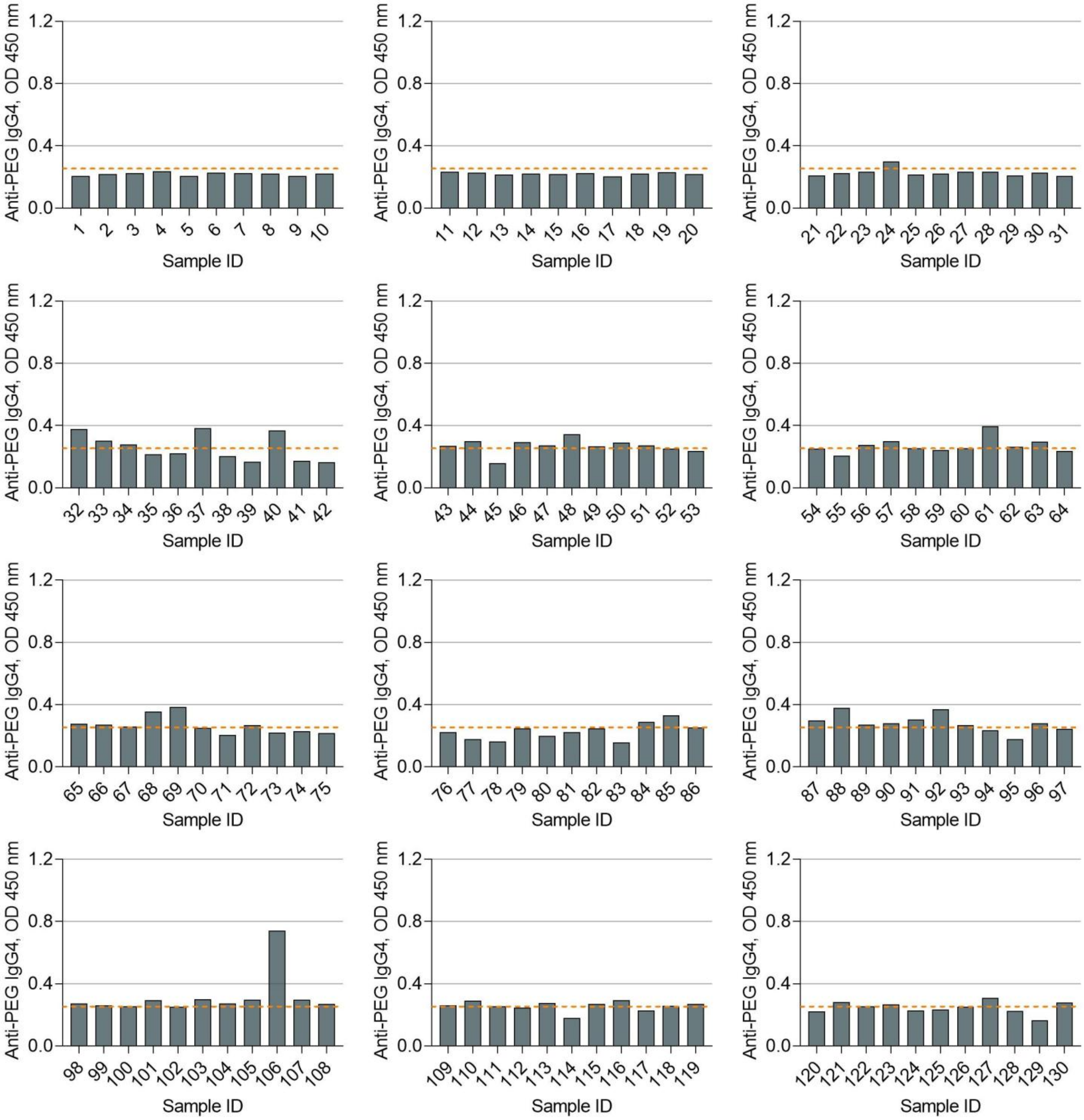

Dotted lines indicate the detection cutoff of anti-PEG IgG4 (see eMethods 3 for the establishment of detection cutoff of anti-PEG IgG4).

Figure S16. Detection of anti-PEG IgG4 in newborn serum samples by direct ELISA (Batch 2)

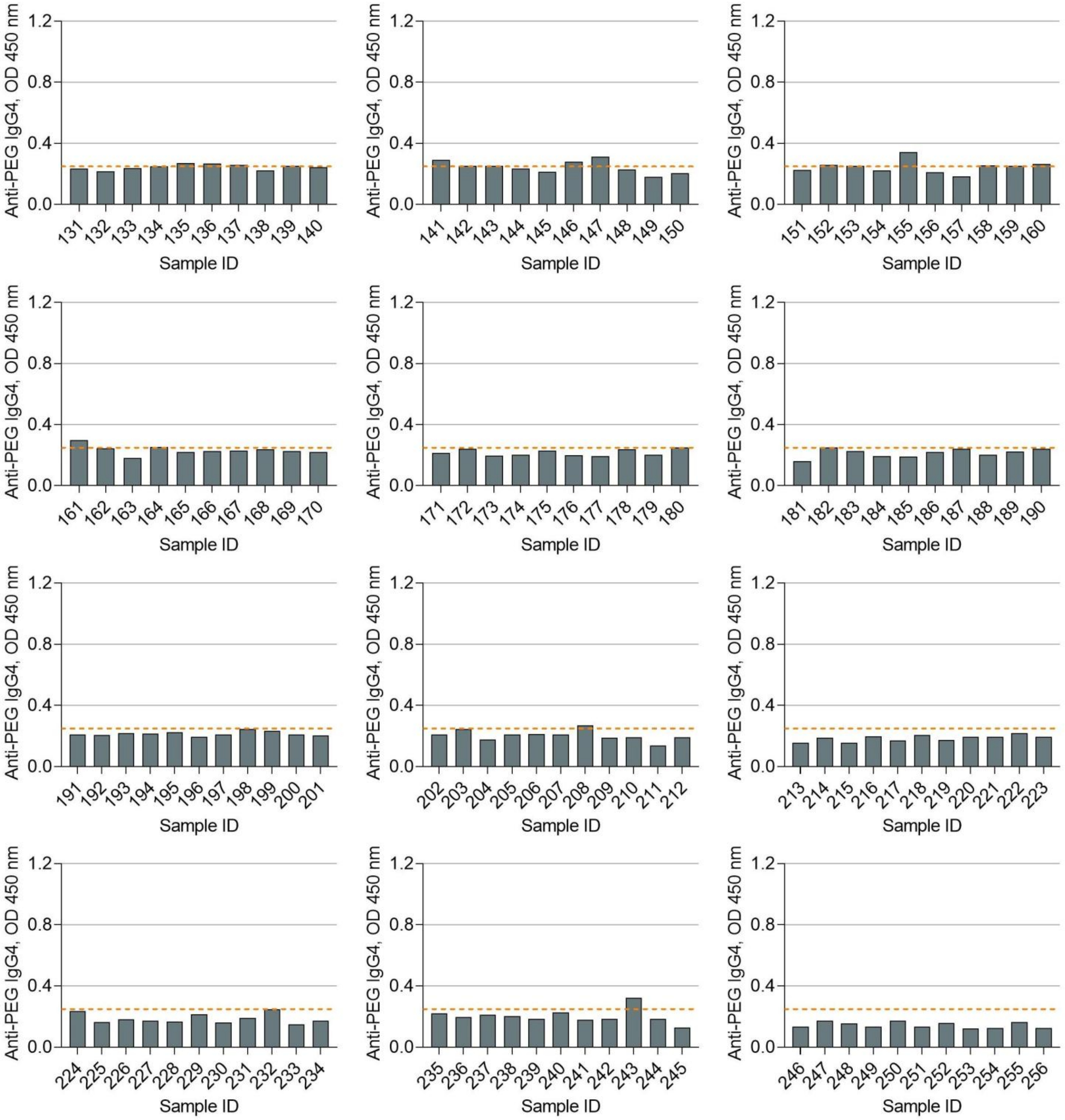

Dotted lines indicate the detection cutoff of anti-PEG IgG4 (see eMethods 3 for the establishment of detection cutoff of anti-PEG IgG4).

Figure S17. Detection of anti-PEG IgM in maternal serum samples by direct ELISA (Batch 1)

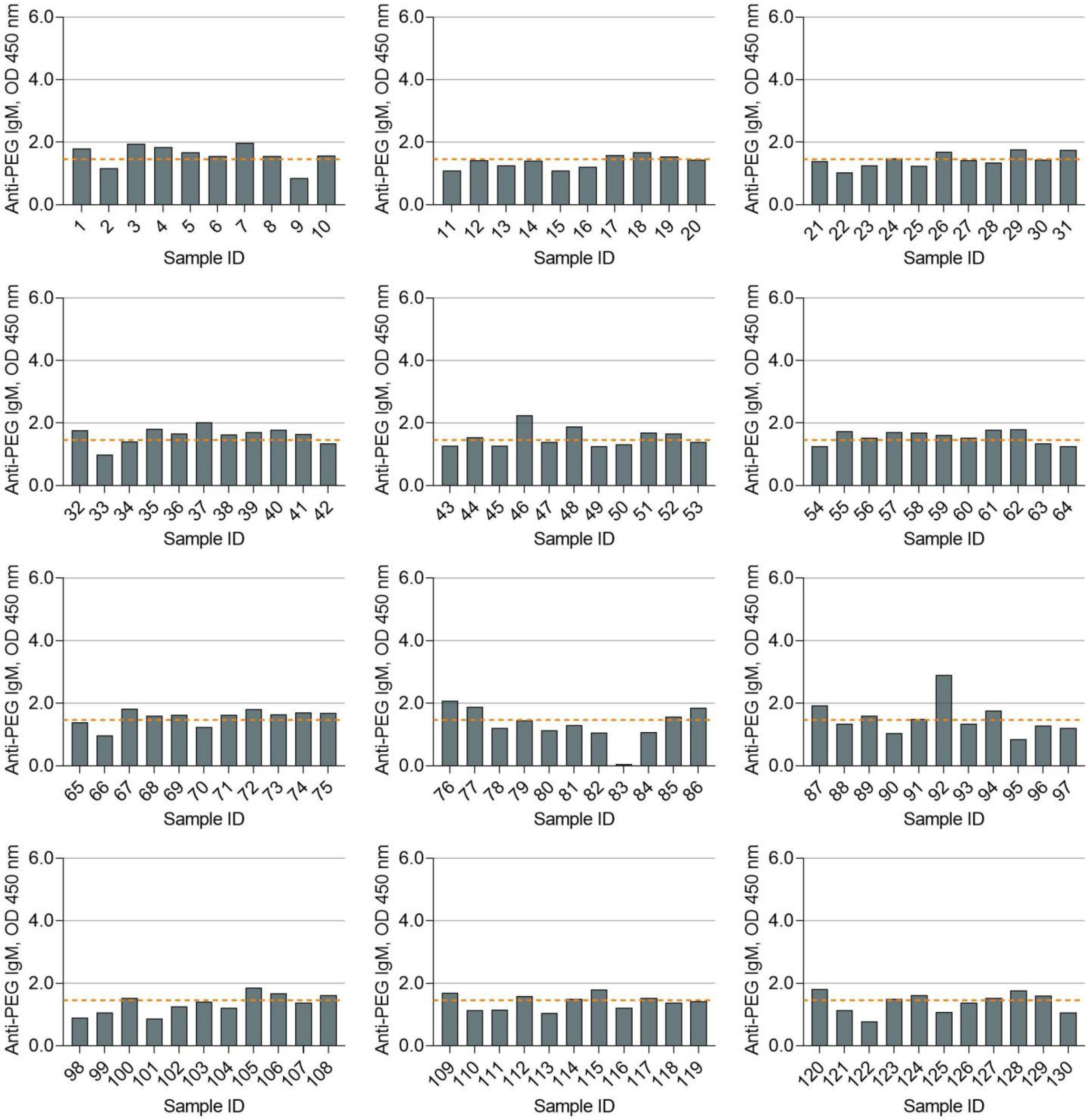

Dotted lines indicate the detection cutoff of anti-PEG IgM (see eMethods 3 for the establishment of detection cutoff of anti-PEG IgM).

Figure S18. Detection of anti-PEG IgM in maternal serum samples by direct ELISA (Batch 2)

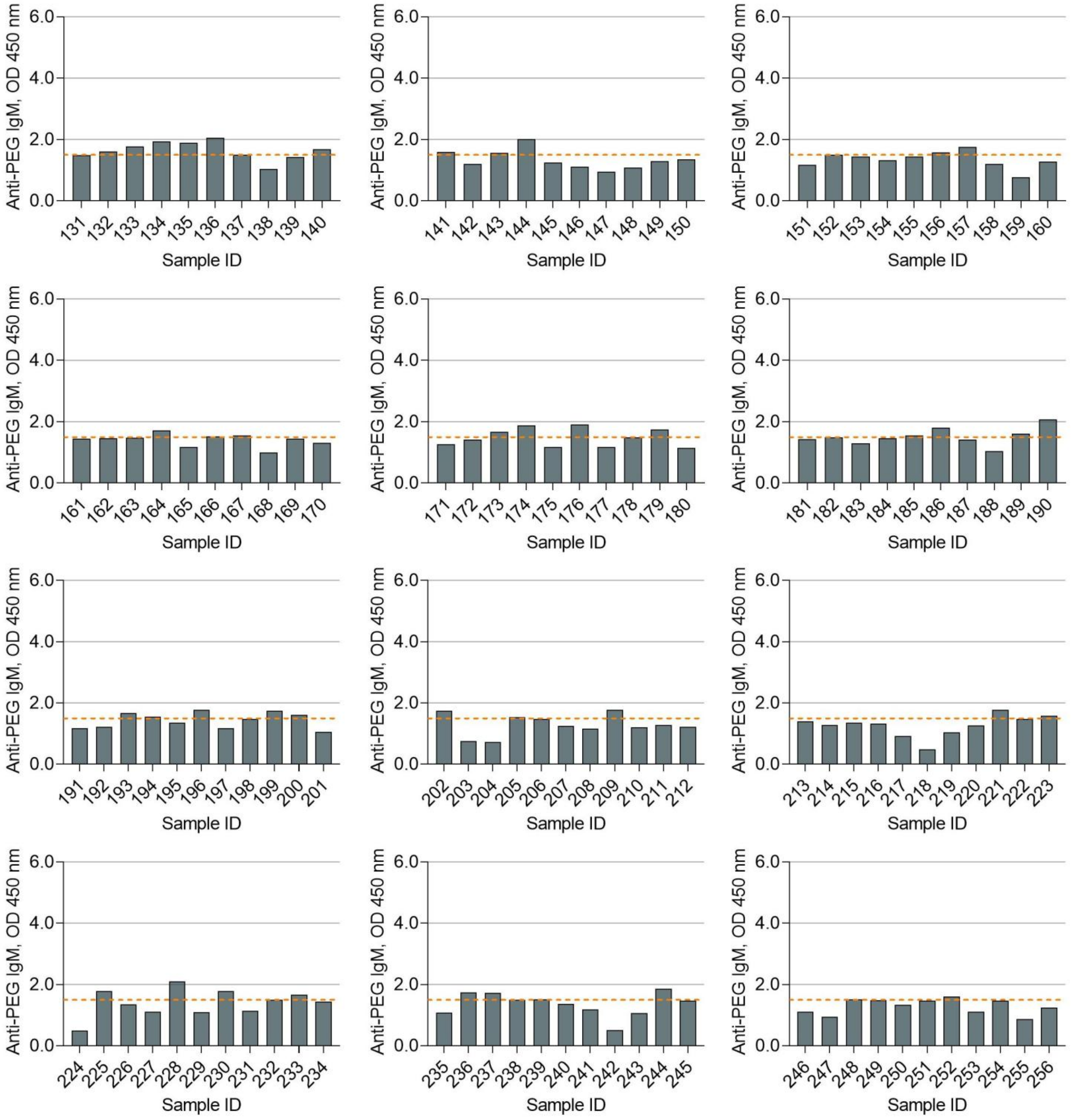

Dotted lines indicate the detection cutoff of anti-PEG IgM (see eMethods 3 for the establishment of detection cutoff of anti-PEG IgM).

Figure S19. Detection of anti-PEG IgM in newborn serum samples by direct ELISA (Batch 1)

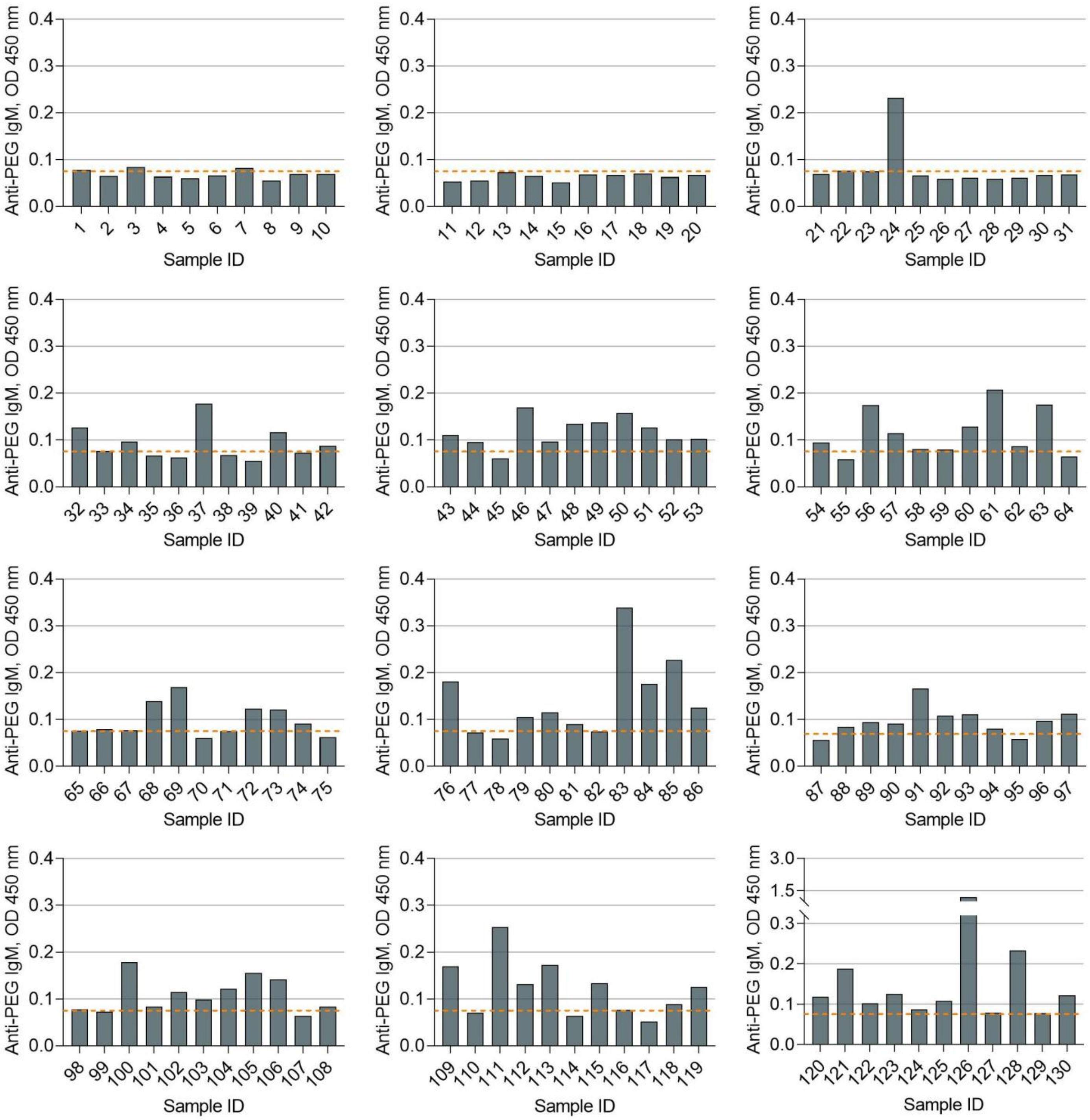

Dotted lines indicate the detection cutoff of anti-PEG IgM (see eMethods 3 for the establishment of detection cutoff of anti-PEG IgM).

Figure S20. Detection of anti-PEG IgM in newborn serum samples by direct ELISA (Batch 2)

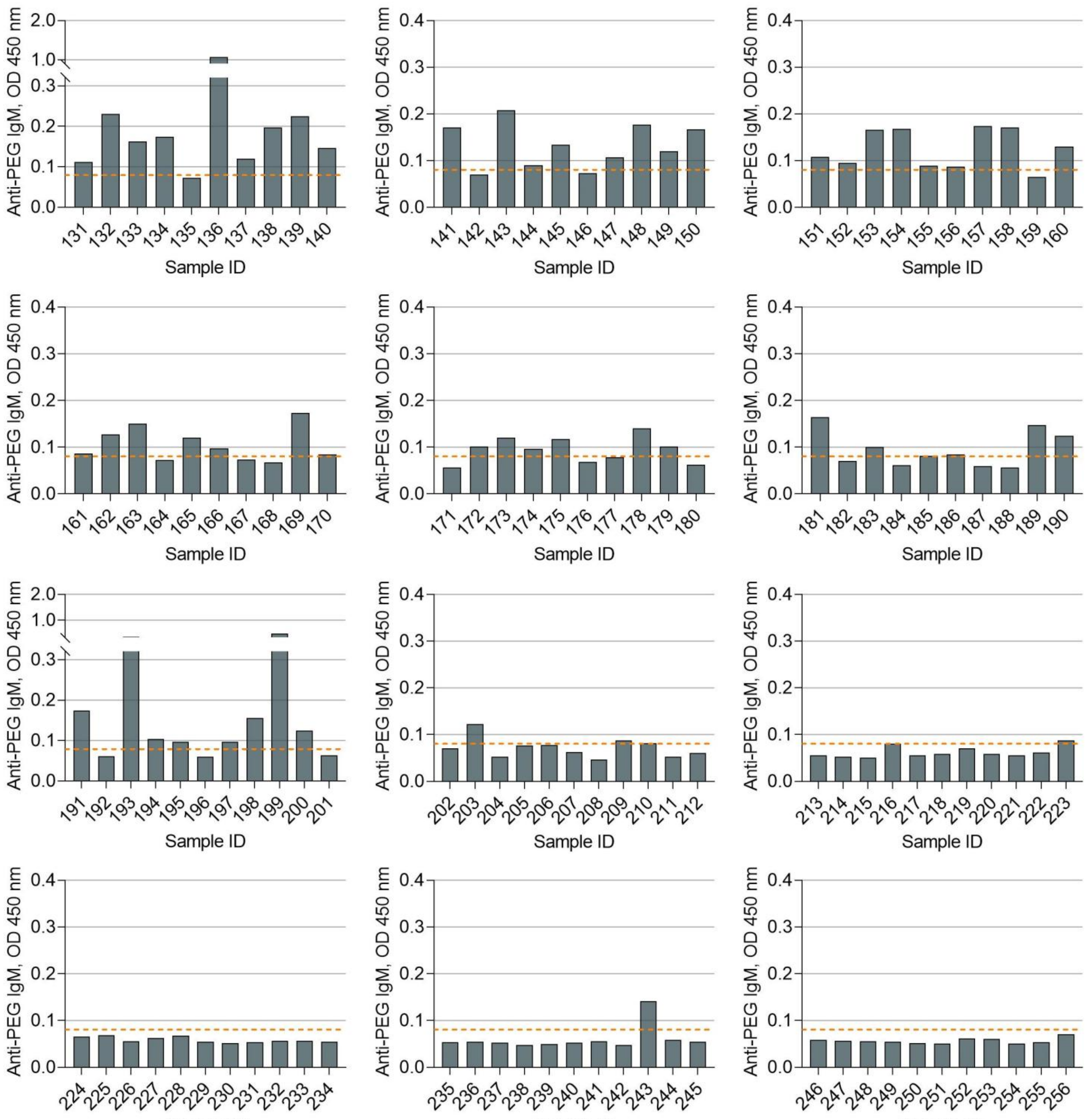

Dotted lines indicate the detection cutoff of anti-PEG IgM (see eMethods 3 for the establishment of detection cutoff of anti-PEG IgM).

Figure S21. Detection of anti-PEG IgE in maternal serum samples by direct ELISA (Batch 1)

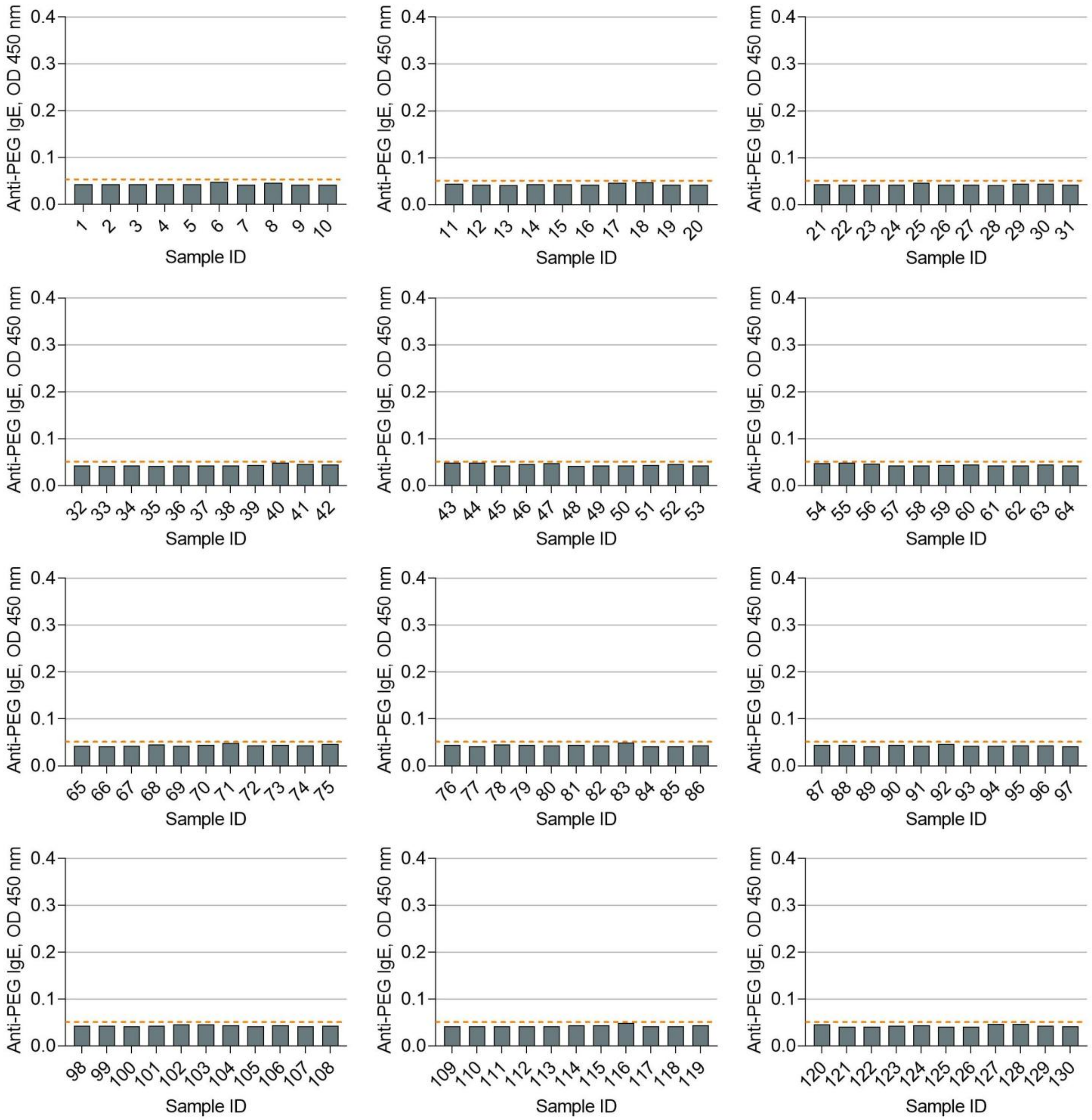

Dotted lines indicate the detection cutoff of anti-PEG IgE (see eMethods 3 for the establishment of detection cutoff of anti-PEG IgE).

Figure S22. Detection of anti-PEG IgE in maternal serum samples by direct ELISA (Batch 2)

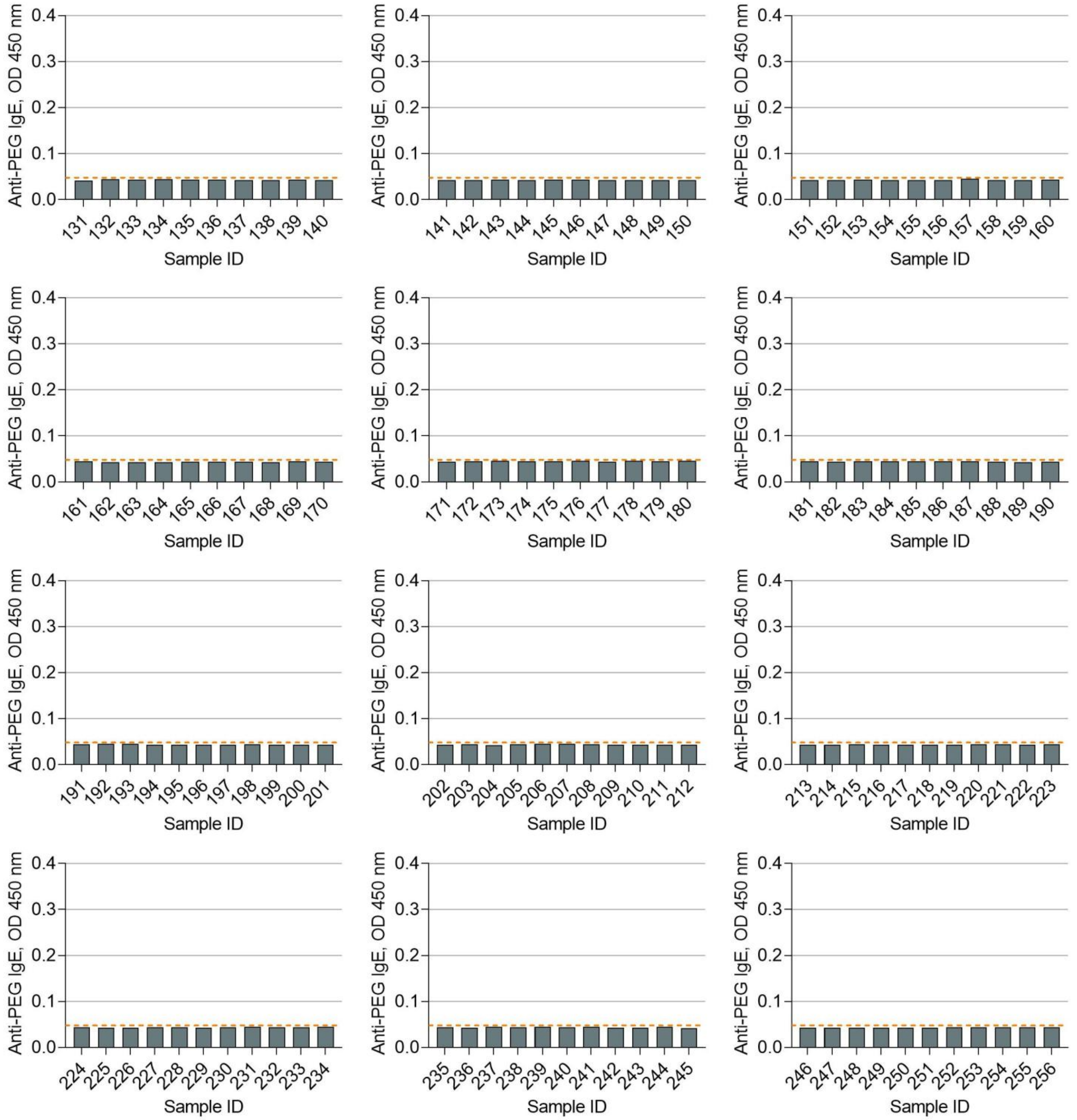

Dotted lines indicate the detection cutoff of anti-PEG IgE (see eMethods 3 for the establishment of detection cutoff of anti-PEG IgE).

Figure S23. Detection of anti-PEG IgE in newborn serum samples by direct ELISA (Batch 1)

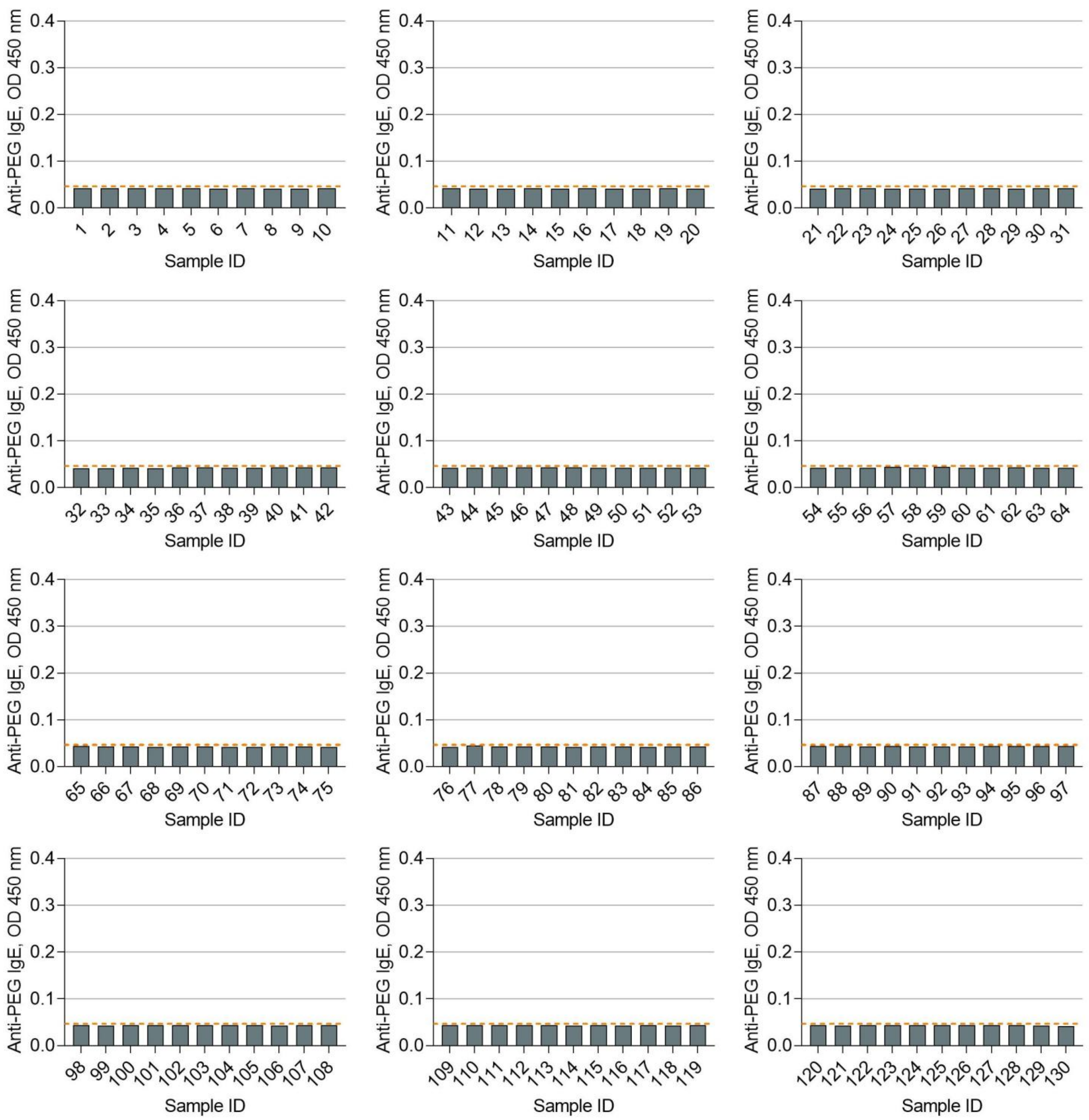

Dotted lines indicate the detection cutoff of anti-PEG IgE (see eMethods 3 for the establishment of detection cutoff of anti-PEG IgE).

Figure S24. Detection of anti-PEG IgE in newborn serum samples by direct ELISA (Batch 2)

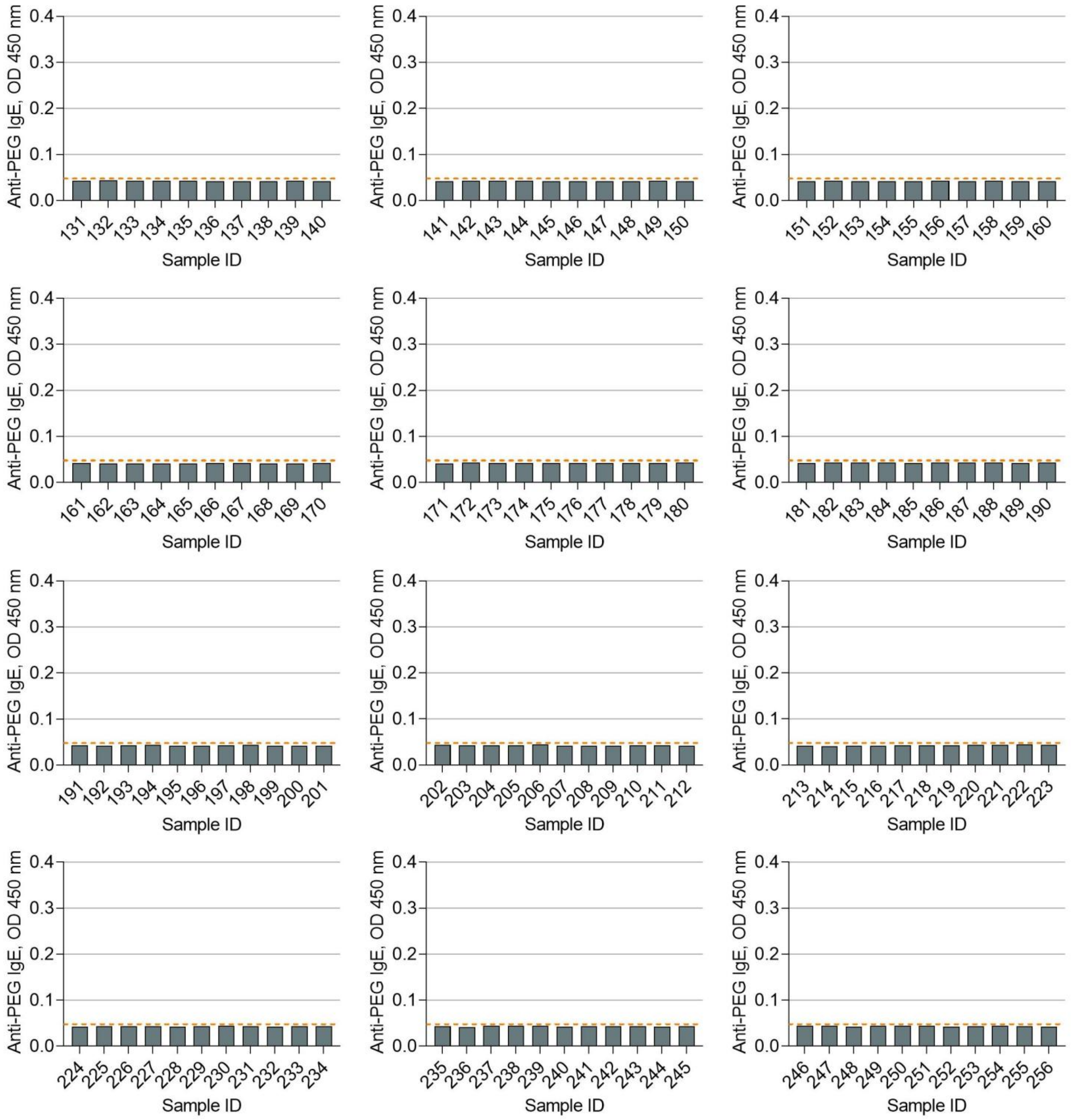

Dotted lines indicate the detection cutoff of anti-PEG IgE (see eMethods 3 for the establishment of detection cutoff of anti-PEG IgE).

Table S6. Precision of competitive ELISA for confirmation of the PEG specificity of maternal and newborn anti-PEG IgG1-4 and IgM

	Intra-assay precision (CV%)[a]			
	Anti-PEG antibody standards without LIP	Anti-PEG antibody standards with LIP	Samples without LIP	Samples with LIP
**Pregnant women**				
Anti-PEG IgG1	4.450 ± 3.277	5.298 ± 4.890	3.600 ± 3.091	3.617 ± 3.032
Anti-PEG IgG2	2.829 ± 1.566	4.006 ± 3.028	2.908 ± 2.254	2.693 ± 2.233
Anti-PEG IgG3	3.087 ± 1.620	5.827 ± 3.586	3.312 ± 3.028	2.979 ± 2.504
Anti-PEG IgG4	2.920 ± 2.128	2.267 ± 1.683	1.799 ± 1.624	1.236 ± 1.401
Anti-PEG IgM	2.872 ± 2.146	1.995 ± 1.387	2.598 ± 2.160	2.771 ± 2.387
**Newborns**				
Anti-PEG IgG1	3.268 ± 3.264	4.555 ± 5.154	3.242 ± 2.613	2.453 ± 2.449
Anti-PEG IgG2	3.571 ± 1.400	6.477 ± 2.463	3.323 ± 3.593	3.276 ± 2.490
Anti-PEG IgG3	4.058 ± 1.919	5.319 ± 3.987	2.769 ± 2.401	2.557 ± 2.425
Anti-PEG IgG4	4.220 ± 3.045	3.884 ± 2.679	2.377 ± 2.017	2.554 ± 2.060
Anti-PEG IgM	2.281 ± 0.900	3.390 ± 1.666	2.183 ± 1.908	1.962 ± 1.766

Abbreviations: LIP, PEGylated liposomal doxorubicin (competitors, see eMethods 4).

[a]Intra-assay precision was evaluated by calculating the coefficient of variation (CV% = (Standard deviation/Mean) × 100%) for all anti-PEG antibody standards without LIP, anti-PEG antibody standards with LIP, samples without LIP, and samples with LIP in one independent batch of competitive ELISA (see eMethods 5 for acceptance criteria). Data were presented as "mean ± standard deviation", with n = 9 for intra-assay precision (CV%) of anti-PEG antibody standards without LIP and with LIP in all competitive ELISA, n = 71, 118, 92, 35, 118, 74, 45, 84, 103, 138 for intra-assay precision (CV%) of samples without LIP and with LIP in competitive ELISA for the confirmation of PEG-specificity of maternal anti-PEG IgG1, newborn anti-PEG IgG1, maternal anti-PEG IgG2, newborn anti-PEG IgG2, maternal anti-PEG IgG3, newborn anti-PEG IgG3, maternal anti-PEG IgG4, newborn anti-PEG IgG4, maternal anti-PEG IgM and newborn anti-PEG IgM, respectively.

Figure S25. Establishment of specificity cutoffs of maternal anti-PEG IgG1-4 and IgM

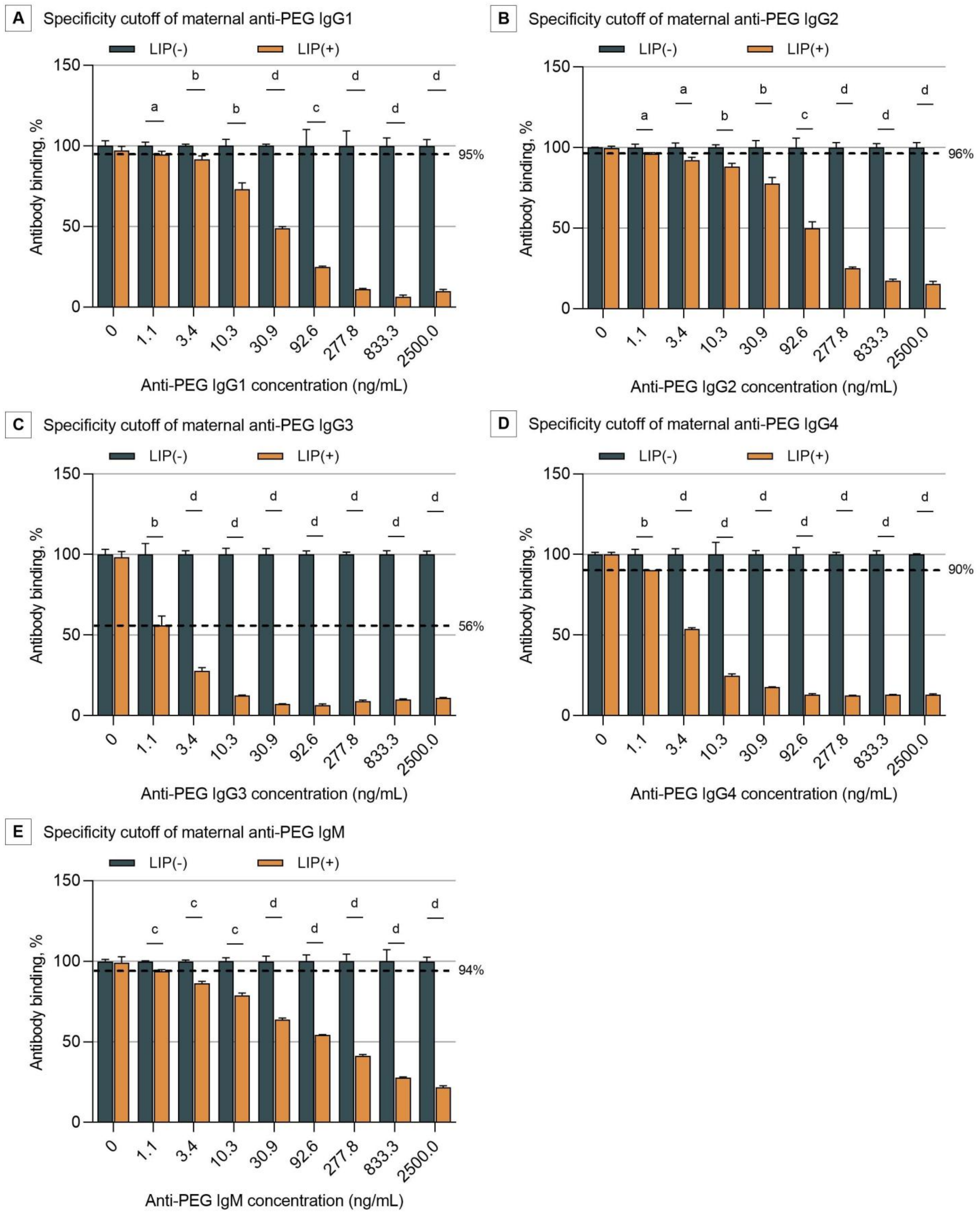

Abbreviations: LIP, PEGylated liposomal doxorubicin (competitors, see eMethods 4); LIP(-), anti-PEG antibody standards without the addition of LIP; LIP(+), anti-PEG antibody standards with the addition of LIP. Dotted lines indicate specificity cutoffs of maternal anti-PEG IgG1-4 and IgM (see eMethods 4 for establishment of specificity cutoffs of anti-PEG antibodies). All data were presented as "mean ± standard deviation" (n = 3), with differences between two groups analyzed using the two-tailed unpaired t-test. a, $P < 0.05$; b, $P < 0.01$; c, $P < 0.001$; d, $P < 0.0001$.

Figure S26. Establishment of specificity cutoffs of newborn anti-PEG IgG1-4 and IgM

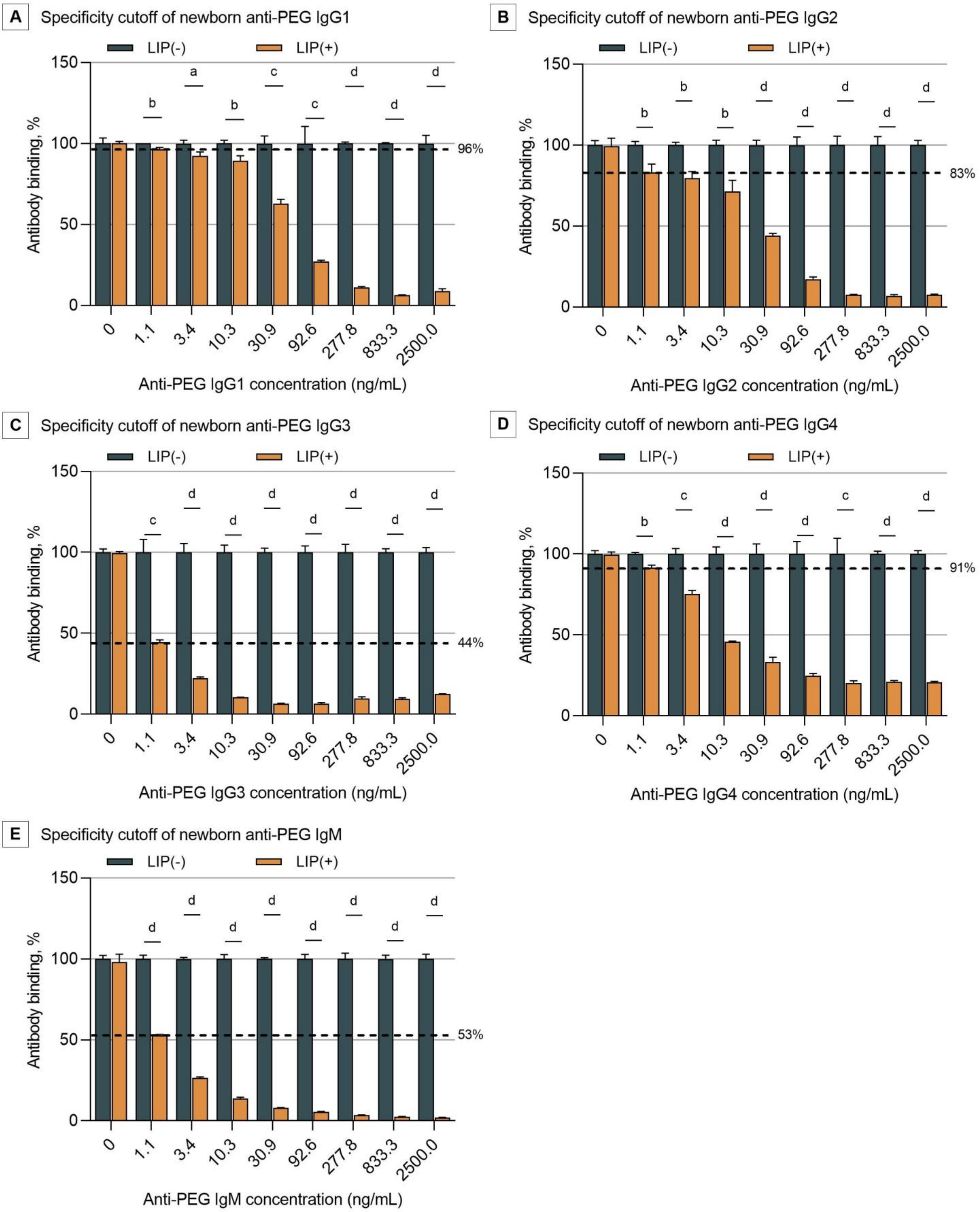

Abbreviations: LIP, PEGylated liposomal doxorubicin (competitors, see eMethods 4); LIP(-), anti-PEG antibody standards without the addition of LIP; LIP(+), anti-PEG antibody standards with the addition of LIP. Dotted lines indicate specificity cutoffs of maternal anti-PEG IgG1-4 and IgM (see eMethods 4 for establishment of specificity cutoffs of anti-PEG antibodies). All data were presented as "mean ± standard deviation" (n = 3), with differences between two groups analyzed using the two-tailed unpaired t-test. a, $P < 0.05$; b, $P < 0.01$; c, $P < 0.001$; d, $P < 0.0001$.

Figure S27. Confirmation of the PEG specificity of anti-PEG IgG1 in maternal serum samples by competitive ELISA

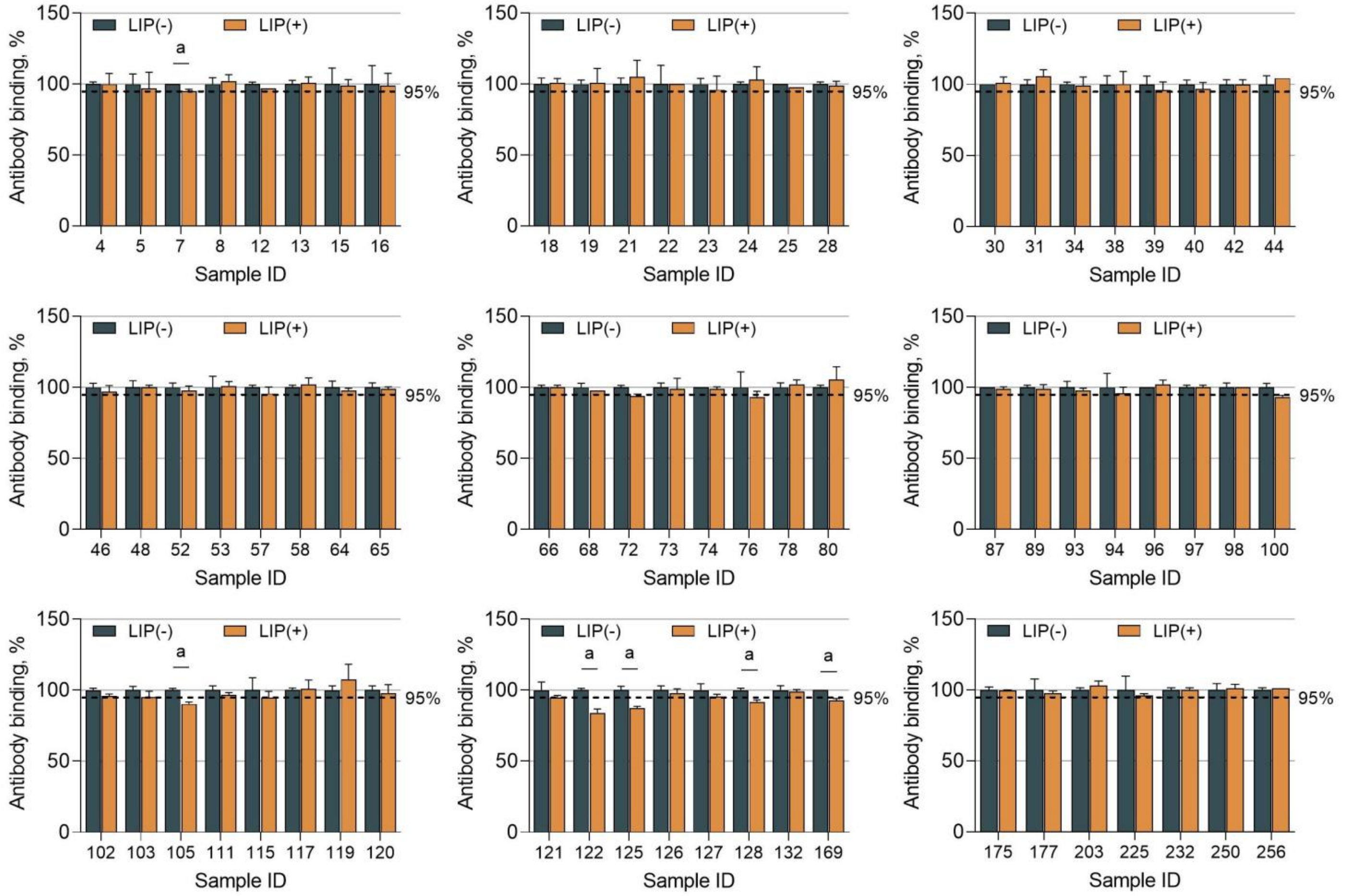

Abbreviations: LIP, PEGylated liposomal doxorubicin (competitors, see eMethods 4); LIP(-), maternal serum samples without the addition of LIP; LIP(+), maternal serum samples with the addition of LIP. Dotted lines indicate specificity cutoffs of maternal anti-PEG IgG1. All data were presented as "mean ± standard deviation" (n = 2), with differences between two groups analyzed using the two-tailed unpaired t-test. a, $P < 0.05$.

Figure S28. Confirmation of the PEG specificity of anti-PEG IgG1 in newborn serum samples by competitive ELISA

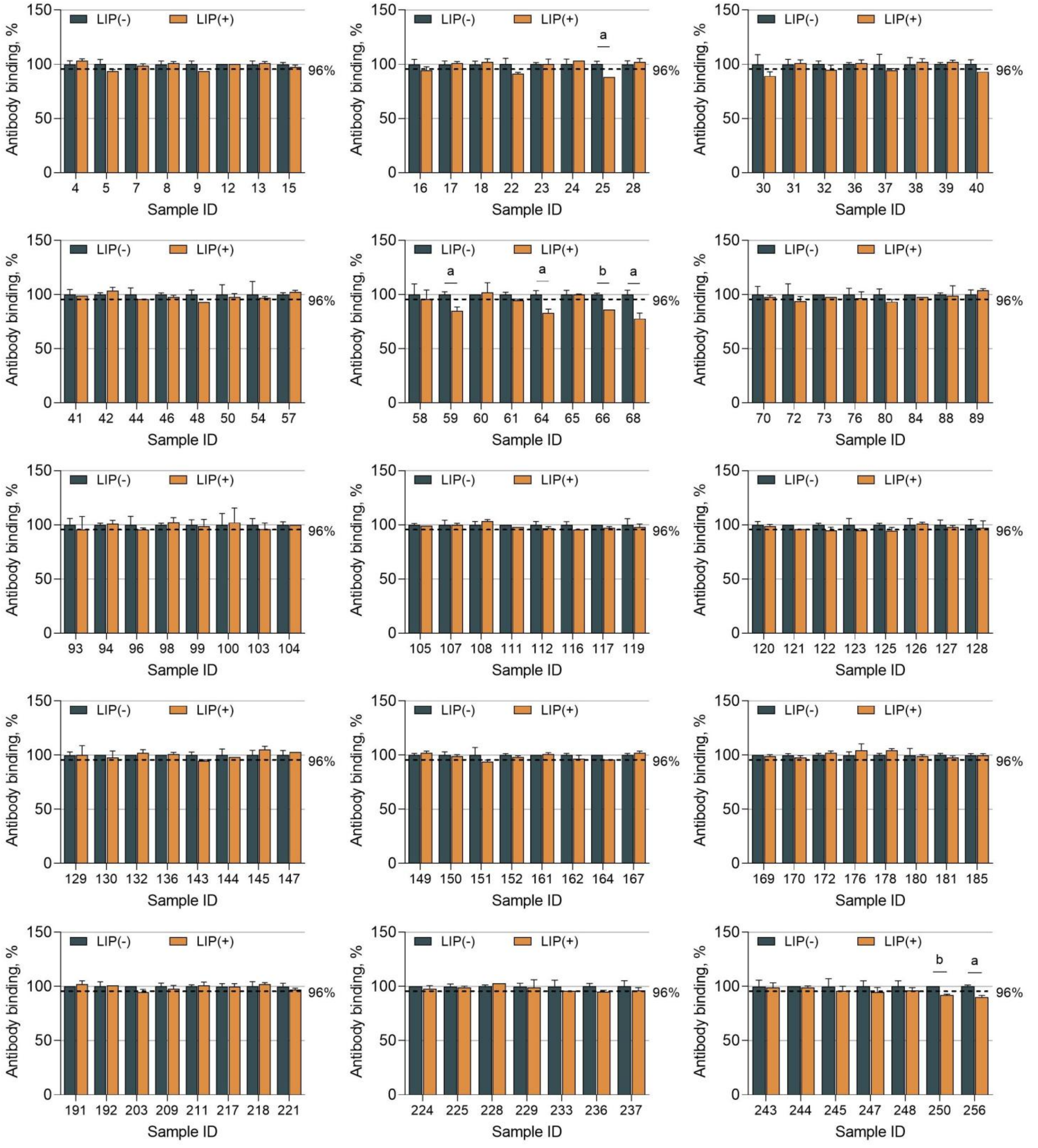

Abbreviations: LIP, PEGylated liposomal doxorubicin (competitors, see eMethods 4); LIP(-), newborn serum samples without the addition of LIP; LIP(+), newborn serum samples with the addition of LIP. Dotted lines indicate specificity cutoffs of neonatal anti-PEG IgG1. All data were presented as "mean ± standard deviation" (n = 2), with differences between two groups analyzed using the two-tailed unpaired t-test. a, $P < 0.05$; b, $P < 0.01$.

Figure S29. Confirmation of the PEG specificity of anti-PEG IgG2 in maternal serum samples by competitive ELISA

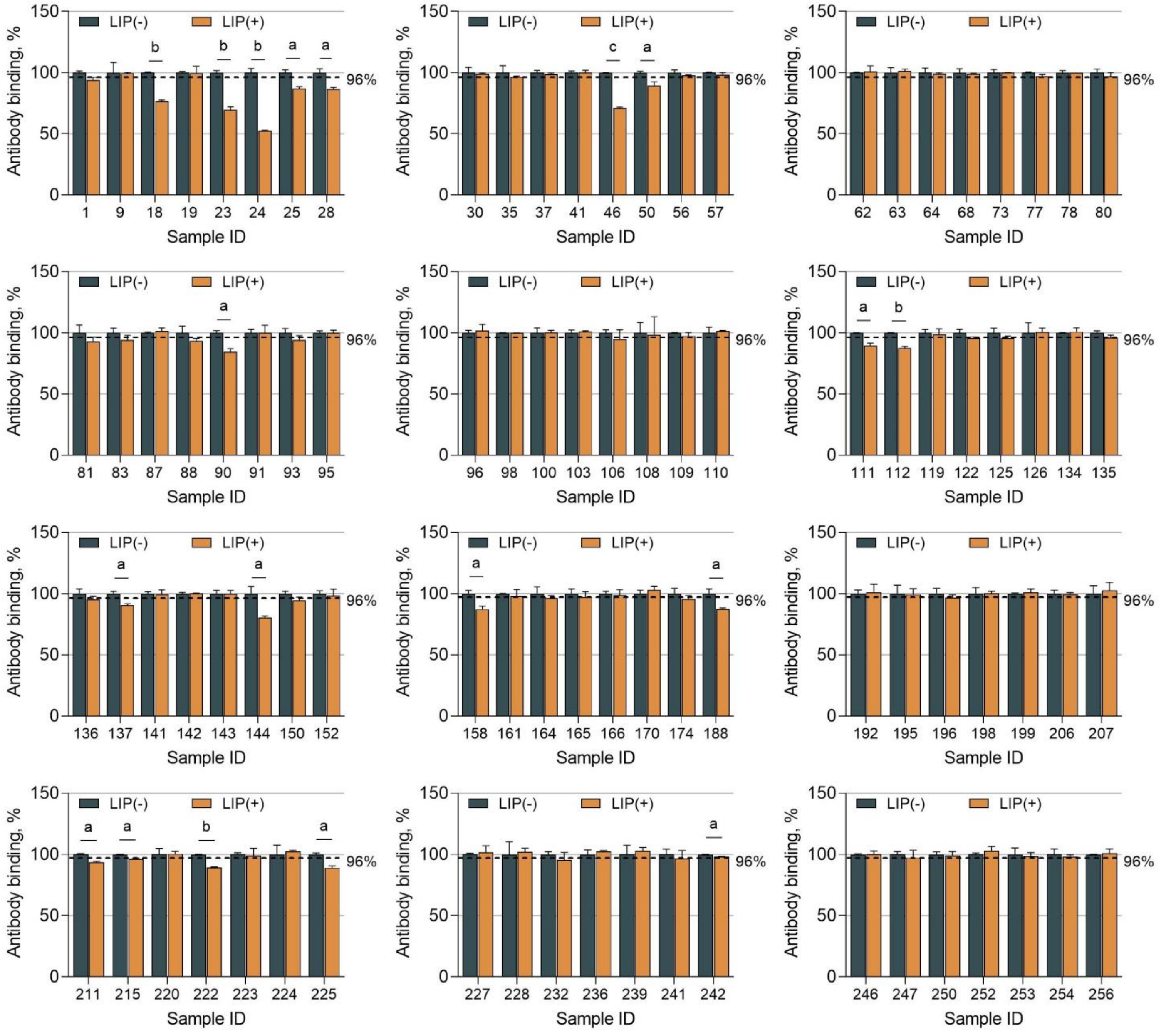

Abbreviations: LIP, PEGylated liposomal doxorubicin (competitors, see eMethods 4); LIP(-), maternal serum samples without the addition of LIP; LIP(+), maternal serum samples with the addition of LIP. Dotted lines indicate specificity cutoffs of maternal anti-PEG IgG2. All data were presented as "mean ± standard deviation" (n = 2), with differences between two groups analyzed using the two-tailed unpaired t-test. a, $P < 0.05$; b, $P < 0.01$; c, $P < 0.001$.

Figure S30. Confirmation of the PEG specificity of anti-PEG IgG2 in newborn serum samples by competitive ELISA

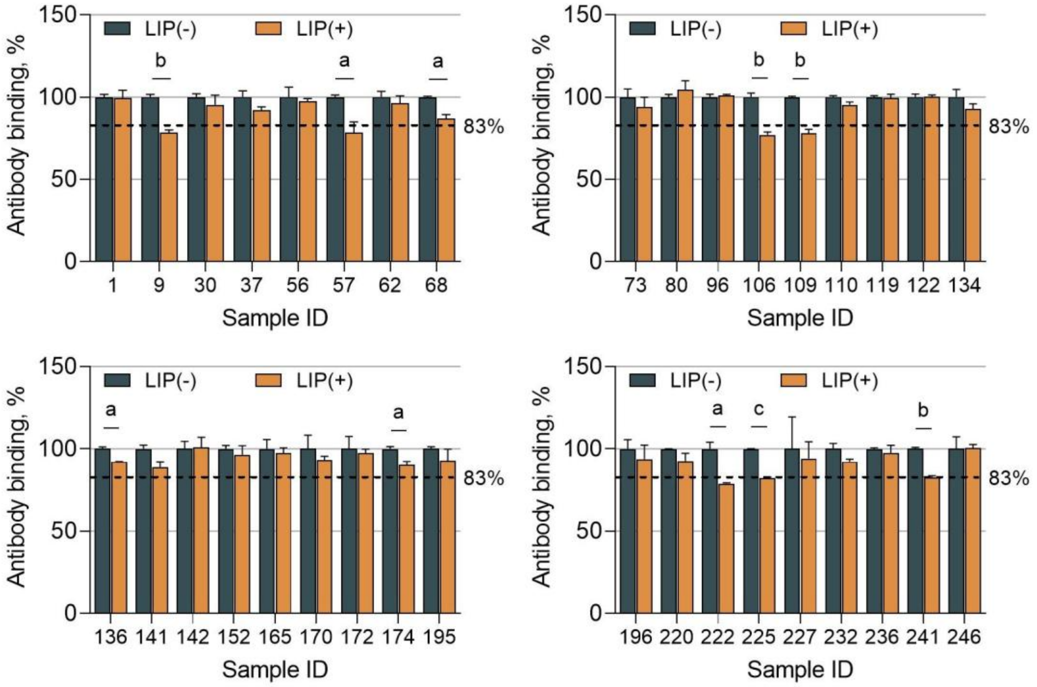

Abbreviations: LIP, PEGylated liposomal doxorubicin (competitors, see eMethods 4); LIP(-), newborn serum samples without the addition of LIP; LIP(+), newborn serum samples with the addition of LIP. Dotted lines indicate specificity cutoffs of neonatal anti-PEG IgG2. All data were presented as "mean ± standard deviation" (n = 2), with differences between two groups analyzed using the two-tailed unpaired t-test. a, $P < 0.05$; b, $P < 0.01$; c, $P < 0.001$.

Figure S31. Confirmation of the PEG specificity of anti-PEG IgG3 in maternal serum samples by competitive ELISA

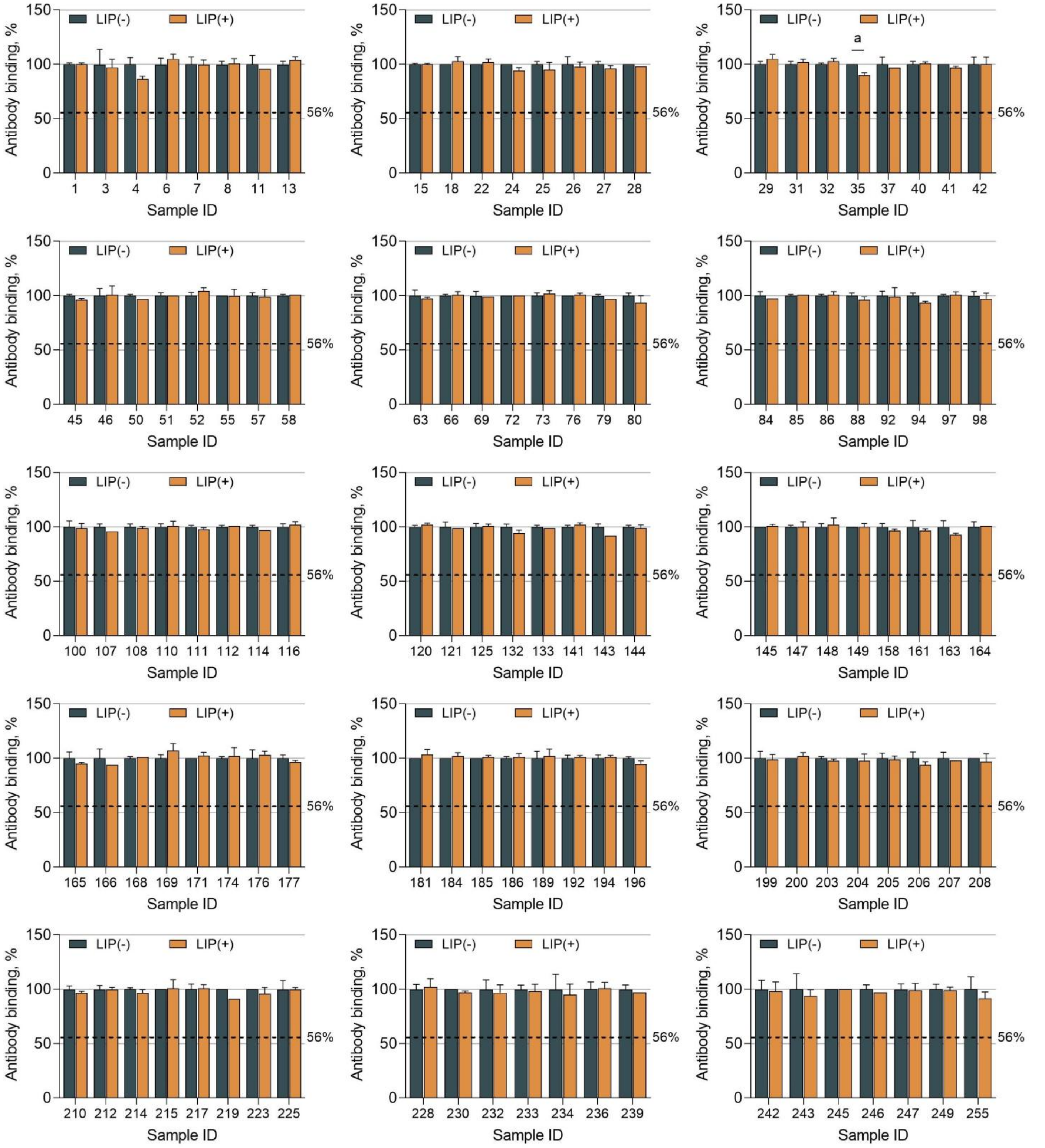

Abbreviations: LIP, PEGylated liposomal doxorubicin (competitors, see eMethods 4); LIP(-), newborn serum samples without the addition of LIP; LIP(+), newborn serum samples with the addition of LIP. Dotted lines indicate specificity cutoffs of maternal anti-PEG IgG3. All data were presented as "mean ± standard deviation" (n = 2), with differences between two groups analyzed using the two-tailed unpaired t-test. a, $P < 0.05$.

Figure S32. Confirmation of the PEG specificity of anti-PEG IgG3 in newborn serum samples by competitive ELISA

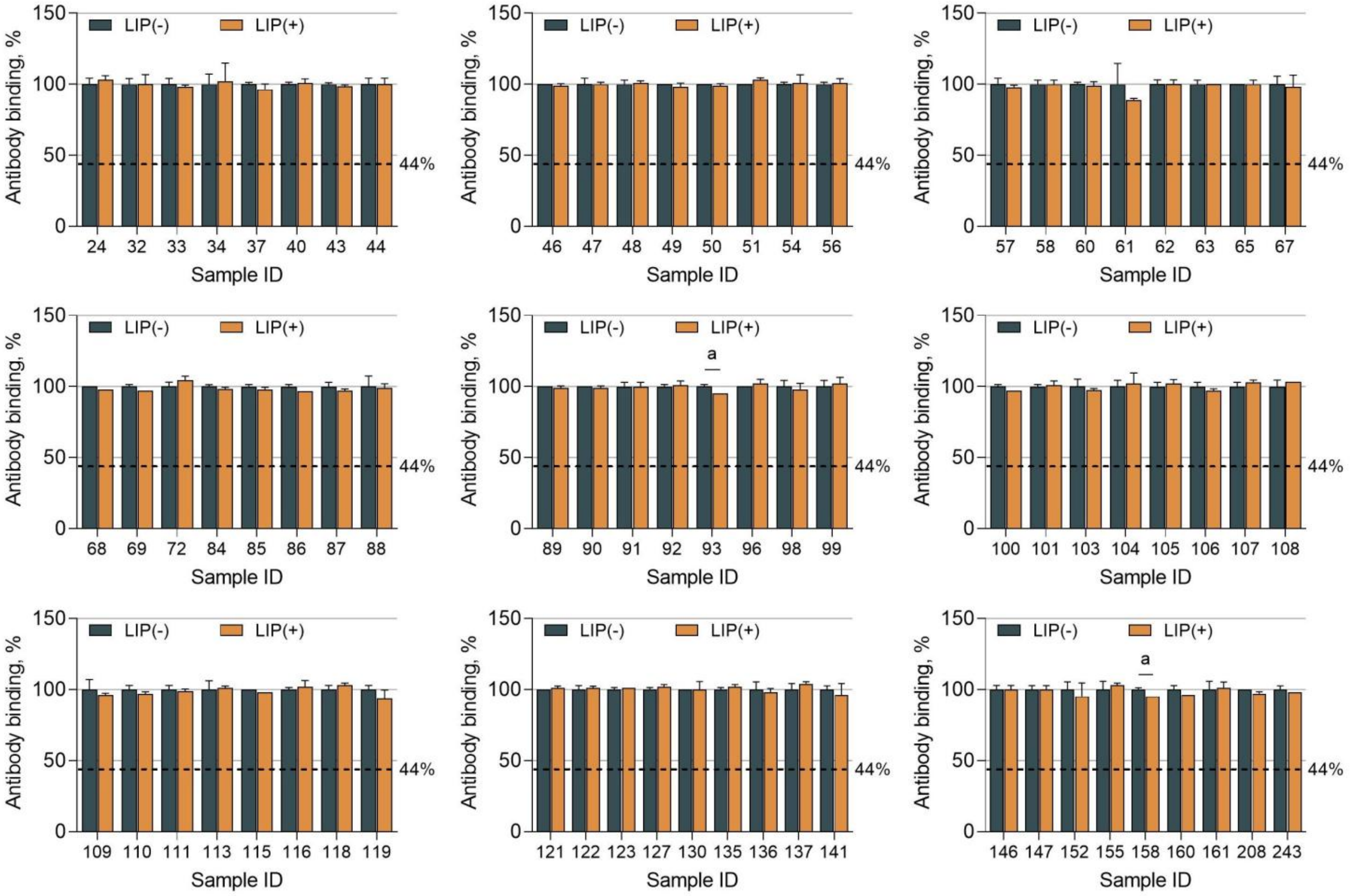

Abbreviations: LIP, PEGylated liposomal doxorubicin (competitors, see eMethods 4); LIP(-), newborn serum samples without the addition of LIP; LIP(+), newborn serum samples with the addition of LIP. Dotted lines indicate specificity cutoffs of neonatal anti-PEG IgG3. All data were presented as "mean ± standard deviation" (n = 2), with differences between two groups analyzed using the two-tailed unpaired t-test. a, $P < 0.05$.

Figure S33. Confirmation of the PEG specificity of anti-PEG IgG4 in maternal serum samples by competitive ELISA

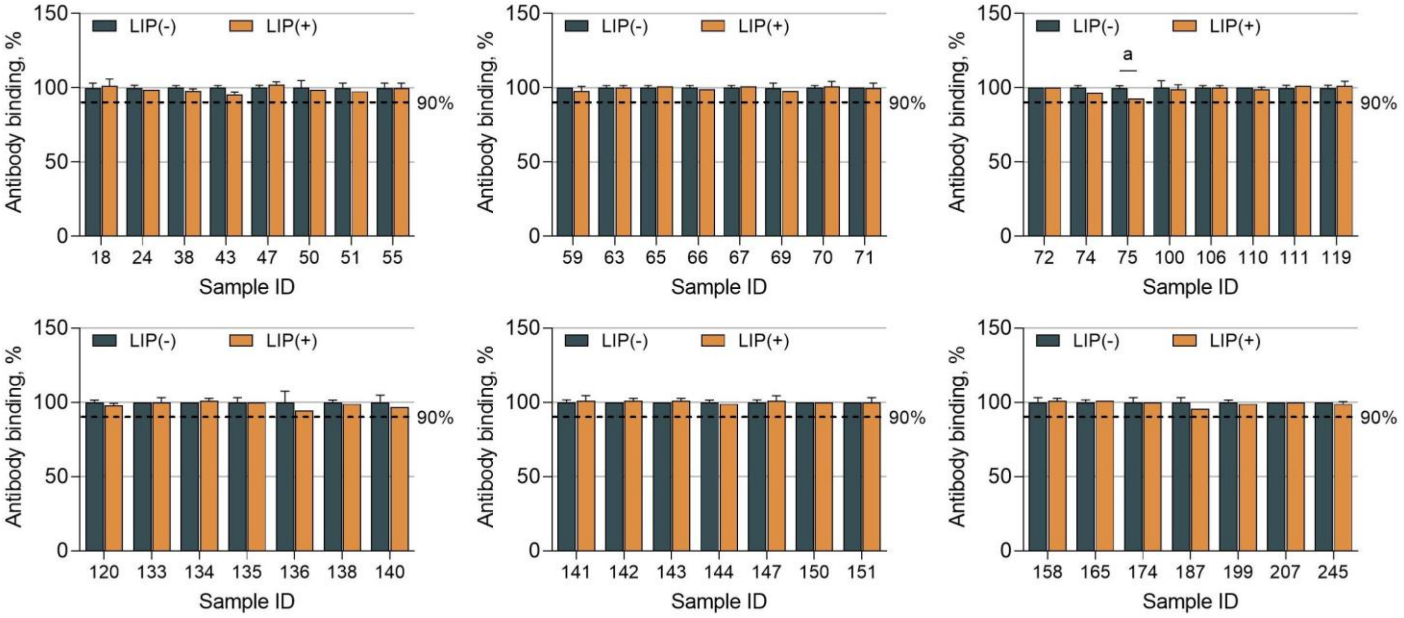

Abbreviations: LIP, PEGylated liposomal doxorubicin (competitors, see eMethods 4); LIP(-), newborn serum samples without the addition of LIP; LIP(+), newborn serum samples with the addition of LIP. Dotted lines indicate specificity cutoffs of maternal anti-PEG IgG4. All data were presented as “mean ± standard deviation” (n = 2), with differences between two groups analyzed using the two-tailed unpaired t-test. a, $P < 0.05$.

Figure S34. Confirmation of the PEG specificity of anti-PEG IgG4 in newborn serum samples by competitive ELISA

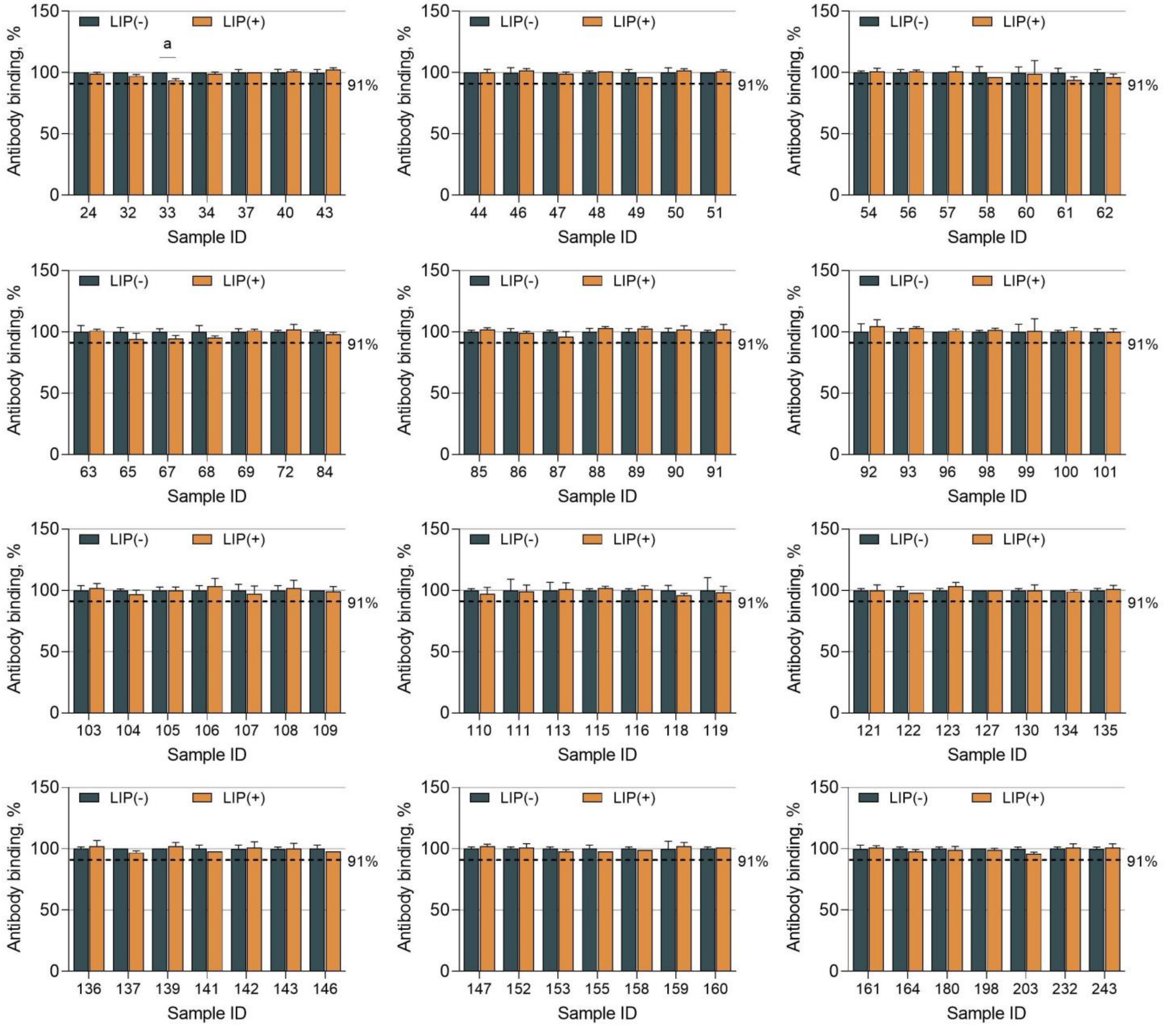

Abbreviations: LIP, PEGylated liposomal doxorubicin (competitors, see eMethods 4); LIP(-), newborn serum samples without the addition of LIP; LIP(+), newborn serum samples with the addition of LIP. Dotted lines indicate specificity cutoffs of neonatal anti-PEG IgG4. All data were presented as "mean ± standard deviation" (n = 2), with differences between two groups analyzed using the two-tailed unpaired t-test. a, $P < 0.05$.

Figure S35. Confirmation of the PEG specificity of anti-PEG IgM in maternal serum samples by competitive ELISA

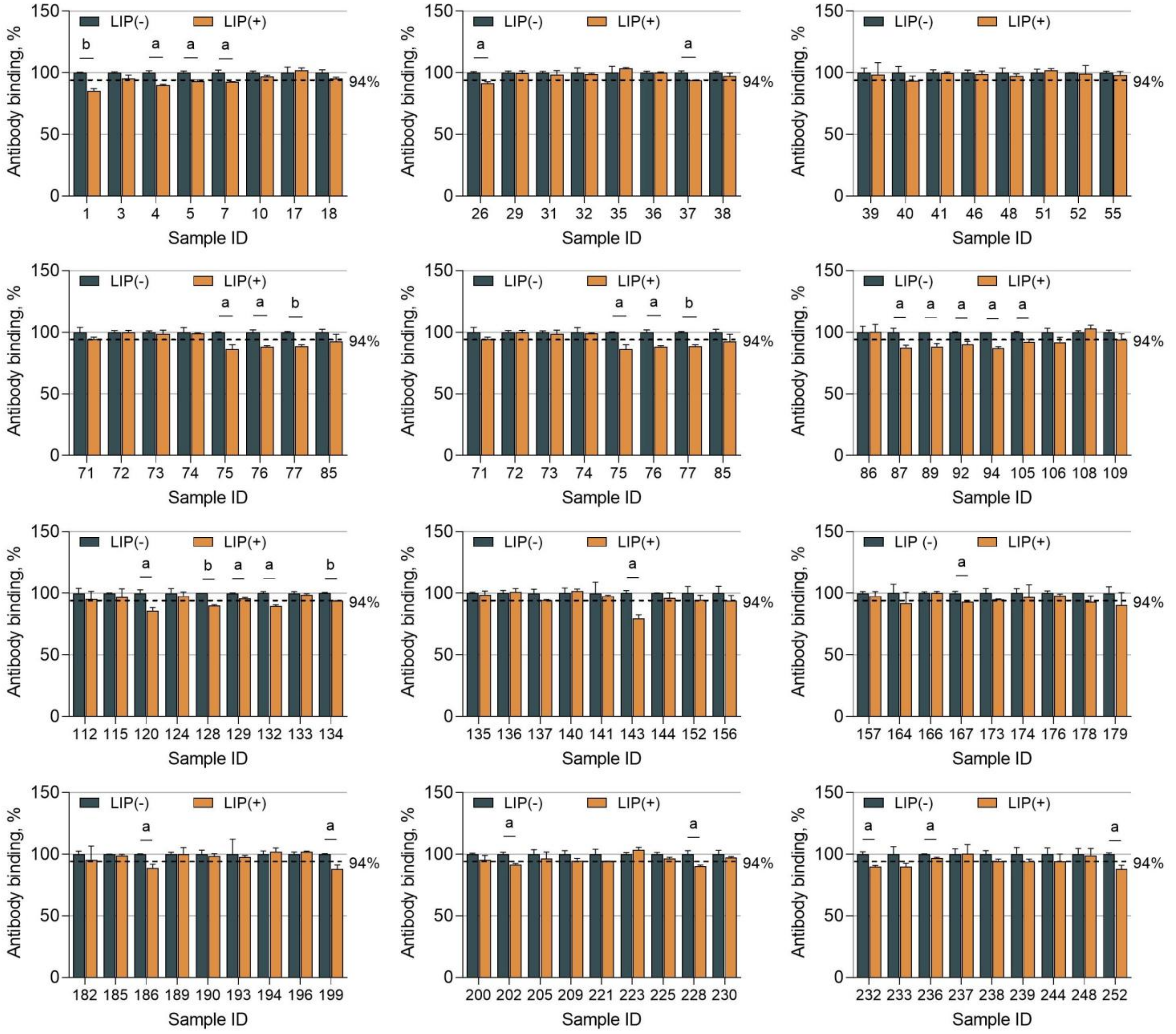

Abbreviations: LIP, PEGylated liposomal doxorubicin (competitors, see eMethods 4); LIP(-), newborn serum samples without the addition of LIP; LIP(+), newborn serum samples with the addition of LIP. Dotted lines indicate specificity cutoffs of maternal anti-PEG IgM. All data were presented as "mean ± standard deviation" (n = 2), with differences between two groups analyzed using the two-tailed unpaired t-test. a, $P < 0.05$; b, $P < 0.01$.

Figure S36. Confirmation of the PEG specificity of anti-PEG IgM in newborn serum samples by competitive ELISA

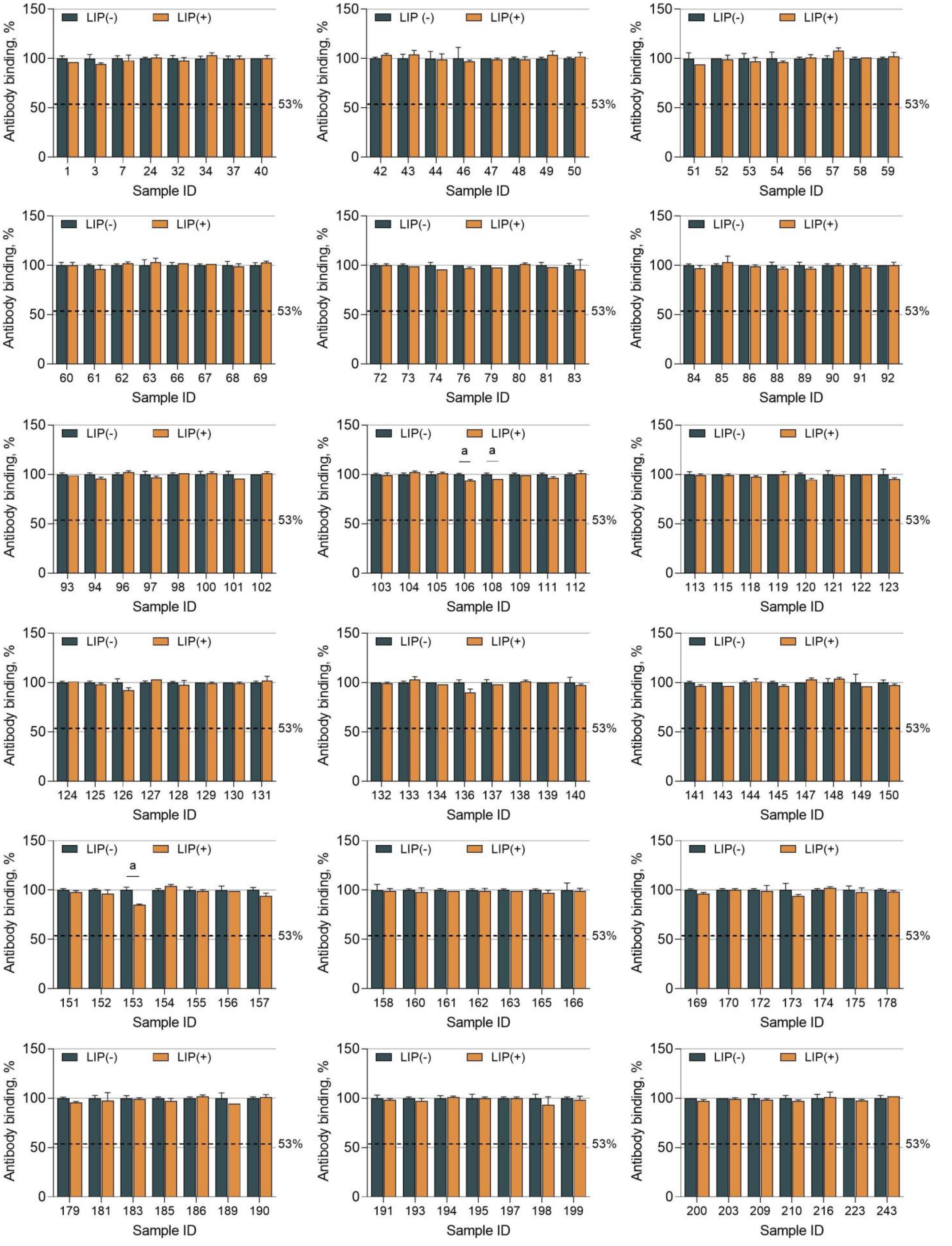

Abbreviations: LIP, PEGylated liposomal doxorubicin (competitors, see eMethods 4); LIP(-), newborn serum samples without the addition of LIP; LIP(+), newborn serum samples with the addition of LIP. Dotted lines indicate specificity cutoffs of neonatal anti-PEG IgM. All data were presented as "mean ± standard deviation" (n = 2), with differences between two groups analyzed using the two-tailed unpaired t-test. a, $P < 0.05$.

Figure S37. Standard curves of direct ELISA for detecting anti-PEG IgG1 in maternal and newborn serum samples[a]

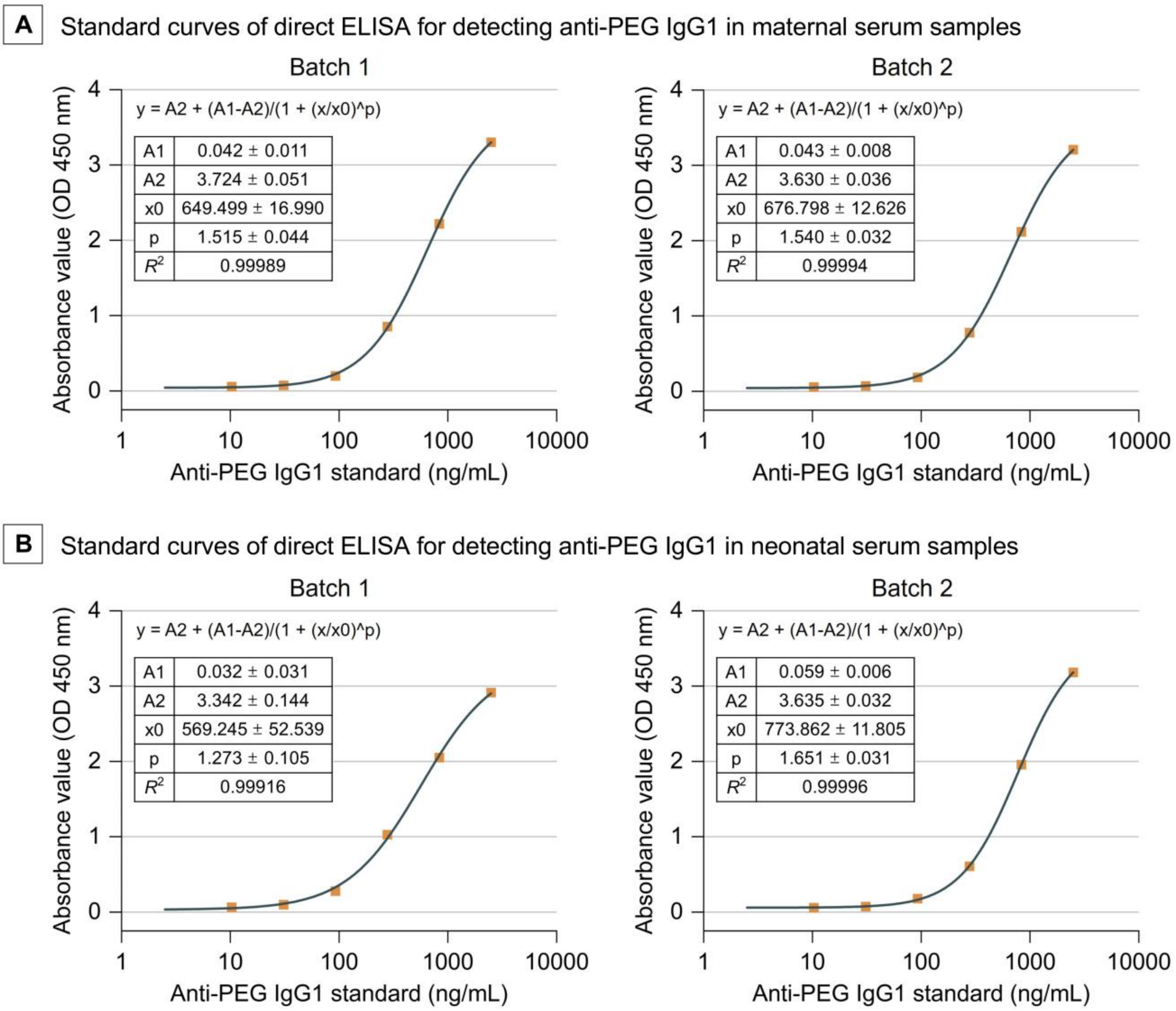

Abbreviations: R^2, coefficient of determination.

[a]Standard curves were constructed by plotting the average absorbance values (OD450 nm) and corresponding antibody concentrations with Four Parameter Logistic (4PL) curve fit using Origin 2021 software. Serial dilutions of human anti-PEG IgG1 standards (0, 10.3, 30.9, 92.6, 277.8, 833.3, and 2500.0 ng/mL) were included in each direct ELISA for two independent batches.

Figure S38. Standard curves of direct ELISA for detecting anti-PEG IgG2 in maternal and newborn serum samples[a]

A Standard curves of direct ELISA for detecting anti-PEG IgG2 in maternal serum samples

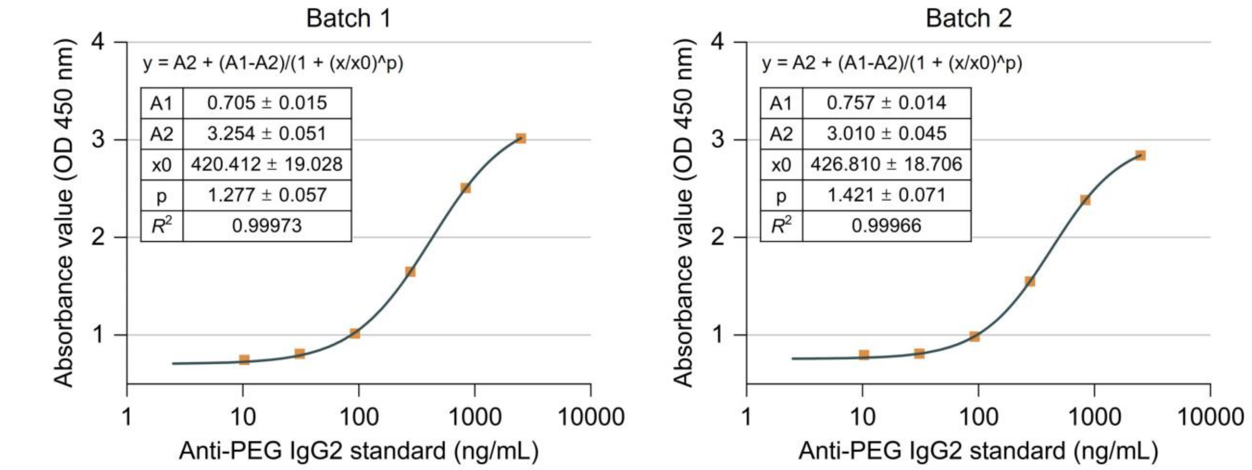

B Standard curves of direct ELISA for detecting anti-PEG IgG2 in neonatal serum samples

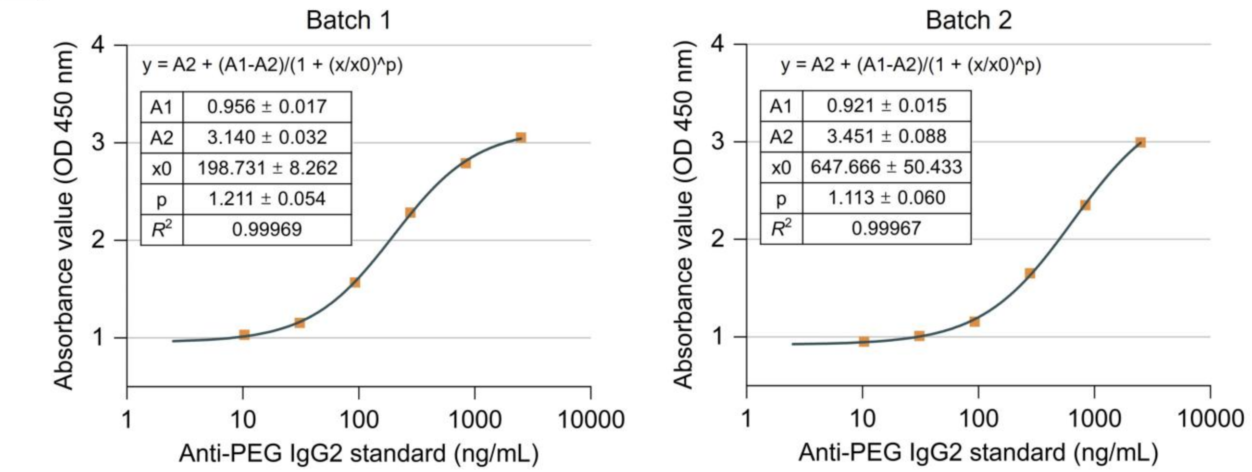

Abbreviations: R^2, coefficient of determination.

[a]Standard curves were constructed by plotting the average absorbance values (OD450 nm) and corresponding antibody concentrations with Four Parameter Logistic (4PL) curve fit using Origin 2021 software. Serial dilutions of human anti-PEG IgG2 standards (0, 10.3, 30.9, 92.6, 277.8, 833.3, and 2500.0 ng/mL) were included in each direct ELISA for two independent batches.

Figure S39. Standard curves of direct ELISA for detecting anti-PEG IgG3 in maternal and newborn serum samples[a]

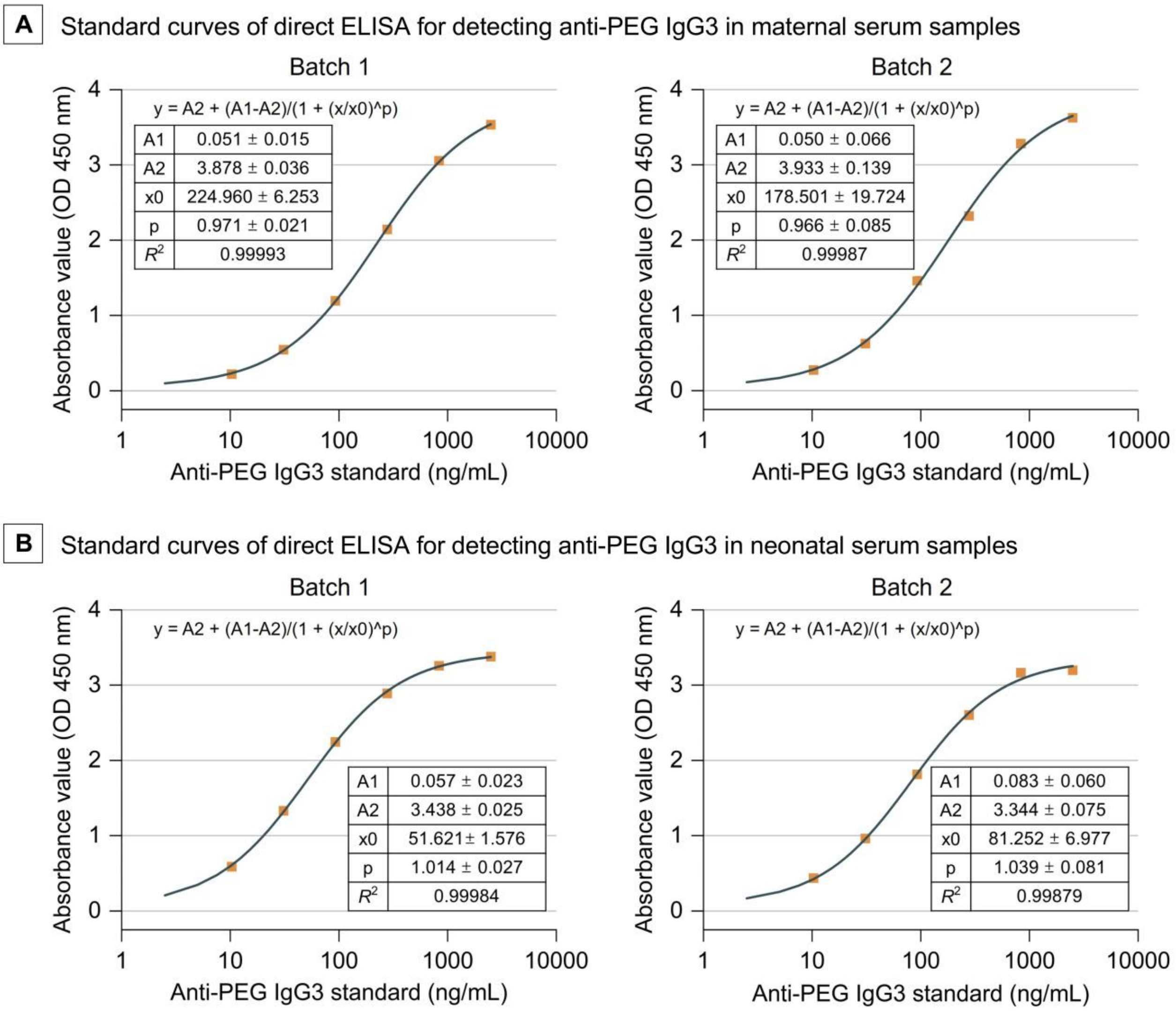

Abbreviations: R^2, coefficient of determination.

[a]Standard curves were constructed by plotting the average absorbance values (OD450 nm) and corresponding antibody concentrations with Four Parameter Logistic (4PL) curve fit using Origin 2021 software. Serial dilutions of human anti-PEG IgG3 standards (0, 10.3, 30.9, 92.6, 277.8, 833.3, and 2500.0 ng/mL) were included in each direct ELISA for two independent batches.

Figure S40. Standard curves of direct ELISA for detecting anti-PEG IgG4 in maternal and newborn serum samples[a]

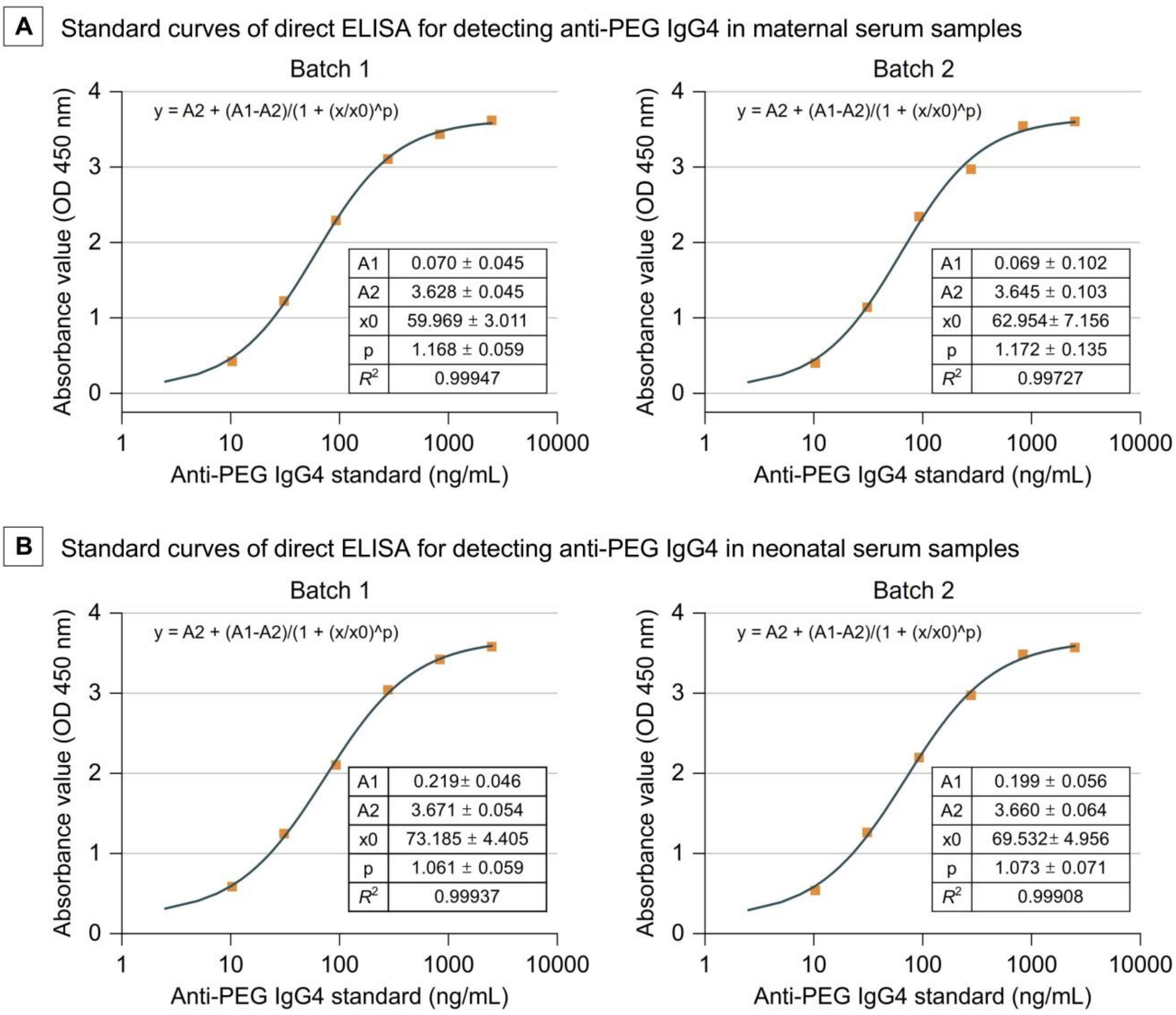

Abbreviations: R^2, coefficient of determination.

[a]Standard curves were constructed by plotting the average absorbance values (OD450 nm) and corresponding antibody concentrations with Four Parameter Logistic (4PL) curve fit using Origin 2021 software. Serial dilutions of human anti-PEG IgG4 standards (0, 10.3, 30.9, 92.6, 277.8, 833.3, and 2500.0 ng/mL) were included in each direct ELISA for two independent batches.

Figure S41. Standard curves of direct ELISA for detecting anti-PEG IgM in maternal and newborn serum samples[a]

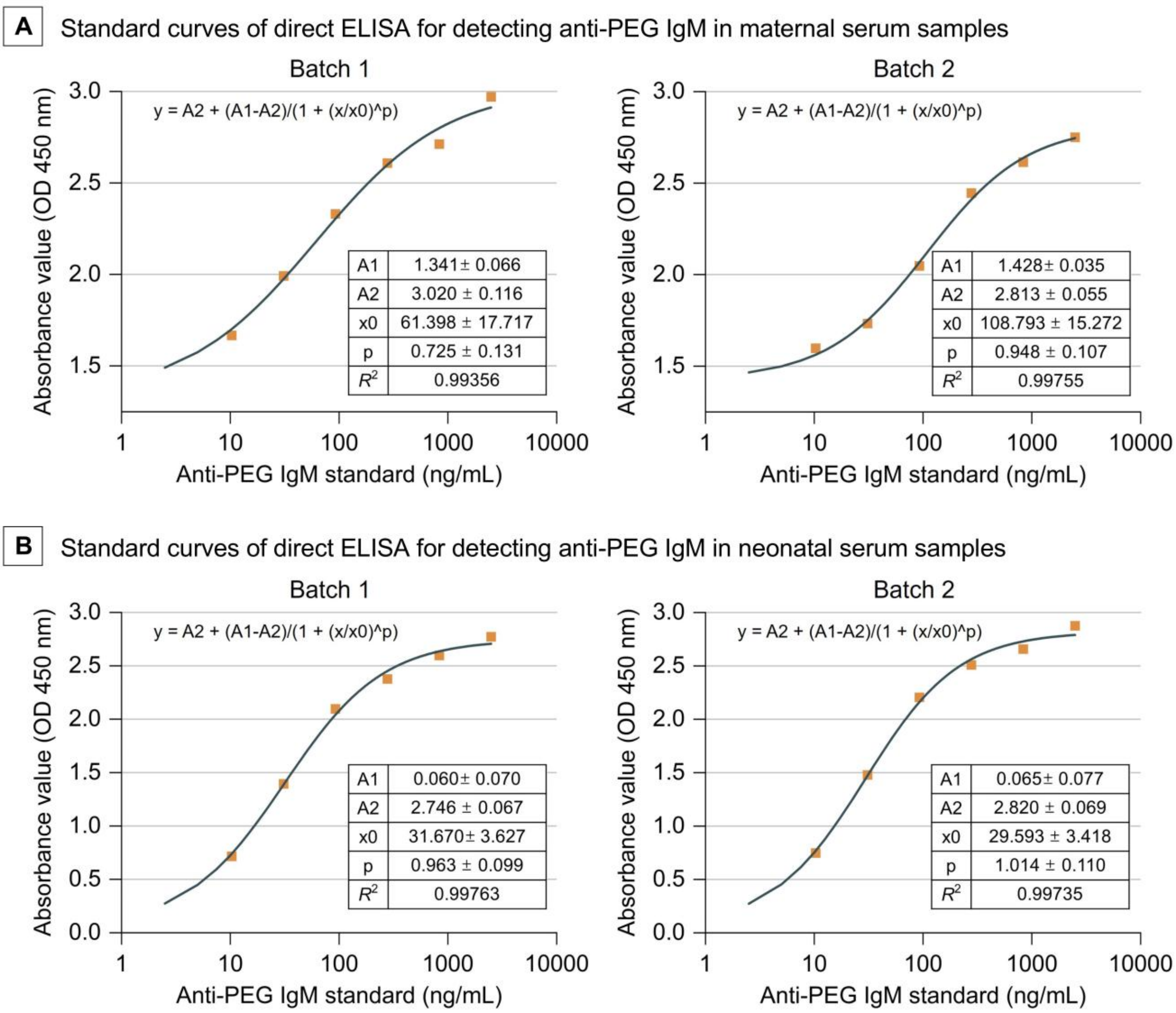

Abbreviations: R^2, coefficient of determination.

[a]Standard curves were constructed by plotting the average absorbance values (OD450 nm) and corresponding antibody concentrations with Four Parameter Logistic (4PL) curve fit using Origin 2021 software. Serial dilutions of human anti-PEG IgM standards (0, 10.3, 30.9, 92.6, 277.8, 833.3, and 2500.0 ng/mL) were included in each direct ELISA for two independent batches.

Figure S42. Standard curves of direct ELISA for detecting anti-PEG IgE in maternal and newborn serum samples[a]

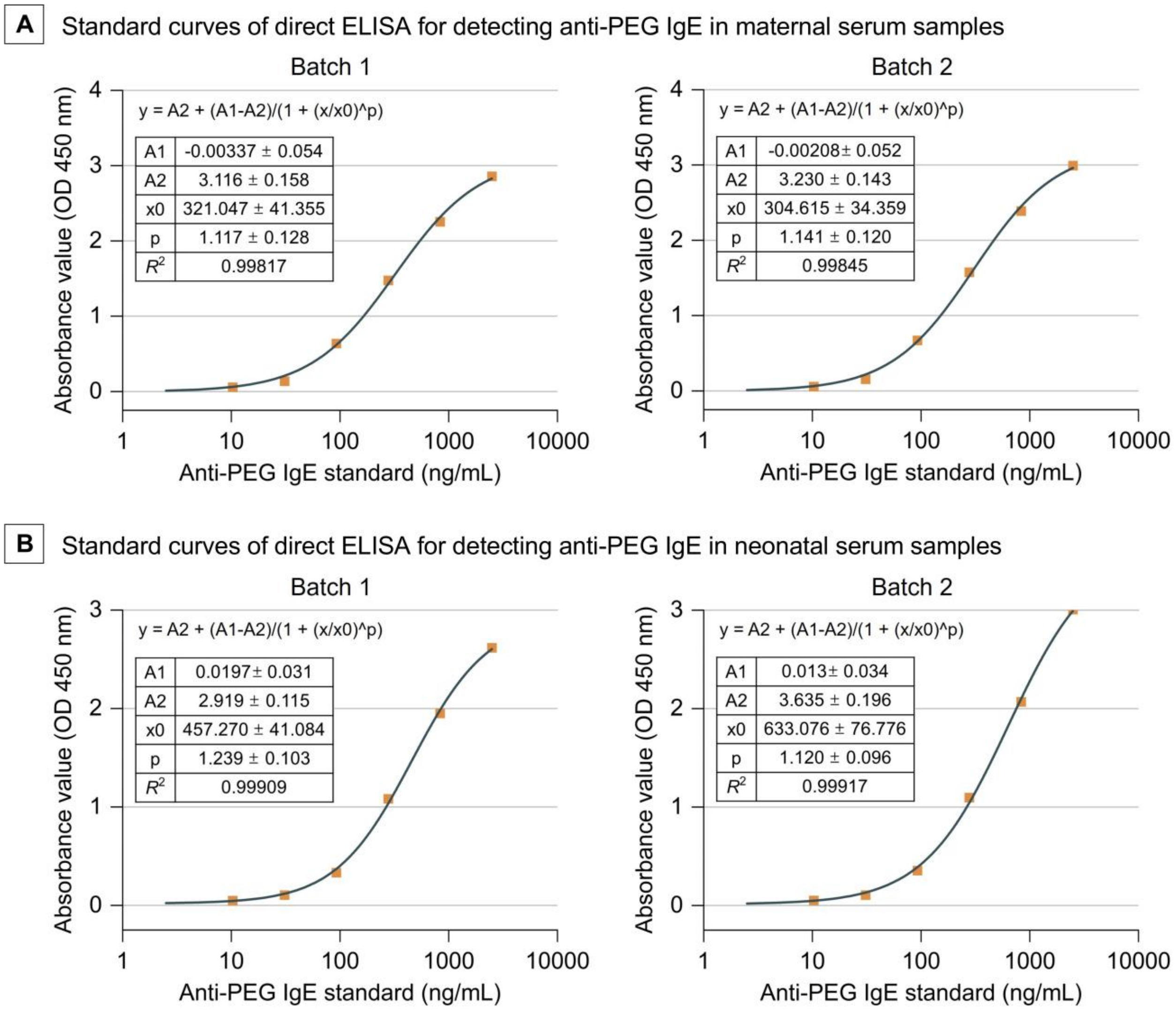

Abbreviations: R^2, coefficient of determination.

[a]Standard curves were constructed by plotting the average absorbance values (OD450 nm) and corresponding antibody concentrations with Four Parameter Logistic (4PL) curve fit using Origin 2021 software. Serial dilutions of human anti-PEG IgE standards (0, 10.3, 30.9, 92.6, 277.8, 833.3, and 2500.0 ng/mL) were included in each direct ELISA for two independent batches.

Table S7. Anti-PEG IgG1 levels in seropositive pregnant women

Sample ID	Maternal anti-PEG IgG1 levels (ng/mL)
7	259.1147
105	513.8989
122	288.6406
125	194.3535
128	183.7449
169	330.1449

Table S8. Anti-PEG IgG1 levels in seropositive newborns

Sample ID	Newborn anti-PEG IgG1 levels (ng/mL)
25	207.9177
59	163.7862
64	120.4021
66	524.5799
68	133.9695
250	1513.9751
256	481.4903

Table S9. Anti-PEG IgG2 levels in seropositive pregnant women

Sample ID	Maternal anti-PEG IgG2 levels (ng/mL)
18	868.0852
23	1412.0005
24	120.2819
25	102.2656
28	75.536
46	85.3274
50	999.4361
90	734.603
111	843.6952
112	476.9167
137	762.0953
144	292.0242
158	671.5611
188	1727.9897
211	1155.2939
215	172.0243
222	1337.3718
225	2604.887

Table S10. Anti-PEG IgG2 levels in seropositive newborns

Sample ID	Newborn anti-PEG IgG2 levels (ng/mL)
9	275.9202
57	527.0678
106	474.5477
109	1069.6205
222	275.8009
225	100.2442
241	336.5249

Table S11. Anti-PEG IgM levels in seropositive pregnant women

Sample ID	Maternal anti-PEG IgM levels (ng/mL)
1	153.8652
4	188.3983
5	93.8578
7	316.8341
26	95.7684
37	358.0414
67	177.5309
68	57.7931
75	96.4906
76	431.0382
77	225.1164
87	258.4069
89	55.4326
92	23649.1374
94	140.125
105	207.1515
120	172.6121
128	142.561
132	61.5203
134	624.2215
143	100.1753
167	89.1675
186	386.5907
199	308.9327
202	310.2522
228	1024.5648
232	58.2421
252	146.6371

Table S12. Univariable logistic regression analysis for the prevalence of pre-existing total anti-PEG antibodies in pregnant women (n = 256)

Variable	OR (95% CI)	*P* value
**Maternal age**	0.887 (0.820-0.959)	**0.003**
**BMI**[a]	1.051 (0.954-1.157)	0.314
**Education**		
Vocational degree or below	1 [Reference]	NA
Associate's degree	1.056 (0.431-2.589)	0.905
Bachelor's degree	0.717 (0.310-1.660)	0.437
Master's degree or above	0.665 (0.221-1.995)	0.467
**Permanent address**		
Town	1 [Reference]	NA
City	0.904 (0.459-1.781)	0.770
**Cosmetics use (per week)**		
0 days	1 [Reference]	NA
1-3 days	1.417 (0.566-3.545)	0.457
4-7 days	2.615 (0.974-7.026)	0.057
**Take-out food consumption (per week)**		
0 times	1 [Reference]	NA
1-3 times	3.129 (1.195-8.191)	**0.020**
4-6 times	2.987 (1.071-8.328)	**0.036**
7-9 times	4.148 (1.238-13.901)	**0.021**

Abbreviations: NA, not applicable; OR, odds ratio; CI, confidence interval. Differences were considered significant at $P < 0.05$.

Table S13. Univariable logistic regression analysis for the prevalence of pre-existing anti-PEG IgG and IgM in pregnant women (n = 256)

Variable	Anti-PEG IgM		Anti-PEG IgG	
	**OR (95% CI)**	***P* value**	**OR (95% CI)**	***P* value**
**Maternal age**	0.866 (0.783-0.958)	**0.005**	0.942 (0.852-1.041)	0.242
**BMI**[a]	1.046 (0.927-1.180)	0.463	1.003 (0.880-1.144)	0.961
**Education**				
Vocational degree or below	1 [Reference]	NA	1 [Reference]	NA
Associate's degree	0.927 (0.311-2.761)	0.892	1.167 (0.347-3.928)	0.803
Bachelor's degree	0.704 (0.255-1.939)	0.497	0.630 (0.190-2.092)	0.451
Master's degree or above	0.341 (0.067-1.746)	0.197	1.364 (0.365-5.097)	0.645
**Permanent address**				
Town	1 [Reference]	NA	1 [Reference]	NA
City	0.842 (0.362-1.957)	0.689	1.244 (0.473-3.269)	0.658
**Cosmetics use (per week)**				
0 days	1 [Reference]	NA	1 [Reference]	NA
1-3 days	0.848 (0.239-3.011)	0.798	2.021 (0.620-6.591)	0.243
4-7 days	0.879 (0.192-4.036)	0.869	5.841 (1.947-17.558)	**0.002**
**Take-out food consumption (per week)**				
0 times	1 [Reference]	NA	1 [Reference]	NA
1-3 times	2.783 (0.875-8.854)	0.083	3.477 (0.727-16.623)	0.118
4-6 times	2.026 (0.564-7.278)	0.279	4.857 (0.991-23.802)	0.051
7-9 times	2.250 (0.467-10.845)	0.312	8.500 (1.531-47.182)	**0.014**

Abbreviations: NA, not applicable; OR, odds ratio; CI, confidence interval. Differences were considered significant at $P < 0.05$.

Table S14. Univariable logistic regression analysis for the prevalence of pre-existing total anti-PEG antibodies in newborns (n = 256)

Variable	OR (95% CI)	*P* value
**Maternal age**	0.963 (0.848-1.094)	0.563
**Maternal BMI**[a]	1.113 (0.949-1.305)	0.189
**Maternal education**		
Vocational degree or below	1 [Reference]	NA
Associate's degree	0.807 (0.191-3.404)	0.770
Bachelor's degree	0.564 (0.145-2.197)	0.446
Master's degree or above	0.311 (0.033-2.901)	0.305
**Maternal permanent address**		
Town	1 [Reference]	NA
City	0.521 (0.174-1.558)	0.243
**Maternal cosmetics use (per week)**		
0 days	1 [Reference]	NA
1-3 days	0.000 (0.000-0.000)	0.998
4-7 days	7.259 (2.159-24.411)	**0.001**
**Maternal take-out food consumption (per week)**		
0 times	1 [Reference]	NA
1-3 times	1.473 (0.355-6.100)	0.594
4-6 times	1.893 (0.434-8.261)	0.396
7-9 times	0.000 (0.000-0.000)	0.998
**Gestational age at delivery**	0.874 (0.461-1.656)	0.679
**Newborn gender**		
Male	1 [Reference]	NA
Female	0.726 (0.244-2.154)	0.563
**Newborn weight**	2.411 (0.616-9.436)	0.206

Abbreviations: NA, not applicable; OR, odds ratio; CI, confidence interval. Differences were considered significant at $P < 0.05$.

Figure S43. Correlation between the levels of pre-existing total anti-PEG antibodies, anti-PEG IgG and IgM with maternal age and BMI in pregnant women seropositive for anti-PEG antibodies (n = 49), anti-PEG IgG (n = 24) and IgM (n = 28)

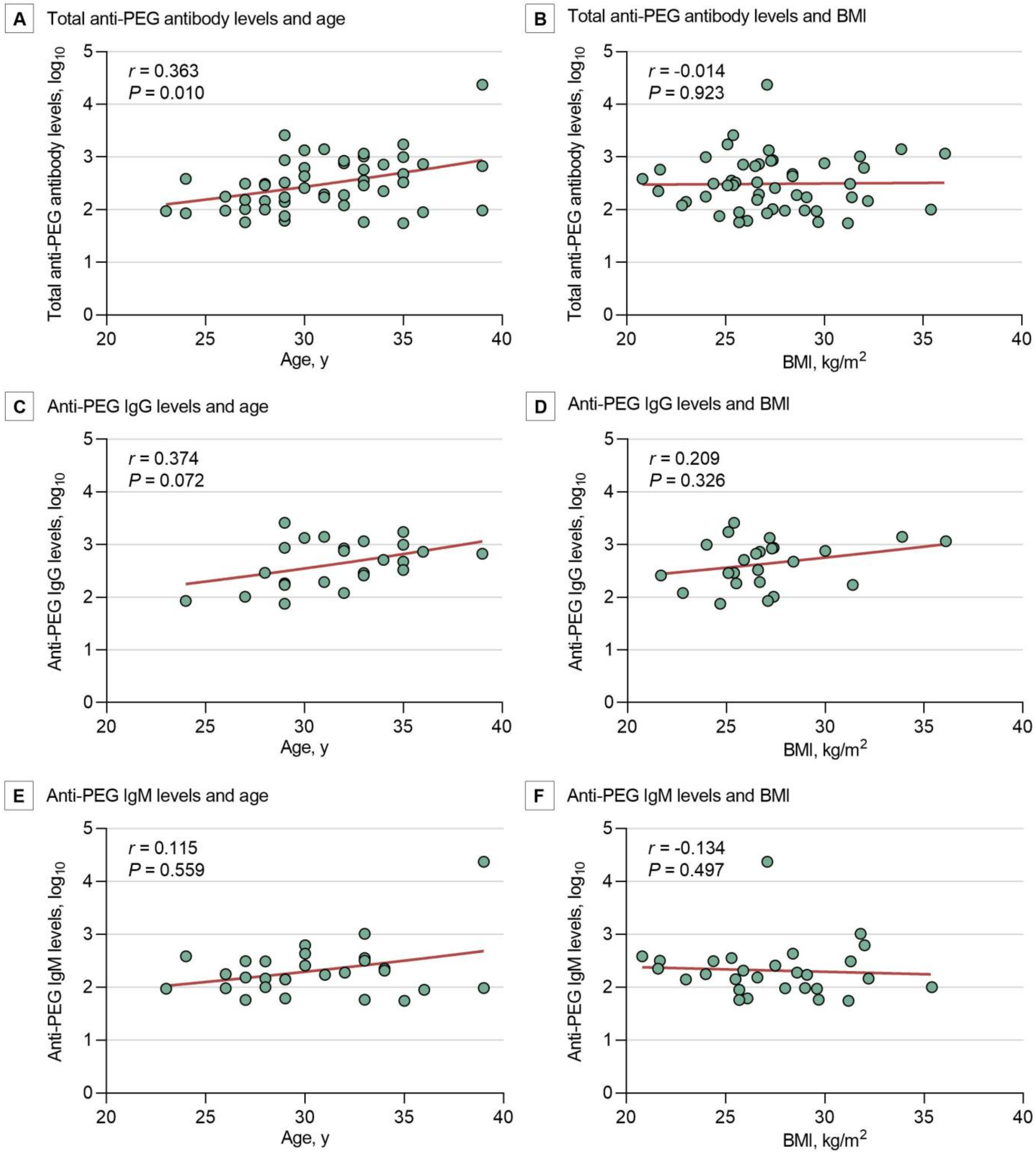

Abbreviations: *r*, correlation coefficients. For pregnant women seropositive for anti-PEG antibodies, correlations between anti-PEG antibody levels after $\log_{10}$ transformation and each continuous variable (e.g., maternal age and BM) were analyzed using Spearman correlation coefficients (*r*). Differences were considered significant at $P < 0.05$.

Table S15. Univariable generalized linear regression analysis for the levels of pre-existing total anti-PEG antibodies in pregnant women seropositive for anti-PEG antibodies (n = 49)

Variable	β (95% CI)	*P* value
**Maternal age**	0.053 (0.018-0.088)	**0.003**
**BMI**[a]	0.002 (-0.041-0.045)	0.918
**Education**		
Vocational degree or below	0 [Reference]	NA
Associate's degree	0.050 (-0.350-0.449)	0.808
Bachelor's degree	0.243 (-0.134-0.621)	0.206
Master's degree or above	0.040 (-0.463-0.542)	0.877
**Permanent address**		
Town	0 [Reference]	NA
City	0.158 (-0.151-0.467)	0.317
**Cosmetics use (per week)**		
0 days	0 [Reference]	NA
1-3 days	0.163 (-0.221-0.547)	0.406
4-7 days	0.576 (0.191-0.960)	**0.003**
**Take-out food consumption (per week)**		
0 times	0 [Reference]	NA
1-3 times	0.160 (-0.302-0.622)	0.498
4-6 times	0.141 (-0.349-0.630)	0.573
7-9 times	0.113 (-0.445-0.671)	0.691

Abbreviations: NA, not applicable; β, standardized regression coefficient; CI, confidence interval. Differences were considered significant at $P < 0.05$.

Table S16. Univariable generalized linear regression analysis for the levels of pre-existing anti-PEG IgG and IgM in pregnant women seropositive for anti-PEG IgG (n = 24) and IgM (n = 28)

Variable	Anti-PEG IgM		Anti-PEG IgG	
	**β (95% CI)**	***P* value**	**β (95% CI)**	***P* value**
**Maternal age**	0.039 (-0.006-0.084)	0.087	0.057 (0.010-0.104)	**0.018**
**BMI**	-0.009 (-0.063-0.044)	0.737	0.039 (-0.013-0.091)	0.143
**Education**				
Vocational degree or below	0 [Reference]	NA	0 [Reference]	NA
Associate's degree	0.173 (-0.304-0.650)	0.477	-0.114 (-0.577-0.348)	0.628
Bachelor's degree	0.453 (0.005-0.900)	**0.047**	0.064 (-0.398-0.527)	0.786
Master's degree or above	0.321 (-0.418-1.060)	0.395	-0.346 (-0.845-0.154)	0.175
**Permanent address**				
Town	0 [Reference]	NA	0 [Reference]	NA
City	0.224 (-0.169-0.617)	0.263	-0.085 (-0.481-0.310)	0.673
**Cosmetics use (per week)**				
0 days	0 [Reference]	NA	0 [Reference]	NA
1-3 days	0.121 (-0.358-0.600)	0.621	0.095 (-0.381-0.571)	0.696
4-7 days	1.220 (0.645-1.795)	**< 0.001**	0.057 (-0.353-0.466)	0.786
**Take-out food consumption (per week)**				
0 times	0 [Reference]	NA	0 [Reference]	NA
1-3 times	-0.188 (-0.733-0.358)	0.500	0.702 (0.107-1.297)	**0.021**
4-6 times	-0.224 (-0.827-0.379)	0.466	0.595 (-0.006-1.196)	0.053
7-9 times	-0.500 (-1.235-0.234)	0.182	0.642 (0.006-1.279)	**0.048**

Abbreviations: NA, not applicable; β, standardized regression coefficient; CI, confidence interval. Differences were considered significant at $P < 0.05$.

Figure S44. Correlation between the levels of pre-existing total anti-PEG antibodies and maternal age, maternal BMI, gestational age at delivery, and newborn weight in newborns seropositive for anti-PEG antibodies (n = 14)

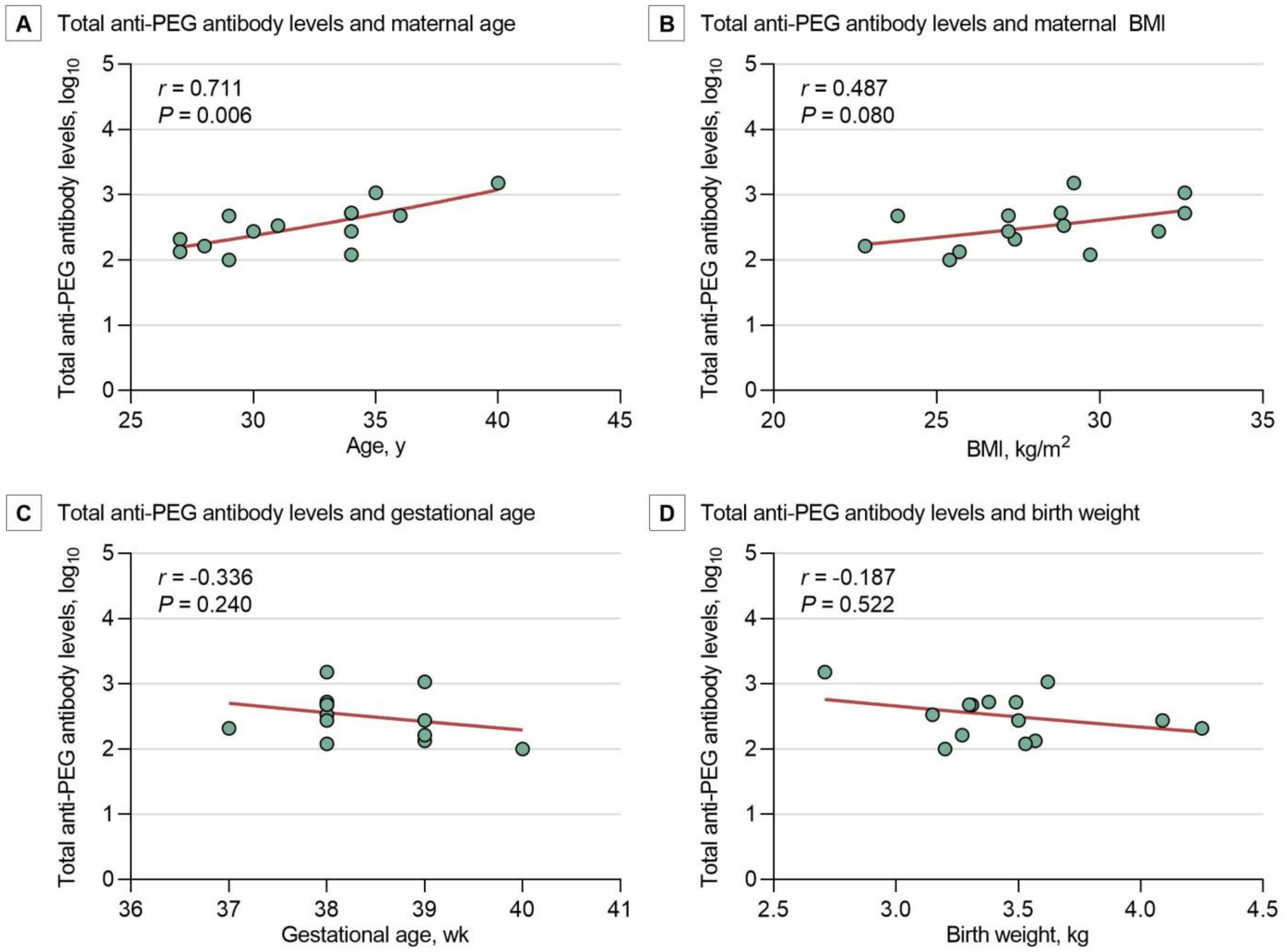

Abbreviations: *r*, correlation coefficients. For newborns seropositive for anti-PEG antibodies, correlations between anti-PEG antibody levels after $\log_{10}$ transformation and each continuous variable (e.g., maternal age, maternal BMI, gestation age at delivery, and newborn weight) were analyzed using Spearman correlation coefficients (*r*). Differences were considered significant at $P < 0.05$.

Table S17. Univariable generalized linear regression analysis for the levels of pre-existing total anti-PEG antibodies in newborns seropositive for anti-PEG antibodies (n = 14)

Variable	β (95% CI)	*P* value
**Maternal age**	0.065 (0.032-0.098)	**< 0.001**
**Maternal BMI**	0.053 (-5.978E-5-0.106)	0.050
**Maternal education**		
Vocational degree or below	0 [Reference]	NA
Associate's degree	0.102 (-0.318-0.523)	0.633
Bachelor's degree	-0.110 (-0.509-0.289)	0.590
Master's degree or above	-0.475 (-1.139-0.190)	0.162
**Maternal permanent address**		
Town	0 [Reference]	NA
City	-0.254 (-0.585-0.077)	0.133
**Maternal cosmetics use (per week)**		
0 days	0 [Reference]	NA
4-7 days	-0.199 (-0.552-0.154)	0.269
**Maternal take-out food consumption (per week)**		
0 times	0 [Reference]	NA
1-3 times	-0.189 (-0.635-0.257)	0.406
4-6 times	-0.271 (-0.731-0.190)	0.250
**Gestational age at delivery**	-0.147 (-0.381-0.086)	0.217
**Newborn gender**		
Male	0 [Reference]	NA
Female	0.074 (-0.281-0.428)	0.684
**Newborn weight**	-0.303 (-0.757-0.152)	0.192

Abbreviations: NA, not applicable; β, standardized regression coefficient; CI, confidence interval. Differences were considered significant at $P < 0.05$.

Figure S45. Overview diagram describing the design, procedures and major research findings of this study

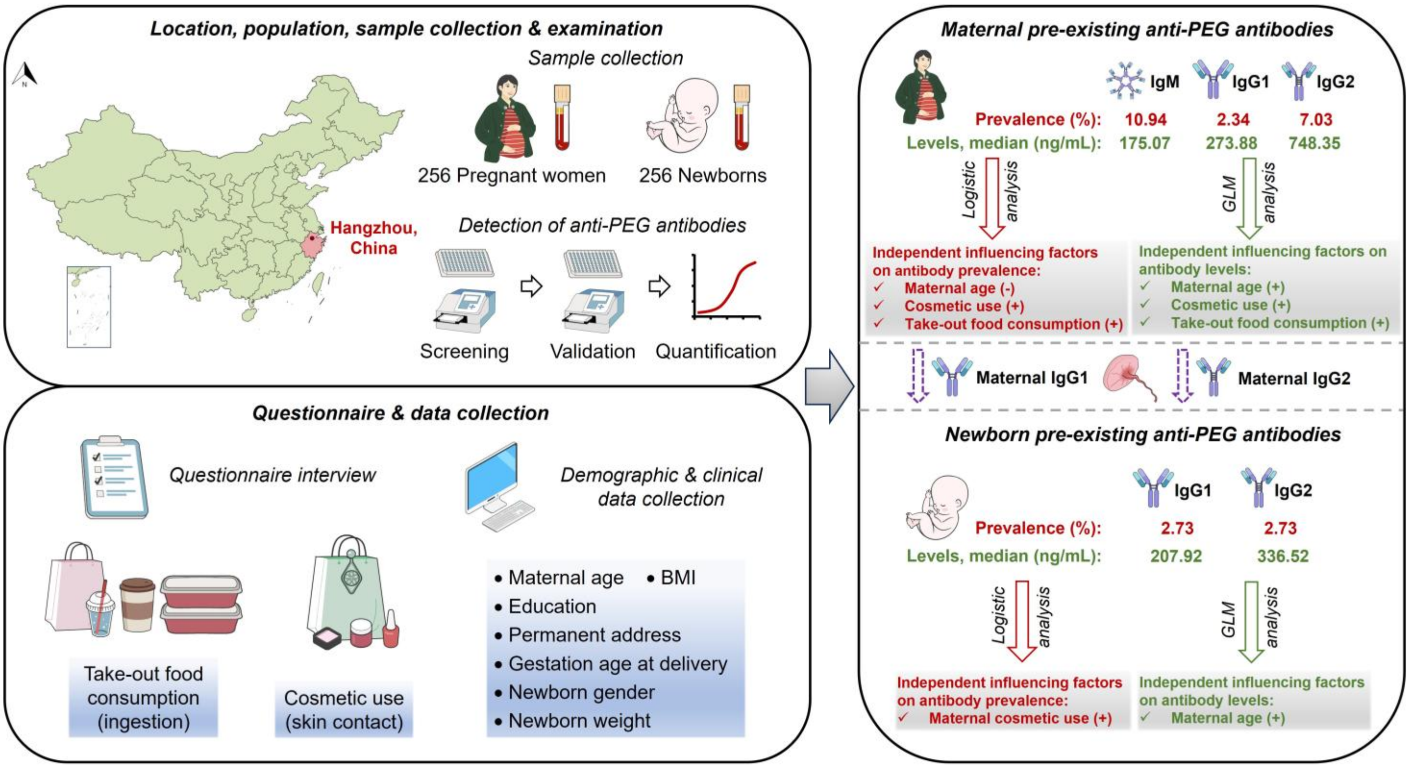

Abbreviations: (-), negative association with antibody prevalence; (+), positive association with antibody prevalence or levels. 256 pregnant women admitted for delivery at the Women's Hospital, Zhejiang University School of Medicine in China and their newborns were enrolled in this cross-sectional study, with corresponding maternal and cord blood samples carefully collected and examined for pre-existing anti-PEG antibodies. Using internationally recognized direct ELISA and competitive ELISA, along with questionnaire interviews, demographic and clinical data collections, and in-depth statistical analysis, the seropositivities and levels of pre-existing anti-PEG antibodies in pregnant women and corresponding newborns were revealed. Moreover, we found that maternal age, take-out food consumption, and cosmetic use were influencing factors of prevalence and levels of maternal anti-PEG antibodies, while the prevalence or levels of newborn anti-PEG antibodies were affected by maternal age and maternal cosmetic use. As fetuses are incapable of synthesizing IgG antibodies, the presence of pre-existing anti-PEG IgG1 and IgG2 in newborns may suggest active maternal-fetal antibody transfers across the placenta.

Table S18. Preclinical and clinical studies indicating induction of anti-PEG antibodies by topical application of lotion products containing PEG derivatives

Year[Ref]	Cosmetic product /Manufacturer	PEG/PEG-derivatives contained in cosmetics	Treatment design	Anti-PEG antibody detected
Preclinical study				
2024[44]	Bioderma Sébium Lotion /Bioderma Co.	PEG-11 methyl-ether-dimethicone and PEG-40 hydrogenated castor oil	Daily topical applications of 100 μL of a cosmetic product for 7 days on shaved compromised dorsal skin in mice	Anti-PEG IgM
2023[45]	AquaLabel Balance Care Lotion /Shiseido Japan Co.	polyoxyethylene (14) polyoxypropylene (7) dimethyl ether and polyoxyethylene (17) polyoxypropylene (4) and dimethyl ether	Daily topical applications of 50 μL of a cosmetic product for 5 days on intact skin and compromised skin, respectively, in mice	Anti-PEG IgM
2023[45]	Bioderma Sébium Lotion /Bioderma Co.	PEG-11 methyl-ether-dimethicone and PEG-40 hydrogenated castor oil	Daily topical applications of 50 μL of a cosmetic product for 5 days on intact skin and compromised skin, respectively, in mice	Anti-PEG IgM
Clinical study				
2023[46]	NA	NA	NA	Anti-PEG IgM[#]

NA, not applicable. [#] This study only confirmed that the plasma level of anti-PEG IgM was significantly higher in frequent cosmetic users compared to rare users. Additionally, this study did not explicitly specify the types of cosmetics used or the types of PEG/PEG derivatives contained in the cosmetics.

Table S19. Representative preclinical studies indicating accelerated blood clearance of PEGylated Drugs

Year[Ref]	Animal model	Treatment design: PEGylated agent for initial dose	Treatment design: PEGylated agent for subsequent dose	Treatment design: Dosing interval	Isotype of anti-PEG antibodies induced by the initial dose
2006 (4)	Rat	PEGylated liposomes (IV)	Same as initial dose	4, 5, 6, 7, 10 and 14 days	Anti-PEG IgM
2007 (5)	Rat	PEGylated liposomes (IV)	Same as initial dose	5 days	Anti-PEG IgM
2009 (14)	Rat	PEG-modified PLA nanoparticles (IV)	Same as initial dose	3, 5, 7 and 14 days	Anti-PEG IgM
2012 (15)	Mice	PEGylated polymeric micelles (IV)	Same as initial dose	7 days	Anti-PEG IgM
2012 (6)	Mice	PEGylated liposomal topotecan (IV)	Same as initial dose	7 days	Anti-PEG IgM
	Rat	PEGylated liposomal topotecan (IV)	Same as initial dose	7 days	Anti-PEG IgM
	Dog	PEGylated liposomal topotecan (IV)	Same as initial dose	7 days	Anti-PEG IgM
2015 (7)	Mice	PEGylated liposomes (IV)	Same as initial dose	6 days	Anti-PEG IgM
	Rat	PEGylated liposomes (IV)	Same as initial dose	6 days	Anti-PEG IgM
	Rabbit	PEGylated liposomes (IV)	Same as initial dose	6 days	Anti-PEG IgM
	Guinea pig	PEGylated liposomes (IV)	Same as initial dose	6 days	Anti-PEG IgM
2015 (11)	Mice	PEGylated ovalbumin (IV)	Same as initial dose	5 days	Anti-PEG IgM
2018 (16)	Rat	Homemade PEGylated microbubble (IV)	Same as initial dose	7 and 14 days	Anti-PEG IgM and IgG
	Rat	Definity®-PEGylated microbubble (IV)	Same as initial dose	7 and 14 days	Anti-PEG IgM and IgG
2019 (8)	Rat	PEGylated liposomal gambogenic acid (IV)	Same as initial dose	3, 5 and 7 days	Anti-PEG IgM
2020 (12)	Mice	Pegasys®-Peginterferon Alfa-2a (SC)	Same as initial dose	7 days	Anti-PEG IgM
	Mice	PEGylated ovalbumin (IV)	Pegasys®-Peginterferon Alfa-2a (SC)	7 days	Anti-PEG IgM
2020 (13)	Mice	Pegfilgrastim®-PEGylated human G-CSF (IV)	PEGylated liposomes (IV)	5 days	Anti-PEG IgM
	Mice	PEGylated ovalbumin (IV)	Pegfilgrastim®-PEGylated human G-CSF (IV)	5 days	Anti-PEG IgM
2021 (17)	Mice	PEGylated exosomes (IV)	Same as initial dose	5 days	Anti-PEG IgM
	Mice	PEGylated liposomes (IV)	PEGylated exosomes (IV)	5 days	Anti-PEG IgM
	Mice	PEGylated ovalbumin (IV)	PEGylated exosomes (IV)	5 days	Anti-PEG IgM
	Mice	PEGylated exosomes (IV)	PEGylated liposomes (IV)	5 days	Anti-PEG IgM
2023 (10)	Mice	PEG-based pharmaceutical excipients (IV)	PEGylated liposomes (IV)	7 days	Anti-PEG IgM and IgG
	Mice	PEG-based pharmaceutical excipients (IV)	PEGylated liposomal doxorubicin (IV)	7 days	Anti-PEG IgM and IgG
2023 (3)	Rat	PEGylated lipid nanoparticles (IM)	Same as initial dose	21 days	Anti-PEG IgM
2024 (9)	Mice	A cosmetic product containing PEG derivatives (TOP)	Doxil®-PEGylated liposomal doxorubicin (IV)	7 days	Anti-PEG IgM
	Mice	A cosmetic product containing PEG derivatives (TOP)	PEGylated liposomal oxaliplatin (IV)	7 days	Anti-PEG IgM

Abbreviations: IV, intravenous injection; SC, subcutaneous injection; IM, intramuscular injection; TOP, daily topical applications for 7 days; G-CSF, granulocyte colony-stimulating factor

Table S20. Clinical studies indicating accelerated blood clearance of PEGylated Drugs

Year[Ref]	Population	Clinical medication protocol					Correlation between treatment-induced anti-PEG antibodies and ABC phenomenon or reduced efficacy
		Drug	Dose	Administration route	Interval	Duration	
2006 (18)	13 patients with refractory gout	Krystexxa®[b]	4 ~ 24 mg	SC	Single injection	NA	Five out of thirteen patients developed low-titer anti-PEG IgM within 3-7 days of drug treatment. Subsequently, between 7-14 days, these patients exhibited low titers of anti-PEG IgG. These antibodies were associated with ABC phenomenon.
2007 (19)	28 children with acute lymphoblastic leukemia	Oncaspar®[a]	1000 U/m^2 for each treatment	IV	2 weeks	8 times	Among serum samples of 15 Oncaspar®-treated patients with undetectable ASNase activity at 0 to 15 days after the last dose, anti-PEG antibodies were detected in 9 samples by serology and 12 samples by flow cytometry. Anti-PEG antibody was detected by flow cytometry in 1 Oncaspar®-treated patient with lower ASNase activity. The presence of anti-PEG antibodies was very closely associated with rapid clearance of Oncaspar®.
2007 (20)	24 patients with refractory gout	Krystexxa®[b]	0.5 ~ 12 mg	IV	Single injection	NA	The relationship between pre-existing anti-PEG antibodies and the ABC phenomenon or reduced efficacy was not addressed in the article. Anti-PEG IgG was detected in serum samples of 9 gout patients, with drug clearance rates in these patients significantly accelerated.
2014 (21)	30 patients with refractory gout (including 7 organ transplant recipients)	Krystexxa®[b]	8 mg for each treatment	IV	3 weeks	5 times	Pre-existing anti-PEG IgG was detected in serum samples of 5 gout patients before Krystexxa® treatment, with drug clearance rates in these patients significantly accelerated. Anti-PEG IgG was detected in serum samples of 13 gout patients after Krystexxa® treatment, with drug clearance rates in these patients significantly accelerated.
2014 (22)	169 patients with refractory gout	Krystexxa®[b]	8 mg for each treatment	IV	2 weeks	12 times	Anti-PEG IgG and/or anti-PEG IgM were detected in serum samples of 67 gout patients after Krystexxa® treatment, with drug clearance rates in these patients significantly accelerated.
2018 (23)	24 patients with phenylketonuria	Palynziq®[c]	2.5 mg for each treatment	IV	1 week	Initiated for 4 times	Anti-PEG IgG and/or anti-PEG IgM were detected in serum samples of approximately 90% of phenylketonuria patients after Palynziq® treatment. These antibodies were associated with ABC phenomenon.
2022 (24)	889 children with acute lymphoblastic leukemia (ALL)	Oncaspar®[a]	Dose not described	IV	NA	NA	In pediatric subjects undergoing treatment with Oncaspar® for ALL, the presence of pre-existing anti-PEG IgG and anti-PEG IgM was associated with ABC phenomenon, reduced efficacy and hypersensitivity reactions.
2024 (25)	19 subjects receiving bivalent Moderna Spikevax® booster immunization	Spikevax®[d]	0.03 mg	IM	NA	1 time	Pre-existing anti-PEG antibodies was not significantly correlated with the clearance rate of Spikevax®. Treatment-induced anti-PEG antibodies was significantly correlated with the clearance rate of Spikevax®.

Abbreviations: IV, intravenous injection; SC, subcutaneous injection; NA, not applicable.

[a]Oncaspar® (pegaspargase), a polyethylene glycol (PEG)-conjugated form of Escherichia coli-derived L-asparaginase, was granted FDA approval in 1994 for the treatment of acute lymphoblastic leukemia.

[b]Krystexxa® (pegloticase), a polyethylene glycol (PEG)-conjugated form of recombinant porcine uricase, was granted FDA approval in 2010 for the treatment of refractory chronic gout.

[c]Palynziq® (pegvaliase), a polyethylene glycol (PEG)-conjugated form of recombinant [Anabaena variabilis] phenylalanine ammonia lyase, was granted FDA approval in 2018 for the treatment of adult phenylketonuria.

[d] Spikevax® (mRNA-1273), a lipid nanoparticle (LNP)-encapsulated messenger RNA (mRNA) encoding the spike protein of SARS-CoV-2, was granted FDA Emergency Use Authorization (EUA) in 2020 for the prevention of COVID-19 in individuals aged 18 years and older.

Figure S46. Screening of paired maternal and newborn serum samples negative for anti-PEG IgG and IgM

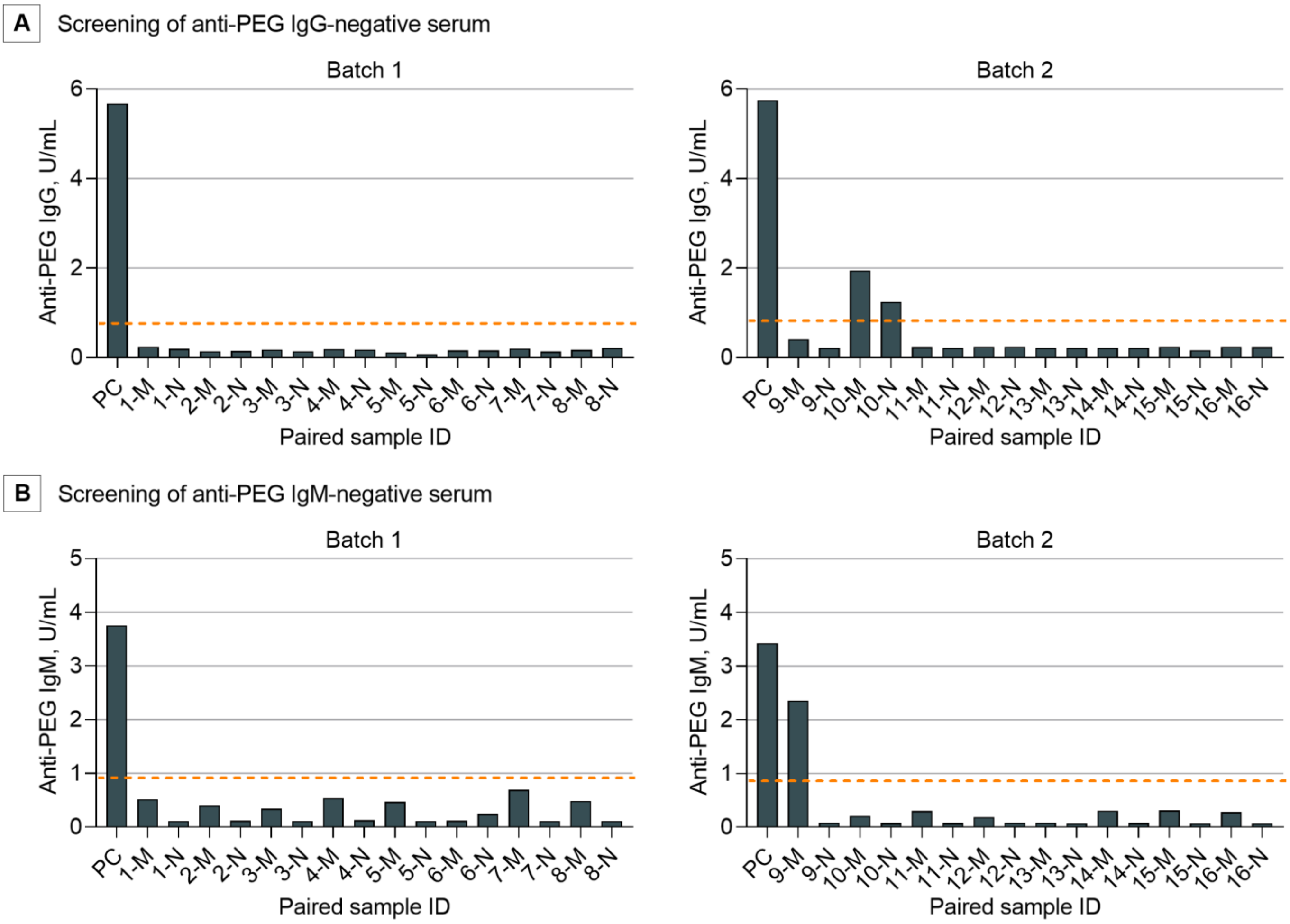

Abbreviations: PC, positive control included in commercial human anti-PEG IgM or IgG ELISA kits; M, maternal serum sample; N, newborn serum sample. Dotted lines indicate threshold values distinguishing between Positive/Negative in each ELISA for two independent batches.

Figure S47. Confirmation of the absence of anti-PEG IgE in maternal and newborn serum samples negative for anti-PEG IgG and IgM

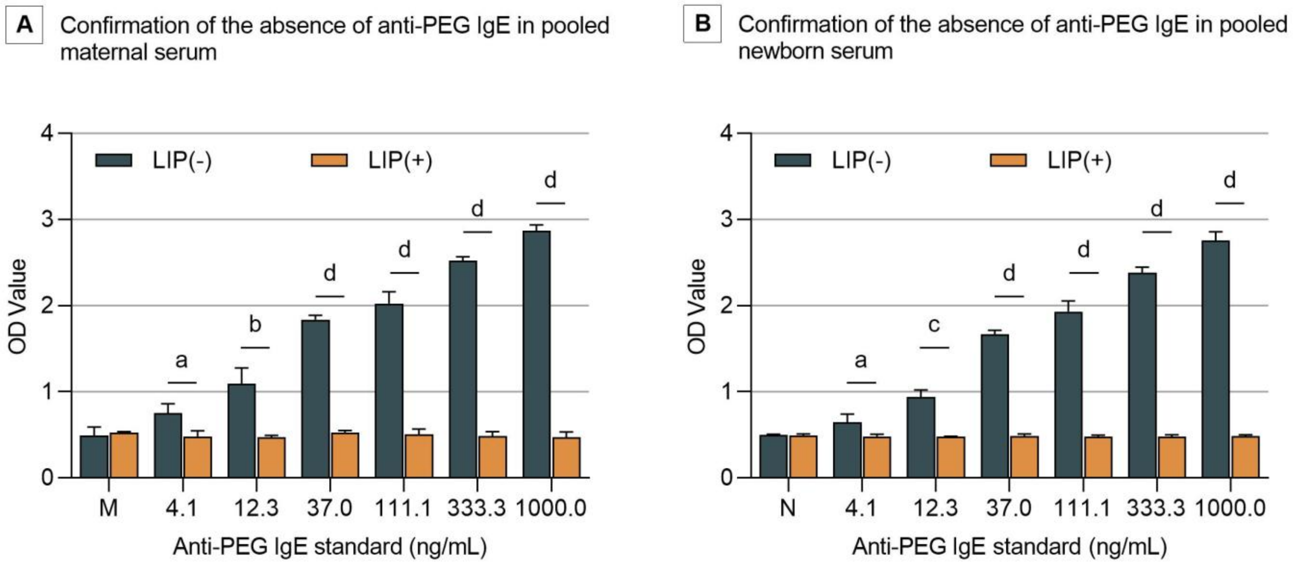

Abbreviations: LIP, PEGylated liposomal doxorubicin (competitors, see eMethods 4). LIP(-), the tested serum samples or anti-PEG IgE standard without the addition of LIP; LIP(+), the tested serum samples or anti-PEG IgE standard with the addition of LIP. M, pooled maternal serum samples negative for anti-PEG IgG and IgM; N, pooled newborn serum samples negative for anti-PEG IgG and IgM. All data were presented as "mean ± standard deviation" (n = 3), with differences between two groups analyzed using the two-tailed unpaired t-test. a, $P < 0.05$; b, $P < 0.01$; c, $P < 0.001$; d, $P < 0.0001$.

References

1. Chen BM, Su YC, Chang CJ, Burnouf PA, Chuang KH, Chen CH, et al. Measurement of pre-existing IgG and IgM antibodies against polyethylene glycol in healthy individuals. Anal Chem. 2016;88(21):10661-10666.
2. Frey A, Di Canzio J, Zurakowski D. A statistically defined endpoint titer determination method for Immunoassays. J Immunol Methods. 1998;221(1-2):35-41.
3. Wang HY, Wang YS, Yuan CZ, Xu X, Zhou WB, Huang YH, et al. Polyethylene glycol (PEG)-associated immune responses triggered by clinically relevant lipid nanoparticles in rats. NPJ Vaccines. 2023;8(1):169.
4. Ishida T, Ichihara M, Wang X, Yamamoto K, Kimura J, Majima E, et al. Injection of PEGylated liposomes in rats elicits PEG-specific IgM, which is responsible for rapid elimination of a second dose of PEGylated liposomes. J Control Release. 2006;112(1):15-25.
5. Wang XY, Ishida T, Kiwada H. Anti-PEG IgM elicited by injection of liposomes is involved in the enhanced blood clearance of a subsequent dose of PEGylated liposomes. J Control Release. 2007;119(2):236-244.
6. Li CL, Cao JN, Wang YJ, Zhao X, Deng CX, Wei N, et al. Accelerated blood clearance of pegylated liposomal topotecan: Influence of polyethylene glycol grafting density and animal species. J Pharm Sci. 2012;101(10):3864-3876.
7. Xu H, Ye FF, Hu MN, Yin PP, Zhang W, Li Y, et al. Influence of phospholipid types and animal models on the accelerated blood clearance phenomenon of PEGylated liposomes upon repeated injection. Drug Deliv. 2015;22(5):598-607.
8. Wang FL, Ye X, Wu YF, Wang HH, Sheng CM, Peng DY, et al. Time interval of two injections and first-dose dependent of accelerated blood clearance phenomenon induced by PEGylated liposomal gambogenic acid: the contribution of PEG-specific IgM. J Pharm Sci. 2019;108(1):641-651.
9. Gaballa SA, Shimizu T, Takata H, Ando H, Ibrahim M, Emam SE, et al. Impact of anti-PEG IgM induced via the topical application of a cosmetic product containing PEG derivatives on the antitumor effects of PEGylated liposomal antitumor drug formulations in mice. Mol Pharm. 2024;21(2):622-632.
10. Miao GF, He YJ, Lai K, Zhao Y, He PY, Tan GZ, et al. Accelerated blood clearance of PEGylated nanoparticles induced by PEG-based pharmaceutical excipients. J Control Release. 2023;363:12-26.
11. Mima Y, Hashimoto Y, Shimizu T, Kiwada H, Ishida T. Anti-PEG IgM is a major contributor to the accelerated blood clearance of polyethylene glycol-conjugated protein. Mol Pharma. 2015;12(7):2429-2435.
12. Elsadek NE, Hondo E, Shimizu T, Takata H, Abu Lila AS, Emam SE, et al. Impact of pre-existing or induced anti-PEG IgM on the pharmacokinetics of peginterferon Alfa-2a (Pegasys) in mice. Mol Pharma. 2020;17(8):2964-2970.
13. Elsadek NE, Lila ASA, Emam SE, Shimizu T, Takata H, Ando H, et al. Pegfilgrastim (PEG-G-CSF) induces anti-PEG IgM in a dose dependent manner and causes the accelerated blood clearance (ABC) phenomenon upon repeated administration in mice. Eur J Pharm Biopharm. 2020;152:56-62.
14. Ishihara T, Takeda M, Sakamoto H, Kimoto A, Kobayashi C, Takasaki N, et al. Accelerated blood clearance phenomenon upon repeated injection of PEG-modified PLA-nanoparticles. Pharm Res. 2009;26(10):2270-2279.
15. Koide H, Asai T, Kato H, Ando H, Shiraishi K, Yokoyama M, et al. Size-dependent induction of accelerated blood clearance phenomenon by repeated injections of polymeric micelles. Int J Pharm. 2012;432(1-2):75-79.
16. Fix SM, Nyankima AG, McSweeney MD, Tsuruta JK, Lai SK, Dayton PA. Accelerated clearance of ultrasound contrast agents containing polyethylene glycol is associated with the generation of anti-polyethylene glycol antibodies. Ultrasound Med Biol. 2018;44(6):1266-1280.
17. Emam SE, Elsadek NE, Abu Lila AS, Takata H, Kawaguchi Y, Shimizu T, et al. Anti-PEG IgM production and accelerated blood clearance phenomenon after the administration of PEGylated exosomes in mice. J Control Release. 2021;334:327-334.
18. Armstrong JK, Hempel G, Koling S, Chan LS, Fisher T, Meiselman HJ. Antibody against poly(ethylene glycol) adversely affects PEG-asparaginase therapy in acute lymphoblastic leukemia patients. Cancer. 2007;110(1):103-111.
19. Ganson NJ, Kelly SJ, Scarlett E, Sundy JS, Hershfield MS. Control of hyperuricemia in subjects with refractory gout, and induction of antibody against poly(ethylene glycol) (PEG), in a phase I trial of subcutaneous PEGylated urate oxidase. Arthritis Res Ther. 2006;8(1):R12.
20. Sundy JS, Ganson NJ, Kelly SJ, Scarlett EL, Rehrig CD, Huang W, et al. Pharmacokinetics and pharmacodynamics of intravenous PEGylated recombinant mammalian urate oxidase in patients with refractory gout. Arthritis Rheum. 2007;56(3):1021-1028.
21. Hershfield MS, Ganson NJ, Kelly SJ, Scarlett EL, Jaggers DA, Sundy JS. Induced and pre-existing anti-polyethylene glycol antibody in a trial of every 3-week dosing of pegloticase for refractory gout, including in organ transplant recipients. Arthritis Res Ther. 2014;16(2):R63.
22. Lipsky PE, Calabrese LH, Kavanaugh A, Sundy JS, Wright D, Wolfson M, et al. Pegloticase immunogenicity: the relationship between efficacy and antibody development in patients treated for refractory chronic gout. Arthritis Res Ther. 2014;16(2):R60.
23. Thomas J, Levy H, Amato S, Vockley J, Zori R, Dimmock D, et al. Pegvaliase for the treatment of phenylketonuria: Results of a long-term phase 3 clinical trial program (PRISM). Mol Genet Metab. 2018;124(1):27-38.
24. Khalil A, Würthwein G, Golitsch J, Hempel G, Fobker M, Gerss J, et al. Pre-existing antibodies against polyethylene glycol reduce asparaginase activities on first administration of pegylated asparaginase in children with acute lymphocytic leukemia. Haematologica. 2022;107(1):49-57.
25. Stephen JK, Li SY, Amarasena TH, Reynaldi A, Lee WS, Leeming MG, et al. Blood distribution of SARS-CoV-2 lipid nanoparticle mRNA vaccine in humans. ACS nano. 2024;18(39):27077-89.
26. Kozma GT, Mészáros T, Vashegyi I, Fülöp T, Örfi E, Dézsi L, et al. Pseudo-anaphylaxis to polyethylene glycol (PEG)-coated liposomes: Roles of anti-PEG IgM and complement activation in a porcine model of human infusion reactions. ACS Nano. 2019;13(8):9315-9324.
27. Stavnsbjerg C, Christensen E, Münter R, Henriksen JR, Fach M, Parhamifar L, et al. Accelerated blood clearance and hypersensitivity by PEGylated liposomes containing TLR agonists. J Control Release. 2022;342:337-344.
28. Chen WA, Chang DY, Chen BM, Lin YC, Barenholz Y, Roffler SR. Antibodies against poly(ethylene glycol) activate innate

immune cells and induce hypersensitivity reactions to PEGylated nanomedicines. ACS Nano. 2023;17(6):5757-5772.
29. Kozma GT, Mészáros T, Berényi P, Facskó R, Patkó Z, Oláh CZ, et al. Role of anti-polyethylene glycol (PEG) antibodies in the allergic reactions to PEG-containing Covid-19 vaccines: evidence for immunogenicity of PEG. Vaccine. 2023;41(31):4561-4570.
30. Ganson NJ, Povsic TJ, Sullenger BA, Alexander JH, Zelenkofske SL, Sailstad JM, et al. Pre-existing anti-polyethylene glycol antibody linked to first-exposure allergic reactions to pegnivacogin, a PEGylated RNA aptamer. J Allergy Clin Immunol. 2016;137(5):1610-1613.
31. Richter AW, Akerblom E. Polyethylene-glycol reactive antibodies in man - Titer distribution in allergic patients treated with monomethoxy polyethylene-glycol modified allergens or placebo, and in healthy blood-donors. Int Arch Allergy Appl Immunol. 1984;74(1):36-39.
32. Lubich C, Allacher P, de la Rosa M, Bauer A, Prenninger T, Horling FM, et al. The mystery of antibodies against polyethylene glycol (PEG) - What do we know? Pharm Res. 2016;33(9):2239-2249.
33. Yang Q, Jacobs TM, McCallen JD, Moore DT, Huckaby JT, Edelstein JN, et al. Analysis of pre-existing IgG and IgM antibodies against polyethylene glycol (PEG) in the general population. Anal Chem. 2016;88(23):11804-11812.
34. Fang JL, Beland FA, Tang YS, Roffler SR. Flow cytometry analysis of anti-polyethylene glycol antibodies in human plasma. Toxicol Rep. 2021;8:148-154.
35. Deuker MFS, Mailänder V, Morsbach S, Landfester K. Anti-PEG antibodies enriched in the protein corona of PEGylated nanocarriers impact the cell uptake. Nanoscale Horiz. 2023;8(10):1377-1385.
36. Li Z, Ma A, Miller I, Starnes R, Talkington A, Stone CA, et al. Development of anti-PEG IgG/IgM/IgE ELISA assays for profiling anti-PEG immunoglobulin response in PEG-sensitized individuals and patients with alpha-gal allergy. J Control Release. 2024;366:342-348.
37. Becker LC, Bergfeld WF, Belsito DV, Hill RA, Liebler DC, Shank RC, et al. Safety assessment of dimethicone crosspolymers as used in cosmetics. Int J Toxicol. 2014;33(2_suppl): 65S-115S.
38. Safety assessment of PEG stearates as used in cosmetics: CIR Expert Panel. 2024. https://www.cir-safety.org/sites/default/files/PEG Stearates.pdf?utm_source=chatgpt.com.
39. Safety assessment of PEG diesters as used in cosmetics: CIR Expert Panel. 2014. https://www.cir-safety.org/sites/default/files/peg_diesters.pdf?utm_source=chatgpt.com
40. Code of Federal Regulations Title 21, Sec. 176.210 - Defoaming agents used in the manufacture of paper and paperboard. (Explicitly lists Polyethylene glycol dilaurate): Food and Drug Administration. https://www.ecfr.gov/current/title-21/section-176.210
41. Code of Federal Regulations Title 21, Sec. 176.170 - Components of paper and paperboard in contact with aqueous and fatty foods: Food and Drug Administration. https://www.ecfr.gov/current/title-21/section-176.170
42. Code of Federal Regulations Title 21, Sec. 178.3910 - Surface lubricants used in the manufacture of metallic articles: Food and Drug Administration. https://www.ecfr.gov/current/title-21/section-178.3910
43. Commission Regulation (EU) No 10/2011 on plastic materials and articles intended to come into contact with food: European Union. https://eur-lex.europa.eu/eli/reg/2011/10/oj
44. Gaballa SA, Ando H, Ibrahim M, Amorim Matsuo NC, Naguib YW, Mady FM, et al. Impact of anti-PEG IgM induced via the topical application of a cosmetic product containing PEG derivatives on the antitumor effects of PEGylated liposomal antitumor drug formulations in mice. Mol Pharm. 2024;21(2):622-632.
45. Ibrahim M, Ando H, Elgarhy OH, Sarhan HA, Hussein AK, Shimizu T, et al. Investigation of anti-PEG antibody response to PEG-containing cosmetic products in mice. J Control Release. 2023;354:260-267.
46. Facskó R,Oláh CZ,Nagy A, Fülöp TG, Glatter KA, Radovits T, et al. Role of anti-polyethylene glycol (PEG) antibodies in the allergic reactions to PEG-containing Covid-19 vaccines: evidence for immunogenicity of PEG. Vaccine. 2023;41(31):4561-4570.
